# Species conservation profiles of endemic spiders (Araneae) from Madeira and Selvagens archipelagos, Portugal

**DOI:** 10.3897/BDJ.5.e20810

**Published:** 2017-10-18

**Authors:** Pedro Cardoso, Luís C Crespo, Isamberto Silva, Paulo AV Borges, Mário Boieiro

**Affiliations:** 1 Finnish Museum of Natural History, University of Helsinki, Helsinki, Finland; 2 IUCN SSC Spider & Scorpion Specialist Group, Helsinki, Finland; 3 Azorean Biodiversity Group/CE3C, University of the Azores, Angra do Heroismo, Portugal; 4 Biodiversity Research Institute UB, Departament Biologia Animal, Universitat de Barcelona, Barcelona, Spain; 5 Institute of Forests and Nature Conservation, Funchal, Portugal

**Keywords:** Arachnida, Arthropoda, extinction risk, islands, IUCN, Red List

## Abstract

**Background:**

The North Atlantic archipelagos of Madeira and Selvagens present a unique biological diversity including, presently, 56 endemic spider species. Several recent projects provide valuable information on their distribution across most islands and habitats. To date, the only endemic spider assessed according to the IUCN Red List criteria is *Hogna ingens.* The objective of this paper is to assess all remaining endemic species and advise on possible future conservation actions critical for the survival of endangered species.

**New information:**

Seven species were found to have a continuing decline in either range or population size. Their decline can be mostly attributed to habitat destruction or degradation, invasive plant species that reduce quality of habitat, forest fires at high mountain regions and possible competition for resources from invasive congeners. The tetragnathid *M. barreti* is considered as possibly extinct due to the suspected impact of a competing species. Although most endemic spiders from the Madeira and Selvagens archipelagos have relatively low extinction risk due to the good condition and protection of the laurisilva forests where many live, there are a number of species requiring urgent attention and protection measures. These include all cave and mountain-restricted species as well as those threatened by competing congeners or invasive plants. Extending current protected areas, restoring original habitats of threatened species and the control of invasive taxa should remain a priority for species survival.

## Introduction

**Figure 1.**
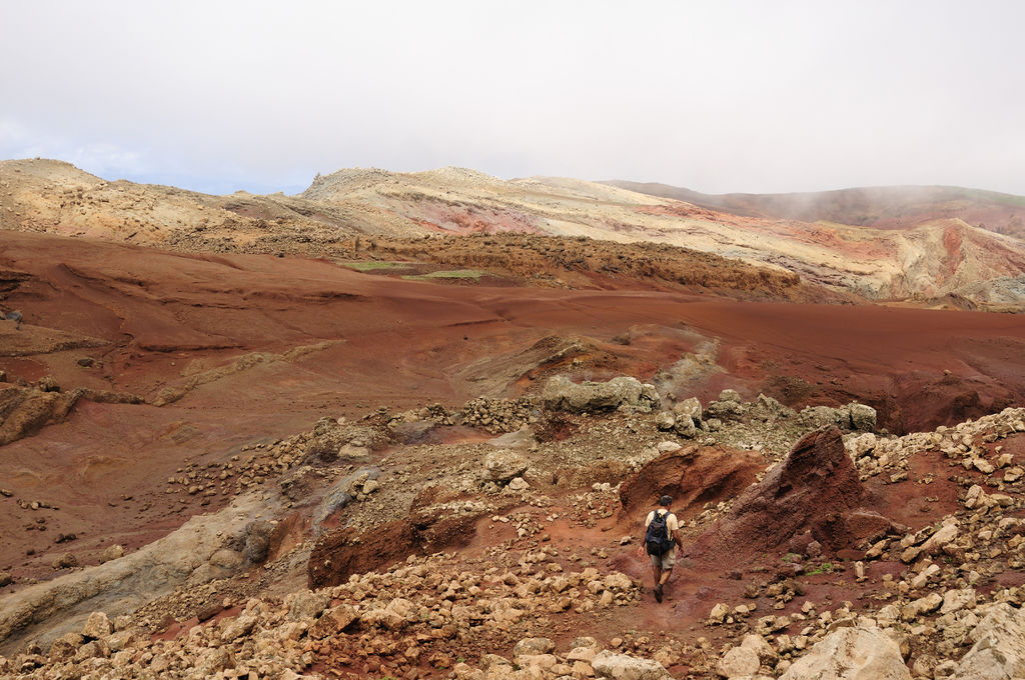
Barren area of Pedregal, Deserta Grande (photo by Pedro Cardoso).

**Figure 2.**
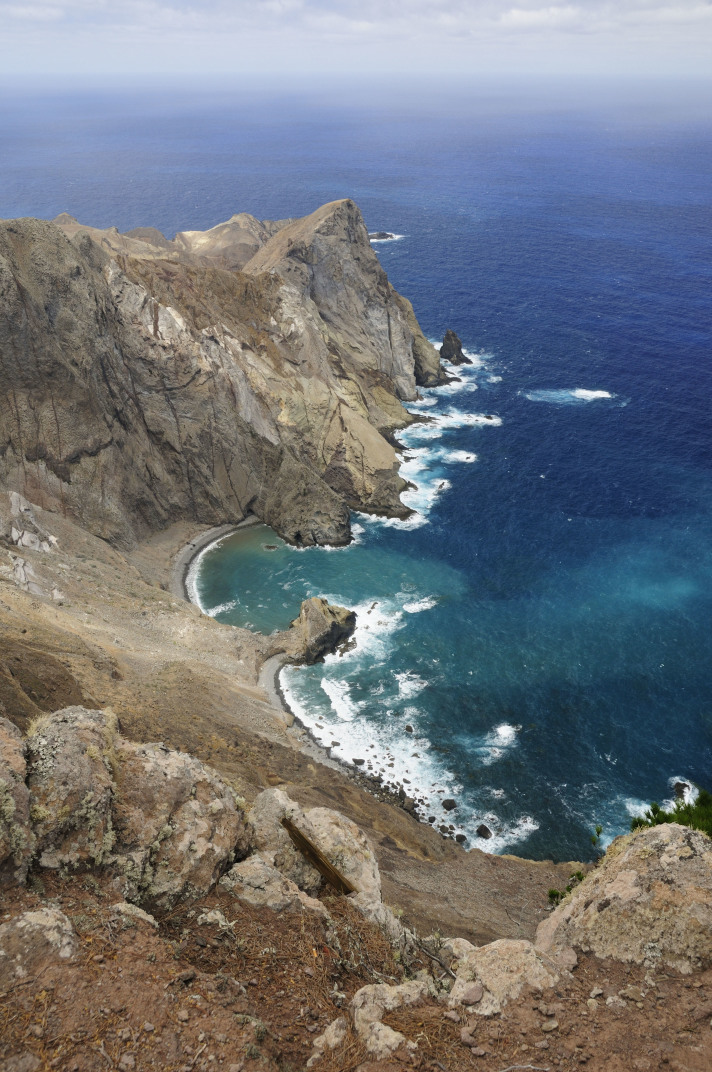
Coastal area near Pico Branco, Porto Santo (photo by Pedro Cardoso).

**Figure 3.**
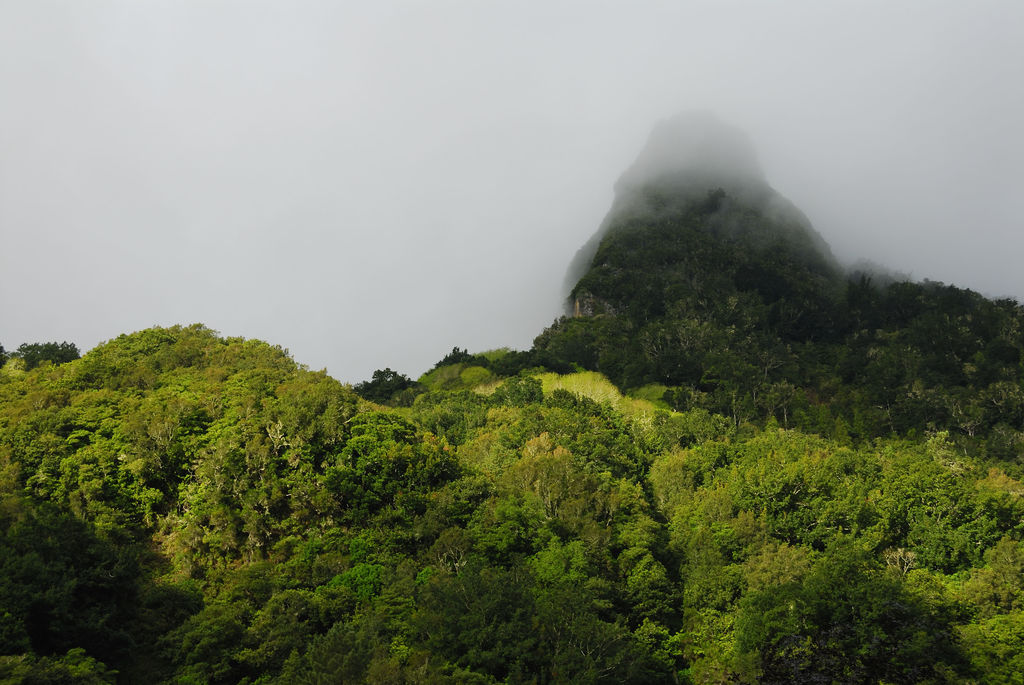
Laurisilva forest at Fajã da Nogueira, Madeira Island (photo by Pedro Cardoso).

**Figure 4.**
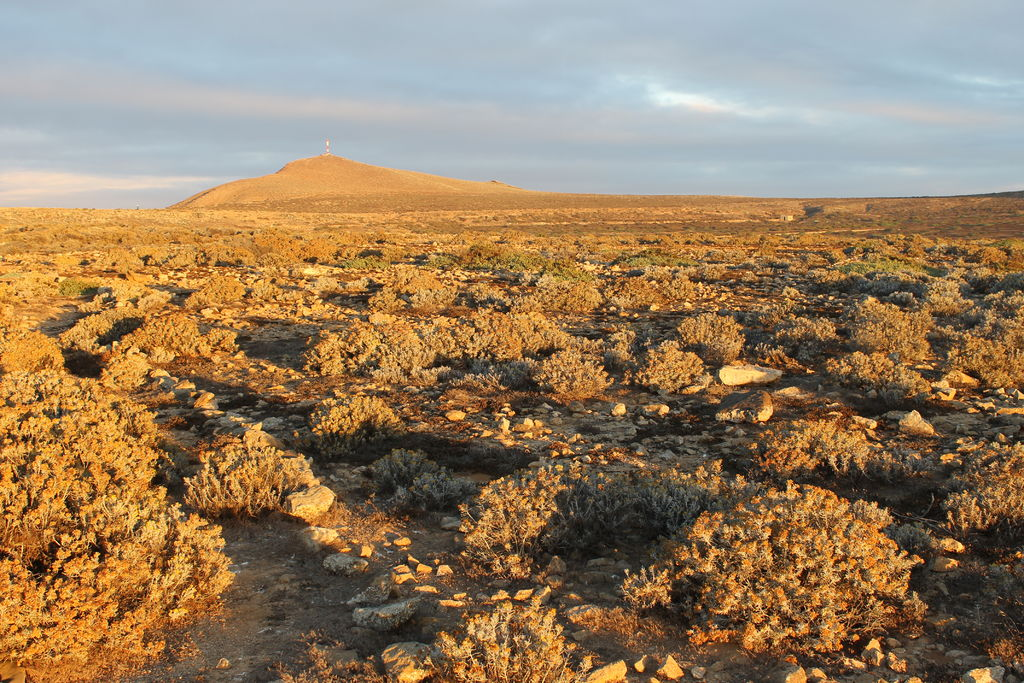
Selvagem Grande (photo by António Costa).

The archipelagos of Madeira and Selvagens are renown for presenting a unique biological diversity, which is a major contributing factor (together with threat levels) for their inclusion in one of the major biodiversity hotspots worldwide, jointly with two other Macaronesian archipelagos (Azores and the Canaries) (Myers et al. 2000). Both geological and geographical factors were important drivers of species richness as well as endemism in the Madeira and Selvagens archipelagos. The geological age of these islands (5-27 My) and their proximity to the mainland or to paleoislands allowed the colonization and diversification of many taxonomic groups (Fernández-Palacios 2010, Fernández-Palacios et al. 2011). Furthermore, the two archipelagos are composed by several islands and islets that share a number of species but also have a considerable number of exclusive species, single-island endemics (Borges et al. 2008). The Madeira archipelago comprises three island groups - the Desertas Islands (Deserta Grande - Fig. 1, Bugio and Ilhéu Chão); Porto Santo (including the main island - Fig. 2 - and seven islets); and Madeira proper (the main island - Fig. 3 - and the surrounding islets). The Selvagens archipelago is composed by one island and two islets – Selvagem Grande (Fig. 4), Selvagem Pequena and Ilhéu de Fora. It is however important to highlight the much larger contribution of Madeira Island to the overall species pool (both in terms of species richness and endemism) when compared with the other islands (Borges et al. 2008). Madeira Island is the largest, the highest and presents a more diverse set of terrestrial ecosystems, matching the stage of maximum orographic complexity of the volcanic island cycle (Fernández-Palacios et al. 2011).

The most emblematic ecosystem of the archipelago – the laurisilva forest – is restricted to Madeira Island where it covers nearly 20% of the land surface (Menezes et al. 2005, Boieiro et al. 2015). Madeira laurisilva is the largest area of this relict forest and includes some of the most pristine fragments being classified as a World Heritage Site by UNESCO (IUCN 1999), a priority habitat under the Habitats Directive and also benefiting from regional, national and international legislation (Menezes et al. 2005). Both the laurisilva forest and the summit vegetation harbour a large number of endemic species which made them preferential targets of biodiversity and taxonomic studies since the 19th century (Boieiro et al. 2010). The other islands, besides Madeira, are dominated by coastal scrublands and thermophilous woodland (particularly in Porto Santo) and have been less studied.

Recent efforts were made to update the knowledge on the taxonomy and conservation priorities for the archipelagos of Madeira and Selvagens (Martín et al. 2008, Silva et al. 2008, Martín et al. 2010). Over 7500 taxa were reported for these archipelagos, of which nearly 20% were endemics, mostly being terrestrial arthropods (Borges et al. 2008). The spider checklist was based on a thorough analysis of the literature plus personal observations and reported the occurrence of 184 species for these archipelagos, including 58 endemics (Cardoso and Crespo 2008). This publication also pointed to some gaps in the knowledge on species taxonomy and distribution that needed to be addressed in future studies. Several recent papers were important contributions to overcome those gaps by reporting new species to the archipelagos and providing valuable information on endemic species distribution (Crespo et al. 2009a, Crespo et al. 2009b, Crespo et al. 2013, Crespo et al. 2014a, Crespo et al. 2014b). Finally, a number of different projects (see acknowledgements) have made important contributions to overcoming these gaps by reporting extensive spider samples across islands and habitats during the last 10 years. These works led to important taxonomic and distribution data changes and we currently know 56 described endemic spider species.

Despite the known vulnerability of many endemic spider species, only the Desertas wolf spider - *Hogna ingens* (Blackwall, 1857), Fig. 5 - was assessed for extinction risk according to the Red List criteria of the International Union for the Conservation of Nature (Cardoso 2014). This large spider, restricted to Vale da Castanheira in the northern end of Deserta Grande, is threatened by *Phalaris* spp. grasses which, with their large roots, prevent the spider to access adequate shelters under stones and in soil crevices. A species conservation plan is now underway and includes regular monitoring of the spider population, chemical treatments to control the invasive species and *ex situ* conservation with possible future reintroduction of adult specimens.

The objective of this paper is to assess the remaining 55 endemic spider species according to the IUCN criteria and advise on possible future conservation actions critical for the survival of endangered species. In the future we intend to assess a number of species currently being described in order to contribute to the proper protection of this unique fauna.

## Methods

Species data were collected from all bibliography on Madeira and Selvagens spiders published until July 2017. These included mainly taxonomic and faunistic works. We also used numerous unpublished data collected within multiple projects (e.g. Boieiro et al. 2013, see also acknowledgements) that used standardized sampling (e.g. Cardoso 2009). This new information on species distribution will be published in several forthcoming papers. Whenever possible, with each species record we also collected additional information, namely habitat type and spatial error of coordinates.

For all analyses we used the R package red - IUCN redlisting tools (Cardoso 2017a, Cardoso 2017b). This package performs a number of spatial analyses based on either observed occurrences or estimated ranges. Functions include calculating Extent of Occurrence (EOO), Area of Occupancy (AOO), mapping species ranges, species distribution modelling using climate and land cover, calculating the Red List Index for groups of species, among others. The package also allows the calculation of confidence limits for all measures, an essential but almost invariably forgotten feature in view of unavoidable uncertainty. It outputs geographical range, elevation and country values, maps in several formats and vectorial data for visualization in Google Earth.

In this work, the EOO and AOO were calculated in one of two ways:

- for extremely range restricted species for which we assumed to know the full range, these values were classified as observed, the minimum convex polygon encompassing all observations used to calculate the EOO and the 2x2 km cells known to be occupied used to calculate the AOO. When the EOO was smaller than the AOO, it was made equal as per the IUCN guidelines (IUCN Standards and Petitions Subcommittee 2017).

- for widespread species or those for which we did not have confidence to know the full range, we performed species distribution modeling (SDM). This was done based on two environmental datasets depending on the distribution of each species. For single island endemics of Madeira proper (main island) we used 100x100m resolution data on altimetry, slope, annual precipitation, annual maximum and minimum relative humidity, annual maximum and minimum temperature, land cover and a disturbance index based on the latter (Boieiro et al. 2013, Cardoso et al. 2013). For species living on other islands, given the lack of availability of high-resolution data, we used 30 arc-second (approximately 1x1km) resolution data on 19 bioclimatic variables (Fick and Hijmans 2017) reduced to three after performing a PCA (the three first axes) plus land cover.

For SDMs we used ensemble modeling with the Maxent method (Phillips et al. 2006) implemented in red with associated spatial error (used to randomly place records within such error for each run), 100 runs per species and using only a subset of two explanatory variables for each run. Using subsets of explanatory variables was found to output better predictions than using many variables for rare species, with few occurrence records (Lomba et al. 2010, Breiner et al. 2015). Ensembles were summed using the Area Under the Curve (AUC) values to weight each of the 100 runs as:

weigth_run_ = max(0, (AUC_run_ - 0.5))^2^

Isolated patches outise the original distribution polygon were then excluded from maps to avoid overestimation of values. When performing SDMs, confidence limits for EOO and AOO were calculated using the number of models (runs) that predicted presence for each cell applying the percentiles 0.025 and 0.975 for the upper and lower limits respectively. All final maps and values were checked and validated by our own expert opinion. KMLs derived from these maps were also produced using the red package.

## Species Conservation Profiles

### Araneus hortensis

#### Species information

Scientific name: Araneus hortensis

Species authority: (Blackwall, 1859)

Kingdom: Animalia

Phylum: Arthropoda

Class: Arachnida

Order: Araneae

Family: Araneidae

Taxonomic notes: This species is not found since its description in 1859 (Blackwall 1859). Given the usually easy sampling of similar species, relatively large orb weavers, and the fact that the single specimen was captured in a garden, may be due to either it being a synonym of another, potentially common, species or an introduced species.

Region for assessment: Global

#### Geographic range

Biogeographic realm: Palearctic

Countries: Portugal

Map of records (Google Earth): Suppl. material 1

Basis of EOO and AOO: Unknown

Basis (narrative): This species EOO and AOO are unkown.

Range description: Only reference for the Island of Madeira, from unspecified locality (Blackwall 1859).

#### New occurrences

#### Extent of occurrence

EOO (km2): Unknown

Trend: Unknown

Causes ceased?: Unknown

Causes understood?: Unknown

Causes reversible?: Unknown

Extreme fluctuations?: Unknown

#### Area of occupancy

Trend: Unknown

Causes ceased?: Unknown

Causes understood?: Unknown

Causes reversible?: Unknown

Extreme fluctuations?: Unknown

AOO (km2): Unknown

#### Locations

Number of locations: Unknown

Trend: Unknown

Extreme fluctuations?: Unknown

#### Population

Number of individuals: Unknown

Trend: Unknown

Causes ceased?: Unknown

Causes understood?: Unknown

Causes reversible?: Unknown

Extreme fluctuations?: Unknown

Population Information (Narrative): The population size and trend are unknown.

#### Subpopulations

Trend: Unknown

Extreme fluctuations?: Unknown

Severe fragmentation?: Unknown

#### Habitat

System: Terrestrial

Habitat specialist: Unknown

Habitat (narrative): The habitat is unknown, the only specimen was captured in a garden at 200m altitude (Blackwall 1859).

Trend in extent, area or quality?: Unknown

##### Habitat

Habitat importance: Suitable

Habitats: 14.4. Artificial/Terrestrial - Rural Gardens

#### Habitat

Habitat importance: Suitable

Habitats: 14.4. Artificial/Terrestrial - Rural Gardens

#### Ecology

Size: 5 mm

Generation length (yr): 1

Dependency of single sp?: Unknown

Ecology and traits (narrative): The ecology of the species is unknown. The species and family are orb weavers feeding mostly on flying insects.

#### Threats

Justification for threats: Unknown threats.

##### Threats

Threat type: Past

Threats: 12. Other options - Other threat

#### Threats

Threat type: Past

Threats: 12. Other options - Other threat

#### Conservation

##### Conservation actions

#### Conservation actions

#### Other

##### Use and trade

Use type: International

##### Ecosystem services

Ecosystem service type: Less important

##### Research needed

Research needed: 1.1. Research - Taxonomy1.2. Research - Population size, distribution & trends1.3. Research - Life history & ecology1.5. Research - Threats

Justification for research needed: The species has not been found since original description in 1859 (Blackwall 1859) and needs, first of all, taxonomic clarification. If valid, basic information would be needed on its distribution, ecology and possible threats.

#### Use and trade

Use type: International

#### Ecosystem services

Ecosystem service type: Less important

#### Research needed

Research needed: 1.1. Research - Taxonomy1.2. Research - Population size, distribution & trends1.3. Research - Life history & ecology1.5. Research - Threats

Justification for research needed: The species has not been found since original description in 1859 (Blackwall 1859) and needs, first of all, taxonomic clarification. If valid, basic information would be needed on its distribution, ecology and possible threats.

#### Viability analysis

### Arctosa maderana

#### Species information

Scientific name: Arctosa maderana

Species authority: Roewer, 1960

Kingdom: Animalia

Phylum: Arthropoda

Class: Arachnida

Order: Araneae

Family: Lycosidae

Region for assessment: Global

#### Geographic range

Biogeographic realm: Palearctic

Countries: Portugal

Map of records (Google Earth): Suppl. material 2

Basis of EOO and AOO: Unknown

Basis (narrative): This species EOO and AOO are unknown.

Range description: Recorded from both Madeira Island and Porto Santo, from unspecified localities (Roewer 1960, Schmidt 1990).

#### New occurrences

#### Extent of occurrence

EOO (km2): Unknown

Trend: Unknown

Causes ceased?: Unknown

Causes understood?: Unknown

Causes reversible?: Unknown

Extreme fluctuations?: Unknown

#### Area of occupancy

Trend: Unknown

Causes ceased?: Unknown

Causes understood?: Unknown

Causes reversible?: Unknown

Extreme fluctuations?: Unknown

AOO (km2): Unknown

#### Locations

Number of locations: Unknown

Trend: Unknown

Extreme fluctuations?: Unknown

#### Population

Number of individuals: Unknown

Trend: Unknown

Causes ceased?: Unknown

Causes understood?: Unknown

Causes reversible?: Unknown

Extreme fluctuations?: Unknown

Population Information (Narrative): The population size and trend are unknown.

#### Subpopulations

Trend: Unknown

Extreme fluctuations?: Unknown

Severe fragmentation?: Unknown

#### Habitat

System: Terrestrial

Habitat specialist: Unknown

Habitat (narrative): There is poor information on species habitat, although the second record was from a sandy beach (Schmidt 1990).

Trend in extent, area or quality?: Unknown

##### Habitat

Habitat importance: Major Importance

Habitats: 12.2. Marine Intertidal - Sandy Shoreline and/or Beaches, Sand Bars, Spits, Etc

#### Habitat

Habitat importance: Major Importance

Habitats: 12.2. Marine Intertidal - Sandy Shoreline and/or Beaches, Sand Bars, Spits, Etc

#### Ecology

Size: 13-15 mm

Generation length (yr): 1

Dependency of single sp?: Unknown

Ecology and traits (narrative): The species ecology is unknown. Species of the same family and genus are active epigean hunters of insects and other arthropods.

#### Threats

Justification for threats: Unknown threats.

##### Threats

Threat type: Past

Threats: 12. Other options - Other threat

#### Threats

Threat type: Past

Threats: 12. Other options - Other threat

#### Conservation

##### Conservation actions

Conservation action type: In Place

#### Conservation actions

Conservation action type: In Place

#### Other

##### Use and trade

Use type: International

##### Ecosystem services

Ecosystem service type: Very important

##### Research needed

Research needed: 1.1. Research - Taxonomy1.2. Research - Population size, distribution & trends1.3. Research - Life history & ecology1.5. Research - Threats

Justification for research needed: Adults of this species have not been found since the original description (Roewer 1960) and it needs, first of all, taxonomic clarification. If valid, basic information would be needed on its distribution, ecology and possible threats.

#### Use and trade

Use type: International

#### Ecosystem services

Ecosystem service type: Very important

#### Research needed

Research needed: 1.1. Research - Taxonomy1.2. Research - Population size, distribution & trends1.3. Research - Life history & ecology1.5. Research - Threats

Justification for research needed: Adults of this species have not been found since the original description (Roewer 1960) and it needs, first of all, taxonomic clarification. If valid, basic information would be needed on its distribution, ecology and possible threats.

#### Viability analysis

### Centromerus anoculus

#### Species information

Scientific name: Centromerus anoculus

Species authority: Wunderlich, 1995

Common names: Aranha-cavernícola-de-São-Vicente

Kingdom: Animalia

Phylum: Arthropoda

Class: Arachnida

Order: Araneae

Family: Linyphiidae

Taxonomic notes: Possible junior synonym of *Centromerus sexoculatus* Wunderlich, 1992 (Reboleira et al. 2011) which if true could considerably impact the species extinction risk assessment.

Region for assessment: Global

#### Geographic range

Biogeographic realm: Palearctic

Countries: Portugal

Map of records (Google Earth): Suppl. material 3

Basis of EOO and AOO: Observed

Basis (narrative): Known from two lava tube systems (Wunderlich 1995, Reboleira et al. 2011). Given the relative scarcity and small size of caves in Madeira, this could correspond to the entire range of the species although, as noted above, it probably is a junior synonym of *C. sexoculatus*.

Min Elevation/Depth (m): 100

Max Elevation/Depth (m): 150

Range description: Only found in the lava tubes of Gruta dos Cardais in São Vicente, on northern Madeira Island, and Furnas do Cavalum in Machico, on eastern Madeira Island. Gruta dos Cardais is part of the largest known cave system in Madeira. The neighboring Grutas de São Vicente, which probably were once habitat for the species, are now in large part converted to show caves with extensive modifications in microclimate, including artificial lighting, water reservoirs and even newly built tunnels.

#### New occurrences

#### Extent of occurrence

EOO (km2): 8

Trend: Decline (inferred)

Justification for trend: No decrease in EOO has been registered but it is inferred from decline in habitat quality.

Causes ceased?: No

Causes understood?: Yes

Causes reversible?: Yes

Extreme fluctuations?: No

#### Area of occupancy

Trend: Decline (inferred)

Justification for trend: No decrease in AOO was observed, but it is inferred from decline in habitat quality.

Causes ceased?: No

Causes understood?: Yes

Causes reversible?: Yes

Extreme fluctuations?: No

AOO (km2): 8

#### Locations

Number of locations: 2

Justification for number of locations: Two locations, Gruta dos Cardais and Furnas do Cavalum, are threatened by uncontrolled visits, accumulation of litter and, in the case of the first, being used as shelter for domestic animals (Reboleira et al. 2011). A potential location, Grutas de São Vicente, was probably lost in the 1990s to a touristic development.

Trend: Stable

Justification for trend: The possible third location was lost more than 10 years ago, meaning the current trend in number of locations is probably stable despite the impeding threats.

Extreme fluctuations?: No

#### Population

Number of individuals: Unknown

Trend: Decline (estimated)

Justification for trend: The current threats are believed to cause a decrease in the species population numbers in unknown rates.

Basis for decline: (c) a decline in area of occupancy, extent of occurrence and/or quality of habitat

Causes ceased?: No

Causes understood?: Yes

Causes reversible?: Yes

Extreme fluctuations?: Unknown

Population Information (Narrative): The uncontrolled visits by tourists and locals that think of caves as adventure playground, accumulation of litter and use by domestic animals cause major changes in the cave environment and consequent decrease in quality of habitat for the species. This is believed to be leading to a decrease in population numbers, although no monitoring is being made and the rates are unknown.

#### Subpopulations

Number of subpopulations: 2

Trend: Stable

Justification for trend: Only two subpopulations historically known.

Extreme fluctuations?: No

Severe fragmentation?: No

#### Habitat

System: Terrestrial

Habitat specialist: Yes

Habitat (narrative): Species known from two lava tube systems.

Trend in extent, area or quality?: Decline (inferred)

Justification for trend: The quality of habitat is inferred to be decreasing due to severe changes in the environment of caves.

##### Habitat

Habitat importance: Major Importance

Habitats: 7.1. Caves and Subterranean Habitats (non-aquatic) - Caves

#### Habitat

Habitat importance: Major Importance

Habitats: 7.1. Caves and Subterranean Habitats (non-aquatic) - Caves

#### Ecology

Size: 3 mm

Generation length (yr): 1

Dependency of single sp?: No

Ecology and traits (narrative): Ecology and traits are largely unknown as the only references to the species are from a short taxonomic description and a checklist. Yet, congeners are known to build sheet webs and this species might build them on cave walls on the hunt for insects.

#### Threats

Justification for threats: The species was probably driven away from part of its historical range by touristic activities that include digging of new tunnels, water regime modifications (artificial pools) and artificial lighting. Both current locations are threatened by use of caves by domestic animals, uncontrolled visits and accumulation of litter.

##### Threats

Threat type: Ongoing

Threats: 1.3. Residential & commercial development - Tourism & recreation areas2.3. Agriculture & aquaculture - Livestock farming & ranching6.1. Human intrusions & disturbance - Recreational activities7.2. Natural system modifications - Dams & water management/use7.3. Natural system modifications - Other ecosystem modifications

#### Threats

Threat type: Ongoing

Threats: 1.3. Residential & commercial development - Tourism & recreation areas2.3. Agriculture & aquaculture - Livestock farming & ranching6.1. Human intrusions & disturbance - Recreational activities7.2. Natural system modifications - Dams & water management/use7.3. Natural system modifications - Other ecosystem modifications

#### Conservation

Justification for conservation actions: Furnas do Cavalum are considered scientific patrimony by the "Plano Director Municipal" of Machico. Yet, this cave species would benefit from effective protection with adequate legislation of the two lava tube systems where it occurs with eventual restoration of natural conditions of the environment and recovery and re-introduction in the lost location. While this is not possible, or as an alternative, a strict code of conduct for touristic or other activities in caves should be enforced and both communication to the general public and training of touristic agents should be subject of a conservation plan.

##### Conservation actions

Conservation action type: Needed

Conservation actions: 1.1. Land/water protection - Site/area protection1.2. Land/water protection - Resource & habitat protection2.1. Land/water management - Site/area management2.3. Land/water management - Habitat & natural process restoration3.2. Species management - Species recovery3.3. Species management - Species re-introduction4.2. Education & awareness - Training4.3. Education & awareness - Awareness & communications5.1. Law & policy - Legislation5.3. Law & policy - Private sector standards & codes5.4. Law & policy - Compliance and enforcement

#### Conservation actions

Conservation action type: Needed

Conservation actions: 1.1. Land/water protection - Site/area protection1.2. Land/water protection - Resource & habitat protection2.1. Land/water management - Site/area management2.3. Land/water management - Habitat & natural process restoration3.2. Species management - Species recovery3.3. Species management - Species re-introduction4.2. Education & awareness - Training4.3. Education & awareness - Awareness & communications5.1. Law & policy - Legislation5.3. Law & policy - Private sector standards & codes5.4. Law & policy - Compliance and enforcement

#### Other

##### Use and trade

Use type: International

##### Ecosystem services

Ecosystem service type: Very important

##### Research needed

Research needed: 1.1. Research - Taxonomy1.2. Research - Population size, distribution & trends1.3. Research - Life history & ecology1.5. Research - Threats2.1. Conservation Planning - Species Action/Recovery Plan2.2. Conservation Planning - Area-based Management Plan3.1. Monitoring - Population trends3.4. Monitoring - Habitat trends

Justification for research needed: The taxonomical status of the species and possible synonymy with *C. sexoculatus* should be clarified. Research on both the species current population trend and the reasons for this is needed to know the real threat levels and how to minimize them. A species conservation plan and a management plan would improve its survival chances for the future.

#### Use and trade

Use type: International

#### Ecosystem services

Ecosystem service type: Very important

#### Research needed

Research needed: 1.1. Research - Taxonomy1.2. Research - Population size, distribution & trends1.3. Research - Life history & ecology1.5. Research - Threats2.1. Conservation Planning - Species Action/Recovery Plan2.2. Conservation Planning - Area-based Management Plan3.1. Monitoring - Population trends3.4. Monitoring - Habitat trends

Justification for research needed: The taxonomical status of the species and possible synonymy with *C. sexoculatus* should be clarified. Research on both the species current population trend and the reasons for this is needed to know the real threat levels and how to minimize them. A species conservation plan and a management plan would improve its survival chances for the future.

#### Viability analysis

Justification for probability: 

### Centromerus sexoculatus

#### Species information

Scientific name: Centromerus sexoculatus

Species authority: Wunderlich, 1992

Common names: Aranha-cavernícola-do-Machico

Kingdom: Animalia

Phylum: Arthropoda

Class: Arachnida

Order: Araneae

Family: Linyphiidae

Taxonomic notes: Possible senior synonymy with *Centromerus anoculus* Wunderlich, 1995 (Reboleira et al. 2011)

Region for assessment: Global

#### Geographic range

Biogeographic realm: Palearctic

Countries: Portugal

Map of records (Google Earth): Suppl. material 4

Basis of EOO and AOO: Observed

Basis (narrative): Only known from a single lava tube system (Wunderlich 1992). Given the relative scarcity and small size of caves in Madeira, this could correspond to the entire range of the species.

Min Elevation/Depth (m): 150

Max Elevation/Depth (m): 150

Range description: Only found in the lava tubes of Furnas do Cavalum, close to Machico on eastern Madeira Island (Wunderlich 1992), although, if the synonymy with the more widespread *C. anoculus* is confirmed, it occupies two cave systems (see assessment for the latter species).

#### New occurrences

#### Extent of occurrence

EOO (km2): 4

Trend: Decline (inferred)

Justification for trend: Possibly threatened by uncontrolled visits to the caves and accumulation of litter (Reboleira et al. 2011) which decrease the habitat quality and ability of the species to occupy its full historical range.

Causes ceased?: No

Causes understood?: Yes

Causes reversible?: Yes

Extreme fluctuations?: No

#### Area of occupancy

Trend: Decline (inferred)

Justification for trend: Possibly threatened by uncontrolled visits to the caves and accumulation of litter (Reboleira et al. 2011) which decrease the habitat quality and ability of the species to occupy its full historical range.

Causes ceased?: No

Causes understood?: Yes

Causes reversible?: Yes

Extreme fluctuations?: No

AOO (km2): 4

#### Locations

Number of locations: 1

Justification for number of locations: A single site is known for the species, which is currently under serious threat.

Trend: Stable

Justification for trend: The single location is the full known historical range.

Extreme fluctuations?: No

#### Population

Number of individuals: Unknown

Trend: Decline (inferred)

Justification for trend: The population size of this species is unknown. It is possibly threatened by uncontrolled visits to the caves and accumulation of litter (Reboleira et al. 2011) which decrease the habitat quality and ability of the species to occupy its full historical range.

Basis for decline: (c) a decline in area of occupancy, extent of occurrence and/or quality of habitat

Causes ceased?: No

Causes understood?: Yes

Causes reversible?: Yes

Extreme fluctuations?: Unknown

Population Information (Narrative): A single subpopulation exists of unknown size.

#### Subpopulations

Number of subpopulations: 1

Trend: Stable

Extreme fluctuations?: No

Severe fragmentation?: No

#### Habitat

System: Terrestrial

Habitat specialist: Yes

Habitat (narrative): Species known from a single lava tube system, Furnas do Cavalum, Machico.

Trend in extent, area or quality?: Stable

##### Habitat

Habitat importance: Major Importance

Habitats: 7.1. Caves and Subterranean Habitats (non-aquatic) - Caves

#### Habitat

Habitat importance: Major Importance

Habitats: 7.1. Caves and Subterranean Habitats (non-aquatic) - Caves

#### Ecology

Size: 2 mm

Generation length (yr): 1

Dependency of single sp?: No

Ecology and traits (narrative): This is a troglobiont species with considerable eye reduction, depigmentation and appendage elongation. Ecology and traits are largely unknown as the only reference to the species is from a short taxonomic description. However, congeners are known to build sheet webs and this species might build them on cave walls on the hunt for insects.

#### Threats

Justification for threats: This species is possibly threatened by uncontrolled visits to the caves and accumulation of litter (Reboleira et al. 2011) which decrease the habitat quality and ability of the species to occupy its full historical range.

##### Threats

Threat type: Ongoing

Threats: 6.1. Human intrusions & disturbance - Recreational activities9.4. Pollution - Garbage & solid waste

#### Threats

Threat type: Ongoing

Threats: 6.1. Human intrusions & disturbance - Recreational activities9.4. Pollution - Garbage & solid waste

#### Conservation

Justification for conservation actions: Furnas do Cavalum are considered scientific patrimony by the "Plano Director Municipal" of Machico. Yet, this cave species would benefit from effective protection with adequate legislation of the lava tube system where it occurs with eventual restoration of natural conditions of the environment and recovery of its population. It should also be formally protected by adequate legislation.

##### Conservation actions

Conservation action type: Needed

Conservation actions: 1.1. Land/water protection - Site/area protection1.2. Land/water protection - Resource & habitat protection2.3. Land/water management - Habitat & natural process restoration3.2. Species management - Species recovery5.1. Law & policy - Legislation5.4. Law & policy - Compliance and enforcement

#### Conservation actions

Conservation action type: Needed

Conservation actions: 1.1. Land/water protection - Site/area protection1.2. Land/water protection - Resource & habitat protection2.3. Land/water management - Habitat & natural process restoration3.2. Species management - Species recovery5.1. Law & policy - Legislation5.4. Law & policy - Compliance and enforcement

#### Other

##### Use and trade

Use type: International

##### Ecosystem services

Ecosystem service type: Very important

##### Research needed

Research needed: 1.1. Research - Taxonomy1.2. Research - Population size, distribution & trends1.3. Research - Life history & ecology2.1. Conservation Planning - Species Action/Recovery Plan2.2. Conservation Planning - Area-based Management Plan3.1. Monitoring - Population trends3.4. Monitoring - Habitat trends

Justification for research needed: The taxonomical status of the species and possible synonymy with *C. anoculus* should be clarified. Research on population trends and its causes is needed to know the real threat levels and how to minimize them. A species conservation plan and a management plan would improve its survival chances for the future.

#### Use and trade

Use type: International

#### Ecosystem services

Ecosystem service type: Very important

#### Research needed

Research needed: 1.1. Research - Taxonomy1.2. Research - Population size, distribution & trends1.3. Research - Life history & ecology2.1. Conservation Planning - Species Action/Recovery Plan2.2. Conservation Planning - Area-based Management Plan3.1. Monitoring - Population trends3.4. Monitoring - Habitat trends

Justification for research needed: The taxonomical status of the species and possible synonymy with *C. anoculus* should be clarified. Research on population trends and its causes is needed to know the real threat levels and how to minimize them. A species conservation plan and a management plan would improve its survival chances for the future.

#### Viability analysis

Justification for probability: 

### Centromerus variegatus

#### Species information

Scientific name: Centromerus variegatus

Species authority: Denis, 1962

Kingdom: Animalia

Phylum: Arthropoda

Class: Arachnida

Order: Araneae

Family: Linyphiidae

Region for assessment: Global

#### Geographic range

Biogeographic realm: Palearctic

Countries: Portugal

Map of records (Google Earth): Suppl. material 5

Basis of EOO and AOO: Species Distribution Model

Basis (narrative): Multiple collection sites are recorded for this species, mostly recent and in laurisilva forest (Denis 1962, Wunderlich 1987, Crespo et al. 2014b). It was possible to perform species distribution modeling to predict its potential range with confidence limits. See methods for details.

Min Elevation/Depth (m): 300

Max Elevation/Depth (m): 1850

Range description: *Centromerus variegatus* is known throughout the laurisilva forest that occupies about 20% of the island, mainly on the steep and humid northern slopes.

#### New occurrences

#### Extent of occurrence

EOO (km2): 256-432-716

Trend: Stable

Justification for trend: The preferred habitat of the species, humid laurisilva forest, is not experiencing a decline in area and the invasive species present seem not to affect the spider populations.

Causes ceased?: Yes

Causes understood?: Yes

Causes reversible?: Yes

Extreme fluctuations?: No

#### Area of occupancy

Trend: Stable

Justification for trend: The preferred habitat of the species, humid laurisilva forest, is not experiencing a decline in area and the invasive species present seem not to affect the spider populations.

Causes ceased?: Yes

Causes understood?: Yes

Causes reversible?: Yes

Extreme fluctuations?: No

AOO (km2): 232-432-716

#### Locations

Number of locations: 0

Justification for number of locations: No known threats to the species.

Trend: Stable

Extreme fluctuations?: No

#### Population

Number of individuals: Unknown

Trend: Stable

Justification for trend: The preferred habitat of the species, humid laurisilva forest, is not experiencing a decline in area and the invasive species present seem not to affect the spider population.

Causes ceased?: Yes

Causes understood?: Yes

Causes reversible?: Yes

Extreme fluctuations?: No

Population Information (Narrative): No population size estimates exist.

#### Subpopulations

Trend: Stable

Extreme fluctuations?: No

Severe fragmentation?: No

#### Habitat

System: Terrestrial

Habitat specialist: Yes

Habitat (narrative): Humid laurisilva forest on the northern slopes of Madeira Island.

Trend in extent, area or quality?: Stable

##### Habitat

Habitat importance: Major Importance

Habitats: 1.9. Forest - Subtropical/Tropical Moist Montane

#### Habitat

Habitat importance: Major Importance

Habitats: 1.9. Forest - Subtropical/Tropical Moist Montane

#### Ecology

Size: 2 mm

Generation length (yr): 1

Dependency of single sp?: No

Ecology and traits (narrative): This species is a sheet-web builder on the tree branches and under stones, feeding mainly on small insects. The species seems closely associated to the laurisilva forest.

#### Threats

Justification for threats: Unknown threats.

##### Threats

Threat type: Past

Threats: 12. Other options - Other threat

#### Threats

Threat type: Past

Threats: 12. Other options - Other threat

#### Conservation

Justification for conservation actions: Most of the species range lies inside the Madeira Natural Park.

##### Conservation actions

Conservation action type: In Place

Conservation actions: 1.1. Land/water protection - Site/area protection

#### Conservation actions

Conservation action type: In Place

Conservation actions: 1.1. Land/water protection - Site/area protection

#### Other

##### Use and trade

Use type: International

##### Ecosystem services

Ecosystem service type: Very important

##### Research needed

Research needed: 3.1. Monitoring - Population trends

Justification for research needed: Monitoring of population trends should be conducted to confirm species status.

#### Use and trade

Use type: International

#### Ecosystem services

Ecosystem service type: Very important

#### Research needed

Research needed: 3.1. Monitoring - Population trends

Justification for research needed: Monitoring of population trends should be conducted to confirm species status.

#### Viability analysis

### Ceratinopsis infuscata

#### Species information

Scientific name: Ceratinopsis infuscata

Species authority: (Denis, 1962)

Kingdom: Animalia

Phylum: Arthropoda

Class: Arachnida

Order: Araneae

Family: Linyphiidae

Region for assessment: Global

#### Geographic range

Biogeographic realm: Palearctic

Countries: Portugal

Map of records (Google Earth): Suppl. material 6

Basis of EOO and AOO: Species Distribution Model

Basis (narrative): Multiple collection sites are recorded for the species, mostly recent and in laurisilva forest (Denis 1962, Wunderlich 1987, Crespo et al. 2014b). It was possible to perform species distribution modeling to predict its potential range with confidence limits. See methods for details.

Min Elevation/Depth (m): 300

Max Elevation/Depth (m): 1650

Range description: *Ceratinopsis infuscata* is known throughout the laurisilva forest that occupies about 20% of the island, mainly the steep and humid northern slopes.

#### New occurrences

#### Extent of occurrence

EOO (km2): 181-360-640

Trend: Stable

Justification for trend: The preferred habitat of the species, humid laurisilva forest, is not experiencing any decline in area and the invasive species present should not affect the spider population.

Causes ceased?: Yes

Causes understood?: Yes

Causes reversible?: Yes

Extreme fluctuations?: No

#### Area of occupancy

Trend: Stable

Justification for trend: The preferred habitat of the species, humid laurisilva forest, is not experiencing any decline in area and the invasive species present should not affect the spider population.

Causes ceased?: Yes

Causes understood?: Yes

Causes reversible?: Yes

Extreme fluctuations?: No

AOO (km2): 120-352-640

#### Locations

Number of locations: 0

Justification for number of locations: No known threats to the species.

Trend: Stable

Extreme fluctuations?: No

#### Population

Number of individuals: Unknown

Trend: Stable

Justification for trend: The preferred habitat of the species, humid laurisilva forest, is not experiencing any decline in area and the invasive species present should not affect the spider population.

Causes ceased?: Yes

Causes understood?: Yes

Causes reversible?: Yes

Extreme fluctuations?: No

Population Information (Narrative): No population size estimates exist.

#### Subpopulations

Trend: Stable

Extreme fluctuations?: No

Severe fragmentation?: No

#### Habitat

System: Terrestrial

Habitat specialist: Yes

Habitat (narrative): Humid laurisilva forest on the northern slopes of Madeira Island.

Trend in extent, area or quality?: Stable

Justification for trend: The preferred habitat of the species, humid laurisilva forest, is not experiencing any decline in area and the invasive species present should not affect the spider population.

##### Habitat

Habitat importance: Major Importance

Habitats: 1.9. Forest - Subtropical/Tropical Moist Montane

#### Habitat

Habitat importance: Major Importance

Habitats: 1.9. Forest - Subtropical/Tropical Moist Montane

#### Ecology

Size: 2 mm

Generation length (yr): 1

Dependency of single sp?: No

Ecology and traits (narrative): Sheet-web builder of the canopy stratum, feeding on small insects.

#### Threats

Justification for threats: Unknown threats.

##### Threats

Threat type: Past

Threats: 12. Other options - Other threat

#### Threats

Threat type: Past

Threats: 12. Other options - Other threat

#### Conservation

Justification for conservation actions: Most of the species range is inside the Madeira Natural Park.

##### Conservation actions

Conservation action type: In Place

Conservation actions: 1.1. Land/water protection - Site/area protection

#### Conservation actions

Conservation action type: In Place

Conservation actions: 1.1. Land/water protection - Site/area protection

#### Other

##### Use and trade

Use type: International

##### Ecosystem services

Ecosystem service type: Very important

##### Research needed

Research needed: 3.1. Monitoring - Population trends

Justification for research needed: Monitoring of population trends should be conducted to confirm species status.

#### Use and trade

Use type: International

#### Ecosystem services

Ecosystem service type: Very important

#### Research needed

Research needed: 3.1. Monitoring - Population trends

Justification for research needed: Monitoring of population trends should be conducted to confirm species status.

#### Viability analysis

### Dipoenata longitarsis

#### Species information

Scientific name: Dipoenata longitarsis

Species authority: (Denis, 1962)

Kingdom: Animalia

Phylum: Arthropoda

Class: Arachnida

Order: Araneae

Family: Theridiidae

Region for assessment: Global

#### Geographic range

Biogeographic realm: Palearctic

Countries: Portugal

Map of records (Google Earth): Suppl. material 7

Basis of EOO and AOO: Species Distribution Model

Basis (narrative): Only four records for this species exist, mostly recent and all in laurisilva forest (Denis 1962, Crespo et al. 2014b). It was possible to perform species distribution modeling to predict its potential range with confidence limits. See methods for details.

Min Elevation/Depth (m): 250

Max Elevation/Depth (m): 1850

Range description: *Dipoenata longitarsis* is known from a few sites in laurisilva forest that occupies about 20% of the island, mainly on its steep and humid northern slopes.

#### New occurrences

#### Extent of occurrence

EOO (km2): 192-404-725

Trend: Stable

Justification for trend: The preferred habitat of the species, humid laurisilva forest, is not experiencing any decline in area and the invasive species present should not affect the spider population.

Causes ceased?: Yes

Causes understood?: Yes

Causes reversible?: Yes

Extreme fluctuations?: No

#### Area of occupancy

Trend: Stable

Justification for trend: The preferred habitat of the species, humid laurisilva forest, is not experiencing any decline in area and the invasive species present should not affect the spider population.

Causes ceased?: Yes

Causes understood?: Yes

Causes reversible?: Yes

Extreme fluctuations?: No

AOO (km2): 192-404-708

#### Locations

Number of locations: 0

Justification for number of locations: No known threats to the species.

Trend: Stable

Extreme fluctuations?: No

#### Population

Number of individuals: Unknown

Trend: Stable

Justification for trend: The preferred habitat of the species, humid laurisilva forest, is not experiencing any decline in area and the invasive species present should not affect the spider population.

Causes ceased?: Yes

Causes understood?: Yes

Causes reversible?: Yes

Extreme fluctuations?: No

Population Information (Narrative): No population size estimates exist.

#### Subpopulations

Trend: Stable

Extreme fluctuations?: No

Severe fragmentation?: No

#### Habitat

System: Terrestrial

Habitat specialist: Yes

Habitat (narrative): Humid laurisilva forest on the northern slopes of Madeira Island.

Trend in extent, area or quality?: Stable

Justification for trend: The preferred habitat of the species, humid laurisilva forest, is not experiencing any decline in area and the invasive species present should not affect the spider population.

##### Habitat

Habitat importance: Major Importance

Habitats: 1.9. Forest - Subtropical/Tropical Moist Montane

#### Habitat

Habitat importance: Major Importance

Habitats: 1.9. Forest - Subtropical/Tropical Moist Montane

#### Ecology

Size: 1.3 mm

Generation length (yr): 1

Dependency of single sp?: No

Ecology and traits (narrative): As other species in the genus, *D. longitarsis* may feed mostly on ants at ground level and low vegetation.

#### Threats

Justification for threats: Unknown threats.

##### Threats

Threat type: Past

Threats: 12. Other options - Other threat

#### Threats

Threat type: Past

Threats: 12. Other options - Other threat

#### Conservation

Justification for conservation actions: Most of the species range is inside the Madeira Natural Park.

##### Conservation actions

Conservation action type: In Place

Conservation actions: 1.1. Land/water protection - Site/area protection

#### Conservation actions

Conservation action type: In Place

Conservation actions: 1.1. Land/water protection - Site/area protection

#### Other

##### Use and trade

Use type: International

##### Ecosystem services

Ecosystem service type: Very important

##### Research needed

Research needed: 3.1. Monitoring - Population trends

Justification for research needed: Monitoring of population trends should be conducted to confirm species status.

#### Use and trade

Use type: International

#### Ecosystem services

Ecosystem service type: Very important

#### Research needed

Research needed: 3.1. Monitoring - Population trends

Justification for research needed: Monitoring of population trends should be conducted to confirm species status.

#### Viability analysis

### Drassodes rugichelis

#### Species information

Scientific name: Drassodes rugichelis

Species authority: Denis, 1962

Kingdom: Animalia

Phylum: Arthropoda

Class: Arachnida

Order: Araneae

Family: Gnaphosidae

Region for assessment: Global

#### Geographic range

Biogeographic realm: Palearctic

Countries: Portugal

Map of records (Google Earth): Suppl. material 8

Basis of EOO and AOO: Observed

Basis (narrative): Largely unknown, as there are only two records for the species (Denis 1962, Wunderlich 1992), both on open mountainous areas.

Min Elevation/Depth (m): 1400

Max Elevation/Depth (m): 1800

Range description: *Drassodes rugichelis* is known from two sites (Paúl da Serra and Pico do Cidrão), both on open mountain areas. The true range is however unknown and not possible to model with confidence.

#### New occurrences

#### Extent of occurrence

EOO (km2): Unknown

Trend: Unknown

Causes ceased?: Unknown

Causes understood?: Unknown

Causes reversible?: Unknown

Extreme fluctuations?: Unknown

#### Area of occupancy

Trend: Unknown

Causes ceased?: Unknown

Causes understood?: Unknown

Causes reversible?: Unknown

Extreme fluctuations?: Unknown

AOO (km2): Unknown

#### Locations

Number of locations: Unknown

Trend: Unknown

Extreme fluctuations?: No

#### Population

Number of individuals: Unknown

Trend: Unknown

Causes ceased?: Unknown

Causes understood?: Unknown

Causes reversible?: Unknown

Extreme fluctuations?: Unknown

Population Information (Narrative): No population size estimates exist.

#### Subpopulations

Trend: Unknown

Extreme fluctuations?: No

Severe fragmentation?: Unknown

#### Habitat

System: Terrestrial

Habitat specialist: Unknown

Habitat (narrative): The two only known sites for the species are in open, mountain areas.

Trend in extent, area or quality?: Unknown

##### Habitat

Habitat importance: Major Importance

Habitats: 4.7. Grassland - Subtropical/High Altitude6. Rocky areas (e.g. inland cliffs, mountain peaks)

#### Habitat

Habitat importance: Major Importance

Habitats: 4.7. Grassland - Subtropical/High Altitude6. Rocky areas (e.g. inland cliffs, mountain peaks)

#### Ecology

Size: 11-16 mm

Generation length (yr): 1

Dependency of single sp?: No

Ecology and traits (narrative): The ecology of this species is mostly unknown, but it is probable that they are active nocturnal hunters at ground level.

#### Threats

Justification for threats: The mountain areas of Madeira Island have been affected by recent wildfires, which might have affected this species.

##### Threats

Threat type: Ongoing

Threats: 7.1. Natural system modifications - Fire & fire suppression

#### Threats

Threat type: Ongoing

Threats: 7.1. Natural system modifications - Fire & fire suppression

#### Conservation

Justification for conservation actions: Part of the known species range is inside the Madeira Natural Park.

##### Conservation actions

Conservation action type: In Place

Conservation actions: 1.1. Land/water protection - Site/area protection

#### Conservation actions

Conservation action type: In Place

Conservation actions: 1.1. Land/water protection - Site/area protection

#### Other

##### Use and trade

Use type: International

##### Ecosystem services

Ecosystem service type: Very important

##### Research needed

Research needed: 1.2. Research - Population size, distribution & trends1.3. Research - Life history & ecology1.5. Research - Threats3.1. Monitoring - Population trends3.4. Monitoring - Habitat trends

Justification for research needed: The distribution of the species should be researched through extensive collections on the islands' mountainous areas above the tree line. Monitoring of population trends should be conducted in the future and the negative effects of possible threats, such as wildfires, should also be assessed.

#### Use and trade

Use type: International

#### Ecosystem services

Ecosystem service type: Very important

#### Research needed

Research needed: 1.2. Research - Population size, distribution & trends1.3. Research - Life history & ecology1.5. Research - Threats3.1. Monitoring - Population trends3.4. Monitoring - Habitat trends

Justification for research needed: The distribution of the species should be researched through extensive collections on the islands' mountainous areas above the tree line. Monitoring of population trends should be conducted in the future and the negative effects of possible threats, such as wildfires, should also be assessed.

#### Viability analysis

### Dysdera aneris

#### Species information

Scientific name: Dysdera aneris

Species authority: Macías−Hernández & Arnedo, 2010

Common names: Aranha-tenaz-das-Selvagens

Kingdom: Animalia

Phylum: Arthropoda

Class: Arachnida

Order: Araneae

Family: Dysderidae

Region for assessment: Global

#### Geographic range

Biogeographic realm: Palearctic

Countries: Portugal

Map of records (Google Earth): Suppl. material 9

Basis of EOO and AOO: Observed

Basis (narrative): The restricted distribution of the species allows to known its EOO and AOO with reasonable confidence.

Min Elevation/Depth (m): 0

Max Elevation/Depth (m): 160

Range description: The species is restricted to the small Selvagens archipelago that lies between Madeira and the Canary Islands, where it possibly occupies all islets - Selvagem Grande, Selvagem Pequena and Ilhéu de Fora (Macías-Hernández et al. 2010).

#### New occurrences

#### Extent of occurrence

EOO (km2): 15

Trend: Stable

Justification for trend: No current threats to the species.

Causes ceased?: Yes

Causes understood?: Yes

Causes reversible?: Yes

Extreme fluctuations?: No

#### Area of occupancy

Trend: Stable

Justification for trend: No current threats to the species.

Causes ceased?: Yes

Causes understood?: Yes

Causes reversible?: Yes

Extreme fluctuations?: No

AOO (km2): 12

#### Locations

Number of locations: 0

Justification for number of locations: No current threats to the species.

Trend: Stable

Justification for trend: No current threats to the species.

Extreme fluctuations?: No

#### Population

Number of individuals: Unknown

Trend: Stable

Justification for trend: No current threats to the species.

Causes ceased?: Yes

Causes understood?: Yes

Causes reversible?: Yes

Extreme fluctuations?: No

Population Information (Narrative): No population size estimates exist.

#### Subpopulations

Number of subpopulations: 3

Trend: Stable

Justification for trend: No current threats to the species.

Extreme fluctuations?: No

Severe fragmentation?: No

#### Habitat

System: Terrestrial

Habitat specialist: Unknown

Habitat (narrative): The Selvagens archipelago is dominated by barren areas with low herbaceous vegetation and rocky outcrops.

Trend in extent, area or quality?: Stable

Justification for trend: The natural vegetation of Selvagem Grande has been largely recovered by successful projects coordinated by the Madeira Natural Park devoted to the eradication of invasive species.

##### Habitat

Habitat importance: Major Importance

Habitats: 4.5. Grassland - Subtropical/Tropical Dry6. Rocky areas (e.g. inland cliffs, mountain peaks)

#### Habitat

Habitat importance: Major Importance

Habitats: 4.5. Grassland - Subtropical/Tropical Dry6. Rocky areas (e.g. inland cliffs, mountain peaks)

#### Ecology

Size: 10 mm

Generation length (yr): 1

Dependency of single sp?: No

Ecology and traits (narrative): This species is found across the islands living on barren and rocky areas. The diet of *D. aneris* is unknown, although most congeners are specialized hunters feeding on woodlice.

#### Threats

Justification for threats: Although not currently a threat, the invasive *D. crocata* has been previously found on Selvagem Grande in the past (Macías-Hernández et al. 2010). It is possible that a future re-introduction of the species might lead to competition for resources with *D. aneris* with unpredictable consequences, as was already suggested for the extinction of at least one endemic *Dysdera* in the Azores (Cardoso et al. 2010).

##### Threats

Threat type: Future

Threats: 8.1. Invasive and other problematic species, genes & diseases - Invasive non-native/alien species/diseases

#### Threats

Threat type: Future

Threats: 8.1. Invasive and other problematic species, genes & diseases - Invasive non-native/alien species/diseases

#### Conservation

Justification for conservation actions: The entire range of the species is within the Selvagens Nature Reserve.

##### Conservation actions

Conservation action type: In Place

Conservation actions: 1.1. Land/water protection - Site/area protection

#### Conservation actions

Conservation action type: In Place

Conservation actions: 1.1. Land/water protection - Site/area protection

#### Other

##### Use and trade

Use type: International

##### Ecosystem services

Ecosystem service type: Very important

##### Research needed

Research needed: 1.3. Research - Life history & ecology3.1. Monitoring - Population trends

Justification for research needed: Monitoring of population trends should be conducted to confirm this species status. In addition, some information on the species life history, namely feeding regimen, should be collected as it might be restricted to very few prey types with implications for its conservation.

#### Use and trade

Use type: International

#### Ecosystem services

Ecosystem service type: Very important

#### Research needed

Research needed: 1.3. Research - Life history & ecology3.1. Monitoring - Population trends

Justification for research needed: Monitoring of population trends should be conducted to confirm this species status. In addition, some information on the species life history, namely feeding regimen, should be collected as it might be restricted to very few prey types with implications for its conservation.

#### Viability analysis

### Dysdera coiffaiti

#### Species information

Scientific name: Dysdera coiffaiti

Species authority: Denis, 1962

Common names: Aranha-tenaz-de-Coiffait

Kingdom: Animalia

Phylum: Arthropoda

Class: Arachnida

Order: Araneae

Family: Dysderidae

Region for assessment: Global

#### Geographic range

Biogeographic realm: Palearctic

Countries: Portugal

Map of records (Google Earth): Suppl. material 10

Basis of EOO and AOO: Species Distribution Model

Basis (narrative): Multiple collection sites are recorded for the species, mostly recent and in laurisilva forest. It was possible to perform species distribution modeling to predict its potential range with confidence limits. See methods for details.

Min Elevation/Depth (m): 300

Max Elevation/Depth (m): 1850

Range description: *Dysdera coiffaiti* is known throughout the laurisilva forest that occupies about 20% of the island, mainly on its steep and humid northern slopes.

#### New occurrences

#### Extent of occurrence

EOO (km2): 141-398-780

Trend: Stable

Justification for trend: The preferred habitat of the species, humid laurisilva forest, is not declining in area and the invasive species present should not affect this spider's population.

Causes ceased?: Yes

Causes understood?: Yes

Causes reversible?: Yes

Extreme fluctuations?: No

#### Area of occupancy

Trend: Stable

Justification for trend: The preferred habitat of the species, humid laurisilva forest, is not experiencing any decline in area and the invasive species present should not affect this spider's population.

Causes ceased?: Yes

Causes understood?: Yes

Causes reversible?: Yes

Extreme fluctuations?: No

AOO (km2): 116-396-780

#### Locations

Number of locations: 0

Justification for number of locations: No known threats to the species.

Trend: Stable

Justification for trend: No known threats to the species

Extreme fluctuations?: No

#### Population

Number of individuals: Unknown

Trend: Stable

Justification for trend: No known threats to the species.

Causes ceased?: Yes

Causes understood?: Yes

Causes reversible?: Yes

Extreme fluctuations?: No

Population Information (Narrative): No population size estimates exist.

#### Subpopulations

Trend: Stable

Extreme fluctuations?: No

Severe fragmentation?: No

#### Habitat

System: Terrestrial

Habitat specialist: Yes

Habitat (narrative): Humid laurisilva forest on the northern slopes of Madeira Island.

Trend in extent, area or quality?: Stable

Justification for trend: The preferred habitat of the species, humid laurisilva forest, is not declining in area and the invasive species present should not affect this spider's population.

##### Habitat

Habitat importance: Major Importance

Habitats: 1.9. Forest - Subtropical/Tropical Moist Montane

#### Habitat

Habitat importance: Major Importance

Habitats: 1.9. Forest - Subtropical/Tropical Moist Montane

#### Ecology

Size: 9-17 mm

Generation length (yr): 1

Dependency of single sp?: No

Ecology and traits (narrative): Found across the laurisilva forest of Madeira Island, living on the soil. The diet of *D. coiffaiti* is unknown, although most congeners are specialized hunters feeding on woodlice.

#### Threats

Justification for threats: Unknown threats.

##### Threats

Threat type: Past

Threats: 12. Other options - Other threat

#### Threats

Threat type: Past

Threats: 12. Other options - Other threat

#### Conservation

Justification for conservation actions: Most of the species range is inside the Madeira Natural Park.

##### Conservation actions

Conservation action type: In Place

Conservation actions: 1.1. Land/water protection - Site/area protection

#### Conservation actions

Conservation action type: In Place

Conservation actions: 1.1. Land/water protection - Site/area protection

#### Other

##### Use and trade

Use type: International

##### Ecosystem services

Ecosystem service type: Very important

##### Research needed

Research needed: 3.1. Monitoring - Population trends

Justification for research needed: Monitoring of population trends should be conducted to confirm species status.

#### Use and trade

Use type: International

#### Ecosystem services

Ecosystem service type: Very important

#### Research needed

Research needed: 3.1. Monitoring - Population trends

Justification for research needed: Monitoring of population trends should be conducted to confirm species status.

#### Viability analysis

### Dysdera diversa

#### Species information

Scientific name: Dysdera diversa

Species authority: Blackwall, 1862

Common names: Aranha-tenaz-diversa

Kingdom: Animalia

Phylum: Arthropoda

Class: Arachnida

Order: Araneae

Family: Dysderidae

Region for assessment: Global

#### Geographic range

Biogeographic realm: Palearctic

Countries: Portugal

Map of records (Google Earth): Suppl. material 11

Basis of EOO and AOO: Species Distribution Model

Basis (narrative): A single record is published on the original description without a precise locality (Blackwall 1862). Two more records are recent and to be confirmed and were used on an attemp to model the species distribution.

Min Elevation/Depth (m): 800

Max Elevation/Depth (m): 1850

Range description: *Dysdera diversa* is known only from high altitude areas of laurisilva forest (above 800m).

#### New occurrences

#### Extent of occurrence

EOO (km2): 13-264-644

Trend: Stable

Justification for trend: Inferred to be stable as the preferred habitat area and quality is stable.

Causes ceased?: Yes

Causes understood?: Yes

Causes reversible?: Yes

Extreme fluctuations?: No

#### Area of occupancy

Trend: Stable

Justification for trend: Inferred to be stable as the preferred habitat area and quality is stable.

Causes ceased?: Yes

Causes understood?: Yes

Causes reversible?: Yes

Extreme fluctuations?: No

AOO (km2): 12-264-644

#### Locations

Number of locations: 0

Justification for number of locations: No known threats to the species.

Trend: Stable

Extreme fluctuations?: No

#### Population

Number of individuals: Unknown

Trend: Stable

Justification for trend: Possibly stable as the preferred habitat area and quality is stable.

Causes ceased?: Yes

Causes understood?: Yes

Causes reversible?: Yes

Extreme fluctuations?: No

Population Information (Narrative): No species abundance estimates exist

#### Subpopulations

Trend: Stable

Extreme fluctuations?: No

Severe fragmentation?: No

#### Habitat

System: Terrestrial

Habitat specialist: Yes

Habitat (narrative): High altitude laurisilva forest.

Trend in extent, area or quality?: Stable

Justification for trend: Preferred habitat area and quality is stable.

##### Habitat

Habitat importance: Major Importance

Habitats: 1.9. Forest - Subtropical/Tropical Moist Montane

#### Habitat

Habitat importance: Major Importance

Habitats: 1.9. Forest - Subtropical/Tropical Moist Montane

#### Ecology

Size: 8 mm

Generation length (yr): 1

Dependency of single sp?: No

Ecology and traits (narrative): The species is found on few places of the high-altitude laurisilva forest of Madeira Island, living on the soil. The diet of *D. diversa* is unknown, although most congeners are specialized hunters feeding on woodlice.

#### Threats

Justification for threats: Unknown threats.

##### Threats

Threat type: Past

Threats: 12. Other options - Other threat

#### Threats

Threat type: Past

Threats: 12. Other options - Other threat

#### Conservation

Justification for conservation actions: All the species range is inside the Madeira Natural Park.

##### Conservation actions

Conservation action type: In Place

Conservation actions: 1.1. Land/water protection - Site/area protection

#### Conservation actions

Conservation action type: In Place

Conservation actions: 1.1. Land/water protection - Site/area protection

#### Other

##### Use and trade

Use type: International

##### Ecosystem services

Ecosystem service type: Very important

##### Research needed

Research needed: 1.2. Research - Population size, distribution & trends3.1. Monitoring - Population trends

Justification for research needed: As few localities are known for the species, basic research on species distribution should be made. Monitoring of population trends should be conducted to confirm species status.

#### Use and trade

Use type: International

#### Ecosystem services

Ecosystem service type: Very important

#### Research needed

Research needed: 1.2. Research - Population size, distribution & trends3.1. Monitoring - Population trends

Justification for research needed: As few localities are known for the species, basic research on species distribution should be made. Monitoring of population trends should be conducted to confirm species status.

#### Viability analysis

### Dysdera portisancti

#### Species information

Scientific name: Dysdera portisancti

Species authority: Wunderlich, 1995

Common names: Aranha-tenaz-do-Porto-Santo

Kingdom: Animalia

Phylum: Arthropoda

Class: Arachnida

Order: Araneae

Family: Dysderidae

Region for assessment: Global

#### Geographic range

Biogeographic realm: Palearctic

Countries: Portugal

Map of records (Google Earth): Suppl. material 12

Basis of EOO and AOO: Observed

Basis (narrative): Only two subpopulations of this species are known. These are in close proximity, on the Island of Porto Santo, which has been extensively surveyed, therefore the entire current distribution of this species should be known.

Min Elevation/Depth (m): 100

Max Elevation/Depth (m): 320

Range description: The species seems to be restricted to the northeastern part of the island of Porto Santo. It was originally described from Pico Branco (Wunderlich 1995), the area with the most extensive and best preserved native vegetation on the island. A single juvenile was recently collected at the top of the neighbouring Pico do Facho, in a small area with few native plants (e.g. *Heberdenia excelsa*) within exotic pine and cedar trees.

#### New occurrences

#### Extent of occurrence

EOO (km2): 8

Trend: Decline (inferred)

Justification for trend: Although the species is not known to ever have occupied areas outside its current range, this is extremely small (EOO is in effect < 1km^2^) and restricted to two nearby peaks with very few remnant native vegetation, with most areas surrounding them being converted to agricultural fields now abandoned or exotic pine and cedar plantations.

Causes ceased?: No

Causes understood?: Yes

Causes reversible?: Yes

Extreme fluctuations?: No

#### Area of occupancy

Trend: Decline (inferred)

Justification for trend: The species is not known to ever have occupied areas outside its current range, this being extremely small (AOO is in effect < 1km^2^) and restricted to two peaks with very few remnant native vegetation. Most areas surrounding them were converted to agricultural fields now abandoned or exotic pine and cedar plantations.

Causes ceased?: No

Causes understood?: Yes

Causes reversible?: Yes

Extreme fluctuations?: No

AOO (km2): 8

#### Locations

Number of locations: 2

Justification for number of locations: The two peaks where the species is found are surrounded by exotic tree plantations with numerous invasive plant species. The species seems to be able to survive mostly among native vegetation but also in few sheltered sites with planted trees. Yet, the spread of invasive plants might jeopardize the subpopulations in these two peaks.

Trend: Stable

Justification for trend: Further subpopulations were almost certainly lost in nearby peaks (e.g. Moledo or Pico da Gandaia), but probably before the species description in 1995.

Extreme fluctuations?: No

#### Population

Number of individuals: Unknown

Trend: Decline (inferred)

Justification for trend: Inferred from possible decline in habitat quality (leading also to possible decline in EOO and AOO) due to the effects of invasive plant species that do not provide adequate shelter for the spider.

Basis for decline: (c) a decline in area of occupancy, extent of occurrence and/or quality of habitat

Causes ceased?: No

Causes understood?: Yes

Causes reversible?: Yes

Extreme fluctuations?: No

Population Information (Narrative): No population size estimates exist.

#### Subpopulations

Number of subpopulations: 2

Trend: Stable

Extreme fluctuations?: No

Severe fragmentation?: Yes

Justification for fragmentation: As only one juvenile individual (identified through clear somatic characters) was found in Pico do Facho despite intensive sampling, it is possible that this subpopulation is endangered due to loss of habitat quality in the near future. That would mean that only a single subpopulation (50%) in Pico Branco would be left.

#### Habitat

System: Terrestrial

Habitat specialist: Yes

Habitat (narrative): The species seems to be restricted to rocky or few native vegetation areas with *Erica platycodon* or *Heberdenia excelsa* often within exotic pine and cedar.

Trend in extent, area or quality?: Decline (estimated)

Justification for trend: Possible estimated decline in habitat quality (leading also to possible inferred decline in EOO and AOO) due to the effects of invasive plant species that do not provide adequate shelter for the spider.

##### Habitat

Habitat importance: Major Importance

Habitats: 3.8. Shrubland - Mediterranean-type Shrubby Vegetation6. Rocky areas (e.g. inland cliffs, mountain peaks)

#### Habitat

Habitat importance: Major Importance

Habitats: 3.8. Shrubland - Mediterranean-type Shrubby Vegetation6. Rocky areas (e.g. inland cliffs, mountain peaks)

#### Ecology

Size: 7 mm

Generation length (yr): 1

Dependency of single sp?: No

Ecology and traits (narrative): The species is found living within scarce patches of native vegetation. The diet of *D. portisancti* is unknown, although most congeners are specialized hunters feeding on woodlice.

#### Threats

Justification for threats: As the species seems to be able to survive mostly among native vegetation, probably due to difficulty in finding adequate shelter within other plant species, the spread of invasive plants might jeopardize its survival.

##### Threats

Threat type: Ongoing

Threats: 8.1. Invasive and other problematic species, genes & diseases - Invasive non-native/alien species/diseases

#### Threats

Threat type: Ongoing

Threats: 8.1. Invasive and other problematic species, genes & diseases - Invasive non-native/alien species/diseases

#### Conservation

Justification for conservation actions: Part of the original habitat (Pico Branco) is included in the Natura network, but both localities urgently need to be restored jointly with the neighbouring peaks. The spider would benefit from recovery and re-introduction to these new areas which should have been part of its historical range with possible ex-situ breeding for both re-introduction and raising awareness on its emperiled status.

##### Conservation actions

Conservation action type: In Place

Conservation actions: 1.1. Land/water protection - Site/area protection

##### Conservation actions

Conservation action type: Needed

Conservation actions: 1.1. Land/water protection - Site/area protection2.2. Land/water management - Invasive/problematic species control2.3. Land/water management - Habitat & natural process restoration3.2. Species management - Species recovery3.3. Species management - Species re-introduction3.4. Species management - Ex-situ conservation4.3. Education & awareness - Awareness & communications5.1. Law & policy - Legislation

#### Conservation actions

Conservation action type: In Place

Conservation actions: 1.1. Land/water protection - Site/area protection

#### Conservation actions

Conservation action type: Needed

Conservation actions: 1.1. Land/water protection - Site/area protection2.2. Land/water management - Invasive/problematic species control2.3. Land/water management - Habitat & natural process restoration3.2. Species management - Species recovery3.3. Species management - Species re-introduction3.4. Species management - Ex-situ conservation4.3. Education & awareness - Awareness & communications5.1. Law & policy - Legislation

#### Other

##### Use and trade

Use type: International

##### Ecosystem services

Ecosystem service type: Very important

##### Research needed

Research needed: 1.3. Research - Life history & ecology2.1. Conservation Planning - Species Action/Recovery Plan2.2. Conservation Planning - Area-based Management Plan3.1. Monitoring - Population trends3.4. Monitoring - Habitat trends

Justification for research needed: Monitoring of population and habitat trends should be conducted to confirm species status. In addition, some information on life history, namely feeding regimen, should be collected about the species, as it might be restricted to very few prey types with implications for its conservation. The species would benefit from a species conservation plan that would include recovery actions for both the spider and the habitat and a management plan for new protected areas to be created within its historical range.

#### Use and trade

Use type: International

#### Ecosystem services

Ecosystem service type: Very important

#### Research needed

Research needed: 1.3. Research - Life history & ecology2.1. Conservation Planning - Species Action/Recovery Plan2.2. Conservation Planning - Area-based Management Plan3.1. Monitoring - Population trends3.4. Monitoring - Habitat trends

Justification for research needed: Monitoring of population and habitat trends should be conducted to confirm species status. In addition, some information on life history, namely feeding regimen, should be collected about the species, as it might be restricted to very few prey types with implications for its conservation. The species would benefit from a species conservation plan that would include recovery actions for both the spider and the habitat and a management plan for new protected areas to be created within its historical range.

#### Viability analysis

### Dysdera vandeli

#### Species information

Scientific name: Dysdera vandeli

Species authority: Denis, 1962

Common names: Aranha-tenaz-de-Vandel

Kingdom: Animalia

Phylum: Arthropoda

Class: Arachnida

Order: Araneae

Family: Dysderidae

Region for assessment: Global

#### Geographic range

Biogeographic realm: Palearctic

Countries: Portugal

Map of records (Google Earth): Suppl. material 13

Basis of EOO and AOO: Unknown

Basis (narrative): Distribution of the species is unknown as there is only a single record from Caldeirão do Inferno in 1962 (Denis 1962). It has not been found since this despite recent survey efforts.

Min Elevation/Depth (m): 1500

Max Elevation/Depth (m): 1500

Range description: Distribution of the species is unknown as there is a single record from Caldeirao do Inferno in 1962 (Denis 1962).

#### New occurrences

#### Extent of occurrence

EOO (km2): Unknown

Trend: Unknown

Causes ceased?: Unknown

Causes understood?: Unknown

Causes reversible?: Unknown

Extreme fluctuations?: Unknown

#### Area of occupancy

Trend: Unknown

Causes ceased?: Unknown

Causes understood?: Unknown

Causes reversible?: Unknown

Extreme fluctuations?: Unknown

AOO (km2): Unknown

#### Locations

Number of locations: Unknown

Trend: Unknown

Extreme fluctuations?: Unknown

#### Population

Number of individuals: Unknown

Trend: Unknown

Causes ceased?: Unknown

Causes understood?: Unknown

Causes reversible?: Unknown

Extreme fluctuations?: Unknown

Population Information (Narrative): No population size estimates exist.

#### Subpopulations

Trend: Unknown

Extreme fluctuations?: Unknown

Severe fragmentation?: Unknown

#### Habitat

System: Terrestrial

Habitat specialist: Unknown

Habitat (narrative): Probably humid laurisilva forest on the northern slopes of Madeira Island.

Trend in extent, area or quality?: Unknown

##### Habitat

Habitat importance: Major Importance

Habitats: 1.9. Forest - Subtropical/Tropical Moist Montane

#### Habitat

Habitat importance: Major Importance

Habitats: 1.9. Forest - Subtropical/Tropical Moist Montane

#### Ecology

Size: 6 mm

Generation length (yr): 1

Dependency of single sp?: No

Ecology and traits (narrative): The diet of *D. vandeli* is unknown, although most congeners are specialized hunters feeding on woodlice.

#### Threats

Justification for threats: Unknown threats.

##### Threats

Threat type: Past

Threats: 12. Other options - Other threat

#### Threats

Threat type: Past

Threats: 12. Other options - Other threat

#### Conservation

Justification for conservation actions: The known species range is inside the Madeira Natural Park.

##### Conservation actions

Conservation action type: In Place

Conservation actions: 1.1. Land/water protection - Site/area protection

#### Conservation actions

Conservation action type: In Place

Conservation actions: 1.1. Land/water protection - Site/area protection

#### Other

##### Use and trade

Use type: International

##### Ecosystem services

Ecosystem service type: Very important

##### Research needed

Research needed: 1.1. Research - Taxonomy1.2. Research - Population size, distribution & trends1.3. Research - Life history & ecology1.5. Research - Threats

Justification for research needed: This species has not been found since the original description (Denis 1962) and it needs, first of all, taxonomic clarification. If valid, basic information would be needed on its distribution, ecology and possible threats.

#### Use and trade

Use type: International

#### Ecosystem services

Ecosystem service type: Very important

#### Research needed

Research needed: 1.1. Research - Taxonomy1.2. Research - Population size, distribution & trends1.3. Research - Life history & ecology1.5. Research - Threats

Justification for research needed: This species has not been found since the original description (Denis 1962) and it needs, first of all, taxonomic clarification. If valid, basic information would be needed on its distribution, ecology and possible threats.

#### Viability analysis

### Echemus modestus

#### Species information

Scientific name: Echemus modestus

Species authority: Kulczynski, 1899

Kingdom: Animalia

Phylum: Arthropoda

Class: Arachnida

Order: Araneae

Family: Gnaphosidae

Taxonomic notes: Not recorded since original description from Madeira with uncertain locality (Kulczyński 1899).

Region for assessment: Global

#### Geographic range

Biogeographic realm: Palearctic

Countries: Portugal

Map of records (Google Earth): Suppl. material 14

Basis of EOO and AOO: Unknown

Basis (narrative): The species EOO and AOO are unknown.

Range description: Only mentioned from Madeira Island, with no locality data.

#### New occurrences

#### Extent of occurrence

EOO (km2): Unknown

Trend: Unknown

Causes ceased?: Unknown

Causes understood?: Unknown

Causes reversible?: Unknown

Extreme fluctuations?: Unknown

#### Area of occupancy

Trend: Unknown

Causes ceased?: Unknown

Causes understood?: Unknown

Causes reversible?: Unknown

Extreme fluctuations?: Unknown

AOO (km2): Unknown

#### Locations

Number of locations: Unknown

Trend: Unknown

Extreme fluctuations?: Unknown

#### Population

Number of individuals: Unknown

Trend: Unknown

Causes ceased?: Unknown

Causes understood?: Unknown

Causes reversible?: Unknown

Extreme fluctuations?: Unknown

Population Information (Narrative): No population size estimates exist.

#### Subpopulations

Trend: Unknown

Extreme fluctuations?: Unknown

Severe fragmentation?: Unknown

#### Habitat

System: Terrestrial

Habitat specialist: Unknown

Habitat (narrative): The species habitat is unknown.

Trend in extent, area or quality?: Unknown

##### Habitat

Habitat importance: Major Importance

Habitats: 18. Unknown

#### Habitat

Habitat importance: Major Importance

Habitats: 18. Unknown

#### Ecology

Size: 4 mm

Generation length (yr): 1

Dependency of single sp?: No

Ecology and traits (narrative): If similar to other congeners, probably an active nocturnal hunter at ground level.

#### Threats

Justification for threats: Unknown threats.

##### Threats

Threat type: Past

Threats: 12. Other options - Other threat

#### Threats

Threat type: Past

Threats: 12. Other options - Other threat

#### Conservation

##### Conservation actions

#### Conservation actions

#### Other

##### Use and trade

Use type: International

##### Ecosystem services

Ecosystem service type: Very important

##### Research needed

Research needed: 1.1. Research - Taxonomy1.2. Research - Population size, distribution & trends1.3. Research - Life history & ecology1.5. Research - Threats

Justification for research needed: The species has not been found since original description in 1899 (Kulczyński 1899) and needs, first of all, taxonomic clarification. If valid, basic information would be needed on its distribution, ecology and possible threats.

#### Use and trade

Use type: International

#### Ecosystem services

Ecosystem service type: Very important

#### Research needed

Research needed: 1.1. Research - Taxonomy1.2. Research - Population size, distribution & trends1.3. Research - Life history & ecology1.5. Research - Threats

Justification for research needed: The species has not been found since original description in 1899 (Kulczyński 1899) and needs, first of all, taxonomic clarification. If valid, basic information would be needed on its distribution, ecology and possible threats.

#### Viability analysis

### Frontinellina dearmata

#### Species information

Scientific name: Frontinellina dearmata

Species authority: (Kulczynski, 1899)

Kingdom: Animalia

Phylum: Arthropoda

Class: Arachnida

Order: Araneae

Family: Linyphiidae

Region for assessment: Global

#### Geographic range

Biogeographic realm: Palearctic

Countries: Portugal

Map of records (Google Earth): Suppl. material 15

Basis of EOO and AOO: Species Distribution Model

Basis (narrative): Multiple collection sites are recorded for the species, mostly recent and in laurisilva forest (Kulczyński 1899, Crespo et al. 2014b). From previous data collection it was possible to perform species distribution modeling to predict its potential range with confidence limits. See methods for details.

Min Elevation/Depth (m): 100

Max Elevation/Depth (m): 1450

Range description: *Frontinellina dearmata* is known throughout the laurisilva forest that occupies about 20% of the island, mainly on its steep and humid northern slopes.

#### New occurrences

#### Extent of occurrence

EOO (km2): 53-296-736

Trend: Stable

Justification for trend: The preferred habitat of the species, humid laurisilva forest, is not experiencing any decline in area and the invasive species present should not affect the spider population.

Causes ceased?: Yes

Causes understood?: Yes

Causes reversible?: Yes

Extreme fluctuations?: No

#### Area of occupancy

Trend: Stable

Justification for trend: The preferred habitat of the species, humid laurisilva forest, is not experiencing any decline in area and the invasive species present should not affect the spider population.

Causes ceased?: Yes

Causes understood?: Yes

Causes reversible?: Yes

Extreme fluctuations?: No

AOO (km2): 52-296-732

#### Locations

Number of locations: 0

Justification for number of locations: No known threats to the species.

Trend: Stable

Extreme fluctuations?: No

#### Population

Number of individuals: Unknown

Trend: Stable

Justification for trend: The preferred habitat of the species, humid laurisilva forest, is not experiencing any decline in area and the invasive species present should not affect the spider population.

Causes ceased?: Yes

Causes understood?: Yes

Causes reversible?: Yes

Extreme fluctuations?: No

Population Information (Narrative): No population size estimates exist.

#### Subpopulations

Trend: Stable

Extreme fluctuations?: No

Severe fragmentation?: No

#### Habitat

System: Terrestrial

Habitat specialist: Yes

Habitat (narrative): Humid laurisilva forest on the northern slopes of Madeira Island.

Trend in extent, area or quality?: Stable

##### Habitat

Habitat importance: Major Importance

Habitats: 1.9. Forest - Subtropical/Tropical Moist Montane

#### Habitat

Habitat importance: Major Importance

Habitats: 1.9. Forest - Subtropical/Tropical Moist Montane

#### Ecology

Size: 5 mm

Generation length (yr): 1

Dependency of single sp?: No

Ecology and traits (narrative): Sheet-web builder at the canopy of native trees feeding mainly on small insects.

#### Threats

Justification for threats: Unknown threats.

##### Threats

Threat type: Past

Threats: 12. Other options - Other threat

#### Threats

Threat type: Past

Threats: 12. Other options - Other threat

#### Conservation

Justification for conservation actions: Most of the species range is inside the Madeira Natural Park.

##### Conservation actions

Conservation action type: In Place

Conservation actions: 1.1. Land/water protection - Site/area protection

#### Conservation actions

Conservation action type: In Place

Conservation actions: 1.1. Land/water protection - Site/area protection

#### Other

##### Use and trade

Use type: International

##### Ecosystem services

Ecosystem service type: Very important

##### Research needed

Research needed: 3.1. Monitoring - Population trends

Justification for research needed: Monitoring of population trends should be conducted to confirm species status.

#### Use and trade

Use type: International

#### Ecosystem services

Ecosystem service type: Very important

#### Research needed

Research needed: 3.1. Monitoring - Population trends

Justification for research needed: Monitoring of population trends should be conducted to confirm species status.

#### Viability analysis

### Frontiphantes fulgurenotatus

#### Species information

Scientific name: Frontiphantes fulgurenotatus

Species authority: (Schenkel, 1938)

Kingdom: Animalia

Phylum: Arthropoda

Class: Arachnida

Order: Araneae

Family: Linyphiidae

Region for assessment: Global

#### Geographic range

Biogeographic realm: Palearctic

Countries: Portugal

Map of records (Google Earth): Suppl. material 16

Basis of EOO and AOO: Species Distribution Model

Basis (narrative): Multiple collection sites have been recorded for the species, mostly recent and in laurisilva forest (Schenkel 1938, Denis 1962, Wunderlich 1987, Crespo et al. 2014b). It was possible to perform species distribution modeling to predict its potential range with confidence limits. See methods for details.

Min Elevation/Depth (m): 250

Max Elevation/Depth (m): 1550

Range description: *Frontiphantes fulgurenotatus* is known throughout the laurisilva forest that occupies about 20% of the island, mainly on its steep and humid northern slopes.

#### New occurrences

#### Extent of occurrence

EOO (km2): 208-361-720

Trend: Stable

Justification for trend: The preferred habitat of the species, humid laurisilva forest, is not experiencing any decline in area and the invasive species present should not affect the spider population.

Causes ceased?: Yes

Causes understood?: Yes

Causes reversible?: Yes

Extreme fluctuations?: No

#### Area of occupancy

Trend: Stable

Justification for trend: The preferred habitat of the species, humid laurisilva forest, is not experiencing any decline in area and the invasive species present should not affect the spider population.

Causes ceased?: Yes

Causes understood?: Yes

Causes reversible?: Yes

Extreme fluctuations?: No

AOO (km2): 144-356-720

#### Locations

Number of locations: 0

Justification for number of locations: No known threats to the species.

Trend: Stable

Extreme fluctuations?: No

#### Population

Number of individuals: Unknown

Trend: Stable

Justification for trend: The preferred habitat of the species, humid laurisilva forest, is not experiencing any decline in area and the invasive species present should not affect the spider population.

Causes ceased?: Yes

Causes understood?: Yes

Causes reversible?: Yes

Extreme fluctuations?: No

Population Information (Narrative): No population size estimates exist.

#### Subpopulations

Trend: Stable

Extreme fluctuations?: No

Severe fragmentation?: No

#### Habitat

System: Terrestrial

Habitat specialist: Yes

Habitat (narrative): Humid laurisilva forest on the northern slopes of Madeira Island.

Trend in extent, area or quality?: Stable

Justification for trend: The preferred habitat of the species, humid laurisilva forest, is not experiencing any decline in area and the invasive species present should not affect the spider population.

##### Habitat

Habitat importance: Major Importance

Habitats: 1.9. Forest - Subtropical/Tropical Moist Montane

#### Habitat

Habitat importance: Major Importance

Habitats: 1.9. Forest - Subtropical/Tropical Moist Montane

#### Ecology

Size: 4 mm

Generation length (yr): 1

Dependency of single sp?: No

Ecology and traits (narrative): Sheet-web builder on the tree branches feeding mainly on small insects.

#### Threats

Justification for threats: Unknown threats.

##### Threats

Threat type: Past

Threats: 12. Other options - Other threat

#### Threats

Threat type: Past

Threats: 12. Other options - Other threat

#### Conservation

Justification for conservation actions: Most of the species range is inside the Madeira Natural Park.

##### Conservation actions

Conservation action type: In Place

Conservation actions: 1.1. Land/water protection - Site/area protection

#### Conservation actions

Conservation action type: In Place

Conservation actions: 1.1. Land/water protection - Site/area protection

#### Other

##### Use and trade

Use type: International

##### Ecosystem services

Ecosystem service type: Very important

##### Research needed

Research needed: 3.1. Monitoring - Population trends

Justification for research needed: Monitoring of population trends should be conducted to confirm species status.

#### Use and trade

Use type: International

#### Ecosystem services

Ecosystem service type: Very important

#### Research needed

Research needed: 3.1. Monitoring - Population trends

Justification for research needed: Monitoring of population trends should be conducted to confirm species status.

#### Viability analysis

### Hahnia insulana

#### Species information

Scientific name: Hahnia insulana

Species authority: Schenkel, 1938

Kingdom: Animalia

Phylum: Arthropoda

Class: Arachnida

Order: Araneae

Family: Hahniidae

Region for assessment: Global

#### Geographic range

Biogeographic realm: Palearctic

Countries: Portugal

Map of records (Google Earth): Suppl. material 17

Basis of EOO and AOO: Species Distribution Model

Basis (narrative): Multiple collection sites are recorded for the species, mostly recent and in laurisilva forest (Schenkel 1938, Denis 1962, Crespo et al. 2014b). It was possible to perform species distribution modeling to predict its potential range with confidence limits. See methods for details.

Min Elevation/Depth (m): 50

Max Elevation/Depth (m): 1700

Range description: *Hahnia insulana* is known throughout the laurisilva forest that occupies about 20% of the island, mainly on its steep and humid northern slopes.

#### New occurrences

#### Extent of occurrence

EOO (km2): 207-364-716

Trend: Stable

Justification for trend: The preferred habitat of the species, humid laurisilva forest, is not experiencing any decline in area and the invasive species present should not affect the spider populations.

Causes ceased?: Yes

Causes understood?: Yes

Causes reversible?: Yes

Extreme fluctuations?: No

#### Area of occupancy

Trend: Stable

Justification for trend: The preferred habitat of the species, humid laurisilva forest, is not experiencing any decline in area and the invasive species present should not affect the spider populations.

Causes ceased?: Yes

Causes understood?: Yes

Causes reversible?: Yes

Extreme fluctuations?: No

AOO (km2): 188-364-708

#### Locations

Number of locations: 0

Justification for number of locations: No known threats to the species.

Trend: Stable

Extreme fluctuations?: No

#### Population

Number of individuals: Unknown

Trend: Stable

Justification for trend: The preferred habitat of the species, humid laurisilva forest, is not experiencing any decline in area and the invasive species present should not affect the spider populations.

Causes ceased?: Yes

Causes understood?: Yes

Causes reversible?: Yes

Extreme fluctuations?: No

Population Information (Narrative): No population size estimates exist.

#### Subpopulations

Trend: Stable

Extreme fluctuations?: No

Severe fragmentation?: No

#### Habitat

System: Terrestrial

Habitat specialist: Yes

Habitat (narrative): Humid laurisilva forest on the northern slopes of Madeira Island.

Trend in extent, area or quality?: Stable

Justification for trend: The preferred habitat of the species, humid laurisilva forest, is not experiencing any decline in area and the invasive species present should not affect the spider populations.

##### Habitat

Habitat importance: Major Importance

Habitats: 1.9. Forest - Subtropical/Tropical Moist Montane

#### Habitat

Habitat importance: Major Importance

Habitats: 1.9. Forest - Subtropical/Tropical Moist Montane

#### Ecology

Size: 2 mm

Generation length (yr): 1

Dependency of single sp?: No

Ecology and traits (narrative): This species is a sheet-web builder among the leaf-litter and low vegetation feeding mainly on small insects.

#### Threats

Justification for threats: Unknown threats.

##### Threats

Threat type: Past

Threats: 12. Other options - Other threat

#### Threats

Threat type: Past

Threats: 12. Other options - Other threat

#### Conservation

Justification for conservation actions: Most of the species range is inside the Madeira Natural Park.

##### Conservation actions

Conservation action type: In Place

Conservation actions: 1.1. Land/water protection - Site/area protection

#### Conservation actions

Conservation action type: In Place

Conservation actions: 1.1. Land/water protection - Site/area protection

#### Other

##### Use and trade

Use type: International

##### Ecosystem services

Ecosystem service type: Very important

##### Research needed

Research needed: 3.1. Monitoring - Population trends

Justification for research needed: Monitoring of population trends should be conducted to confirm species status.

#### Use and trade

Use type: International

#### Ecosystem services

Ecosystem service type: Very important

#### Research needed

Research needed: 3.1. Monitoring - Population trends

Justification for research needed: Monitoring of population trends should be conducted to confirm species status.

#### Viability analysis

### Hogna biscoitoi

#### Species information

Scientific name: Hogna biscoitoi

Species authority: Wunderlich, 1992

Common names: Tarântula-de-BiscoitoBiscoito Wolf Spider

Kingdom: Animalia

Phylum: Arthropoda

Class: Arachnida

Order: Araneae

Family: Lycosidae

Taxonomic notes: Despite intensive searches during the last decade it was not possible to find this species recently described from undetermined locality in the island of Porto Santo (Wunderlich 1992). It probably is a junior synonym of *Hogna insularum*.

Region for assessment: Global

#### Geographic range

Biogeographic realm: Palearctic

Countries: Portugal

Map of records (Google Earth): Suppl. material 18

Basis of EOO and AOO: Unknown

Basis (narrative): The species EOO and AOO are unknown.

Range description: Only recorded from the Island of Porto Santo, from undetermined locality.

#### New occurrences

#### Extent of occurrence

EOO (km2): Unknown

Trend: Unknown

Causes ceased?: Unknown

Causes understood?: Unknown

Causes reversible?: Unknown

Extreme fluctuations?: Unknown

#### Area of occupancy

Trend: Unknown

Causes ceased?: Unknown

Causes understood?: Unknown

Causes reversible?: Unknown

Extreme fluctuations?: Unknown

AOO (km2): Unknown

#### Locations

Number of locations: Unknown

Trend: Unknown

Extreme fluctuations?: Unknown

#### Population

Number of individuals: Unknown

Trend: Unknown

Causes ceased?: Unknown

Causes understood?: Unknown

Causes reversible?: Unknown

Extreme fluctuations?: Unknown

Population Information (Narrative): No population size estimates exist.

#### Subpopulations

Trend: Unknown

Extreme fluctuations?: No

Severe fragmentation?: No

#### Habitat

System: Terrestrial

Habitat specialist: Unknown

Habitat (narrative): The species habitat is unknown.

Trend in extent, area or quality?: Unknown

##### Habitat

Habitat importance: Major Importance

Habitats: 18. Unknown

#### Habitat

Habitat importance: Major Importance

Habitats: 18. Unknown

#### Ecology

Size: 9-13 mm

Generation length (yr): 1

Dependency of single sp?: No

Ecology and traits (narrative): If valid, the species should be an active ground hunter feeding mainly on small/medium size arthropods.

#### Threats

Justification for threats: Unknown threats.

##### Threats

Threat type: Past

Threats: 12. Other options - Other threat

#### Threats

Threat type: Past

Threats: 12. Other options - Other threat

#### Conservation

##### Conservation actions

#### Conservation actions

#### Other

##### Use and trade

Use type: International

##### Ecosystem services

Ecosystem service type: Very important

##### Research needed

Research needed: 1.1. Research - Taxonomy1.2. Research - Population size, distribution & trends1.3. Research - Life history & ecology1.5. Research - Threats

Justification for research needed: Clarification of the taxonomic status is necessary. If valid, basic information would be needed on its distribution, ecology and possible threats.

#### Use and trade

Use type: International

#### Ecosystem services

Ecosystem service type: Very important

#### Research needed

Research needed: 1.1. Research - Taxonomy1.2. Research - Population size, distribution & trends1.3. Research - Life history & ecology1.5. Research - Threats

Justification for research needed: Clarification of the taxonomic status is necessary. If valid, basic information would be needed on its distribution, ecology and possible threats.

#### Viability analysis

### Hogna heeri

#### Species information

Scientific name: Hogna heeri

Species authority: (Thorell, 1875)

Common names: Tarântula-de-HeerHeer Wolf Spider

Kingdom: Animalia

Phylum: Arthropoda

Class: Arachnida

Order: Araneae

Family: Lycosidae

Region for assessment: Global

#### Geographic range

Biogeographic realm: Palearctic

Countries: Portugal

Map of records (Google Earth): Suppl. material 19

Basis of EOO and AOO: Species Distribution Model

Basis (narrative): Multiple collection sites are recorded for the species, many of them recent and in a number of different habitats (Thorell 1875, Warburton 1892, Simon 1897, Kulczyński 1899, Schenkel 1938, Denis 1962, Denis 1963, Wunderlich 1992, Crespo et al. 2013). It was possible to perform species distribution modeling to predict its potential range with confidence limits. See methods for details.

Min Elevation/Depth (m): 0

Max Elevation/Depth (m): 1755

Range description: *Hogna heeri* is known from varied and contrasting habitats, from open barren areas in Bugio (Desertas) to laurisilva forest in Madeira Island.

#### New occurrences

#### Extent of occurrence

EOO (km2): 876-1087-1439

Trend: Stable

Justification for trend: The species seems to be able to live on all kinds of habitat, even close to human settlements.

Causes ceased?: Yes

Causes understood?: Yes

Causes reversible?: Yes

Extreme fluctuations?: No

#### Area of occupancy

Trend: Stable

Justification for trend: The species seems to be able to live on all kinds of habitat, even close to human settlements.

Causes ceased?: Yes

Causes understood?: Yes

Causes reversible?: Yes

Extreme fluctuations?: No

AOO (km2): 36-408-812

#### Locations

Number of locations: 0

Justification for number of locations: No known threats to the species.

Trend: Stable

Extreme fluctuations?: No

#### Population

Number of individuals: Unknown

Trend: Stable

Justification for trend: The species seems to be able to live on all kinds of habitat, even close to human settlements.

Causes ceased?: Yes

Causes understood?: Yes

Causes reversible?: Yes

Extreme fluctuations?: No

Population Information (Narrative): No population size estimates exist.

#### Subpopulations

Trend: Stable

Extreme fluctuations?: No

Severe fragmentation?: No

#### Habitat

System: Terrestrial

Habitat specialist: No

Habitat (narrative): The species seems to be able to live on all kinds of habitat, from barren areas to laurisilva forest.

Trend in extent, area or quality?: Stable

##### Habitat

Habitat importance: Major Importance

Habitats: 1.4. Forest - Temperate1.9. Forest - Subtropical/Tropical Moist Montane3.8. Shrubland - Mediterranean-type Shrubby Vegetation4.4. Grassland - Temperate4.7. Grassland - Subtropical/High Altitude6. Rocky areas (e.g. inland cliffs, mountain peaks)16. Introduced vegetation

#### Habitat

Habitat importance: Major Importance

Habitats: 1.4. Forest - Temperate1.9. Forest - Subtropical/Tropical Moist Montane3.8. Shrubland - Mediterranean-type Shrubby Vegetation4.4. Grassland - Temperate4.7. Grassland - Subtropical/High Altitude6. Rocky areas (e.g. inland cliffs, mountain peaks)16. Introduced vegetation

#### Ecology

Size: 13-15 mm

Generation length (yr): 2

Dependency of single sp?: No

Ecology and traits (narrative): Active ground hunter feeding mainly on small/medium size arthropods.

#### Threats

Justification for threats: Unknown threats.

##### Threats

Threat type: Past

Threats: 12. Other options - Other threat

#### Threats

Threat type: Past

Threats: 12. Other options - Other threat

#### Conservation

Justification for conservation actions: Part of the species range is inside the Madeira Natural Park and in the Desertas Nature Reserve.

##### Conservation actions

Conservation action type: In Place

Conservation actions: 1. Land/water protection

#### Conservation actions

Conservation action type: In Place

Conservation actions: 1. Land/water protection

#### Other

##### Use and trade

Use type: International

##### Ecosystem services

Ecosystem service type: Very important

##### Research needed

Research needed: 1.2. Research - Population size, distribution & trends3.1. Monitoring - Population trends

Justification for research needed: The disjunct distribution of the species is currently being researched and might lead to a change in the current estimated maps. Monitoring of population trends should be conducted to confirm species status.

#### Use and trade

Use type: International

#### Ecosystem services

Ecosystem service type: Very important

#### Research needed

Research needed: 1.2. Research - Population size, distribution & trends3.1. Monitoring - Population trends

Justification for research needed: The disjunct distribution of the species is currently being researched and might lead to a change in the current estimated maps. Monitoring of population trends should be conducted to confirm species status.

#### Viability analysis

### Hogna insularum

#### Species information

Scientific name: Hogna insularum

Species authority: (Kulczynski, 1899)

Kingdom: Animalia

Phylum: Arthropoda

Class: Arachnida

Order: Araneae

Family: Lycosidae

**Figure 6.**
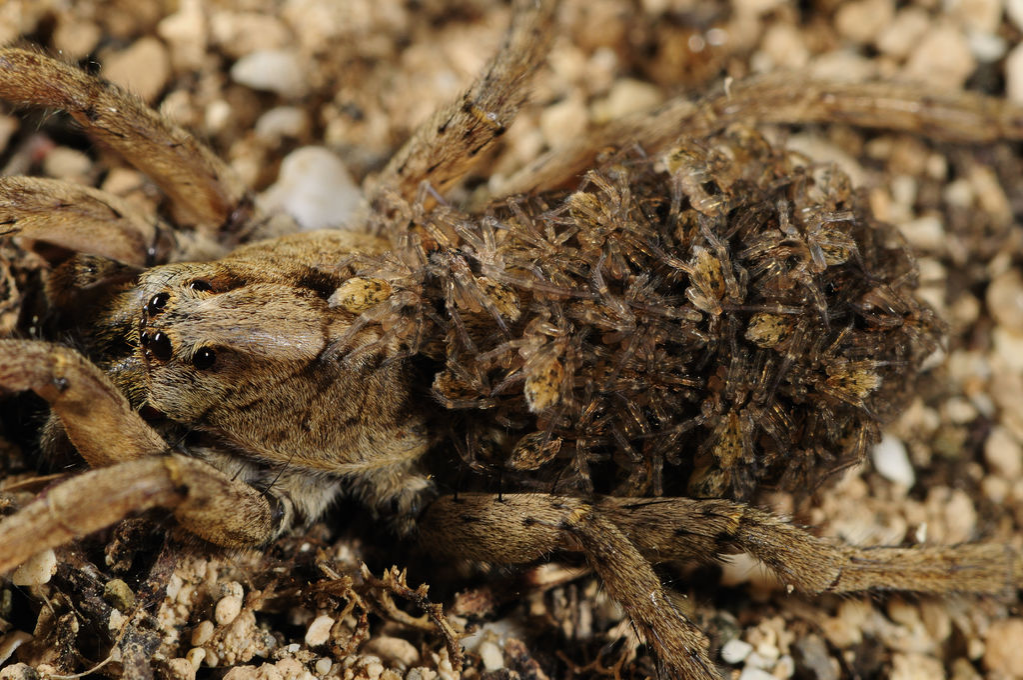
*Hogna insularum* (Kulczynski, 1899) female with spiderlings (photo by Pedro Cardoso).

Figure(s) or Photo(s): Fig. 6

Region for assessment: Global

#### Geographic range

Biogeographic realm: Palearctic

Countries: Portugal

Map of records (Google Earth): Suppl. material 20

Basis of EOO and AOO: Species Distribution Model

Basis (narrative): Multiple collection sites have been recorded for the species, mostly recent in all Madeiran islands at low altitude (Kulczyński 1899, Denis 1962, Denis 1963, Schmidt 1990, Wunderlich 1992, Crespo et al. 2013). It was possible to perform species distribution modeling to predict its potential range with confidence limits. See methods for details.

Min Elevation/Depth (m): 0

Max Elevation/Depth (m): 320

Range description: *Hogna insularum* is known in open habitats across all Madeiran islands at low altitudes. On Madeira Island it only occupies the eastern region.

#### New occurrences

#### Extent of occurrence

EOO (km2): 1333-2412-3518

Trend: Stable

Justification for trend: The preferred habitat of the species, open grassland or shrubland with frequent rock outcrops, is not experiencing any decline in area and the invasive species present should not affect the spider populations.

Causes ceased?: Yes

Causes understood?: Yes

Causes reversible?: Yes

Extreme fluctuations?: No

#### Area of occupancy

Trend: Stable

Justification for trend: The preferred habitat of the species, open grassland or scrubland with frequent rock outcrops, is not experiencing any decline in area and the invasive species present should not affect the spider populations.

Causes ceased?: Yes

Causes understood?: Yes

Causes reversible?: Yes

Extreme fluctuations?: No

AOO (km2): 80-208-644

#### Locations

Number of locations: 0

Justification for number of locations: No known threats to the species.

Trend: Stable

Extreme fluctuations?: No

#### Population

Number of individuals: Unknown

Trend: Stable

Justification for trend: The preferred habitat of the species, open grassland or shrubland with frequent rock outcrops, is not experiencing any decline in area and the invasive species present should not affect the spider populations.

Causes ceased?: Yes

Causes understood?: Yes

Causes reversible?: Yes

Extreme fluctuations?: No

Population Information (Narrative): No population size estimates exist.

#### Subpopulations

Trend: Stable

Extreme fluctuations?: No

Severe fragmentation?: No

#### Habitat

System: Terrestrial

Habitat specialist: No

Habitat (narrative): Open grassland or shrubland with frequent rock outcrops, very common in the eastern part of the archipelago at low altitudes.

Trend in extent, area or quality?: Stable

Justification for trend: The preferred habitat of the species is not experiencing any decline in area and the invasive species present should not affect the spider populations.

##### Habitat

Habitat importance: Major Importance

Habitats: 3.8. Shrubland - Mediterranean-type Shrubby Vegetation4.5. Grassland - Subtropical/Tropical Dry6. Rocky areas (e.g. inland cliffs, mountain peaks)

#### Habitat

Habitat importance: Major Importance

Habitats: 3.8. Shrubland - Mediterranean-type Shrubby Vegetation4.5. Grassland - Subtropical/Tropical Dry6. Rocky areas (e.g. inland cliffs, mountain peaks)

#### Ecology

Size: 11-20 mm

Generation length (yr): 1

Dependency of single sp?: No

Ecology and traits (narrative): Active ground hunter feeding mainly on small/medium size arthropods.

#### Threats

Justification for threats: Unknown threats.

##### Threats

Threat type: Past

Threats: 12. Other options - Other threat

#### Threats

Threat type: Past

Threats: 12. Other options - Other threat

#### Conservation

Justification for conservation actions: Part of the species range is inside several protected areas including the Ponta de São Lourenco Special Protection Area and Desertas Nature Reserve.

##### Conservation actions

Conservation action type: In Place

Conservation actions: 1.1. Land/water protection - Site/area protection

#### Conservation actions

Conservation action type: In Place

Conservation actions: 1.1. Land/water protection - Site/area protection

#### Other

##### Use and trade

Use type: International

##### Ecosystem services

Ecosystem service type: Very important

##### Research needed

Research needed: 3.1. Monitoring - Population trends

Justification for research needed: Monitoring of population trends should be conducted to confirm species status.

#### Use and trade

Use type: International

#### Ecosystem services

Ecosystem service type: Very important

#### Research needed

Research needed: 3.1. Monitoring - Population trends

Justification for research needed: Monitoring of population trends should be conducted to confirm species status.

#### Viability analysis

### Hogna maderiana

#### Species information

Scientific name: Hogna maderiana

Species authority: (Walckenaer, 1837)

Common names: Tarântula-da-MadeiraMadeira Wolf Spider

Kingdom: Animalia

Phylum: Arthropoda

Class: Arachnida

Order: Araneae

Family: Lycosidae

Region for assessment: Global

#### Geographic range

Biogeographic realm: Palearctic

Countries: Portugal

Map of records (Google Earth): Suppl. material 21

Basis of EOO and AOO: Species Distribution Model

Basis (narrative): Multiple collection sites are recorded for the species, usually in open areas but at all altitudes, from coastal areas to the plateau of Paúl da Serra (Walckenaer 1837, Johnson 1863, Thorell 1875, Warburton 1892, Simon 1897, Kulczyński 1899, Schenkel 1938, Roewer 1960, Denis 1962, Denis 1963, Wunderlich 1992, Wunderlich 1995). It was possible to perform species distribution modeling to predict its potential range with confidence limits. See methods for details.

Min Elevation/Depth (m): 0

Max Elevation/Depth (m): 1800

Range description: *Hogna maderiana* is known throughout the island of Madeira in open habitats.

#### New occurrences

#### Extent of occurrence

EOO (km2): 304-368-908

Trend: Stable

Justification for trend: The species seems to be able to live on several habitat types, even close to human settlements.

Causes ceased?: Yes

Causes understood?: Yes

Causes reversible?: Yes

Extreme fluctuations?: No

#### Area of occupancy

Trend: Stable

Justification for trend: The species seems to be able to live on several habitat types, even close to human settlements.

Causes ceased?: Yes

Causes understood?: Yes

Causes reversible?: Yes

Extreme fluctuations?: No

AOO (km2): 28-368-908

#### Locations

Number of locations: 0

Justification for number of locations: No known threats to the species.

Trend: Stable

Extreme fluctuations?: No

#### Population

Number of individuals: Unknown

Trend: Stable

Justification for trend: The species seems to be able to live on several habitat types, even close to human settlements.

Causes ceased?: Yes

Causes understood?: Yes

Causes reversible?: Yes

Extreme fluctuations?: No

Population Information (Narrative): No population size estimates exist.

#### Subpopulations

Trend: Stable

Extreme fluctuations?: No

Severe fragmentation?: No

#### Habitat

System: Terrestrial

Habitat specialist: No

Habitat (narrative): The species seems to be able to live on several open habitat types, including coastal areas and high-altitude plateaus dominated by grasses.

Trend in extent, area or quality?: Stable

##### Habitat

Habitat importance: Major Importance

Habitats: 3.8. Shrubland - Mediterranean-type Shrubby Vegetation4.7. Grassland - Subtropical/High Altitude6. Rocky areas (e.g. inland cliffs, mountain peaks)13.1. Marine Coastal/Supratidal - Sea Cliffs and Rocky Offshore Islands

#### Habitat

Habitat importance: Major Importance

Habitats: 3.8. Shrubland - Mediterranean-type Shrubby Vegetation4.7. Grassland - Subtropical/High Altitude6. Rocky areas (e.g. inland cliffs, mountain peaks)13.1. Marine Coastal/Supratidal - Sea Cliffs and Rocky Offshore Islands

#### Ecology

Size: 25 mm

Generation length (yr): 2

Dependency of single sp?: No

Ecology and traits (narrative): Active ground hunter feeding mainly on small/medium size arthropods.

#### Threats

Justification for threats: Unknown threats.

##### Threats

Threat type: Past

Threats: 12. Other options - Other threat

#### Threats

Threat type: Past

Threats: 12. Other options - Other threat

#### Conservation

##### Conservation actions

#### Conservation actions

#### Other

##### Use and trade

Use type: International

##### Ecosystem services

Ecosystem service type: Very important

##### Research needed

Research needed: 1.2. Research - Population size, distribution & trends3.1. Monitoring - Population trends

Justification for research needed: The disjunct distribution of the species is currently being researched and might lead to a change in the current estimated maps. Monitoring of population trends should be conducted to confirm species status.

#### Use and trade

Use type: International

#### Ecosystem services

Ecosystem service type: Very important

#### Research needed

Research needed: 1.2. Research - Population size, distribution & trends3.1. Monitoring - Population trends

Justification for research needed: The disjunct distribution of the species is currently being researched and might lead to a change in the current estimated maps. Monitoring of population trends should be conducted to confirm species status.

#### Viability analysis

### Hogna nonannulata

#### Species information

Scientific name: Hogna nonannulata

Species authority: Wunderlich, 1995

Kingdom: Animalia

Phylum: Arthropoda

Class: Arachnida

Order: Araneae

Family: Lycosidae

Region for assessment: Global

#### Geographic range

Biogeographic realm: Palearctic

Countries: Portugal

Map of records (Google Earth): Suppl. material 22

Basis of EOO and AOO: Unknown

Basis (narrative): The species EOO and AOO are unknown.

Range description: The true distribution of *Hogna nonannulata* is unknown (Roewer 1960, Wunderlich 1995), although it might be present exclusively in laurisilva forest.

#### New occurrences

#### Extent of occurrence

EOO (km2): Unknown

Trend: Unknown

Causes ceased?: Unknown

Causes understood?: Unknown

Causes reversible?: Unknown

Extreme fluctuations?: Unknown

#### Area of occupancy

Trend: Unknown

Causes ceased?: Unknown

Causes understood?: Unknown

Causes reversible?: Unknown

Extreme fluctuations?: Unknown

AOO (km2): Unknown

#### Locations

Number of locations: Unknown

Trend: Unknown

Extreme fluctuations?: Unknown

#### Population

Number of individuals: Unknown

Trend: Unknown

Causes ceased?: Unknown

Causes understood?: Unknown

Causes reversible?: Unknown

Extreme fluctuations?: Unknown

Population Information (Narrative): No population size estimates exist.

#### Subpopulations

Trend: Unknown

Extreme fluctuations?: Unknown

Severe fragmentation?: Unknown

#### Habitat

System: Terrestrial

Habitat specialist: Yes

Habitat (narrative): The species was found from few sites in humid laurisilva forest (Ribeiro Frio) on the northern slopes of Madeira Island.

Trend in extent, area or quality?: Unknown

##### Habitat

Habitat importance: Major Importance

Habitats: 1.9. Forest - Subtropical/Tropical Moist Montane

#### Habitat

Habitat importance: Major Importance

Habitats: 1.9. Forest - Subtropical/Tropical Moist Montane

#### Ecology

Size: 15-17 mm

Generation length (yr): 2

Dependency of single sp?: No

Ecology and traits (narrative): Active ground hunter feeding mainly on small/medium size arthropods.

#### Threats

Justification for threats: Unknown threats.

##### Threats

Threat type: Past

Threats: 12. Other options - Other threat

#### Threats

Threat type: Past

Threats: 12. Other options - Other threat

#### Conservation

Justification for conservation actions: The known locality is inside the Madeira Natural Park.

##### Conservation actions

Conservation action type: In Place

Conservation actions: 1.1. Land/water protection - Site/area protection

#### Conservation actions

Conservation action type: In Place

Conservation actions: 1.1. Land/water protection - Site/area protection

#### Other

##### Use and trade

Use type: International

##### Ecosystem services

Ecosystem service type: Very important

##### Research needed

Research needed: 1.2. Research - Population size, distribution & trends1.3. Research - Life history & ecology1.5. Research - Threats

Justification for research needed: Basic information is needed on its distribution, ecology and possible threats.

#### Use and trade

Use type: International

#### Ecosystem services

Ecosystem service type: Very important

#### Research needed

Research needed: 1.2. Research - Population size, distribution & trends1.3. Research - Life history & ecology1.5. Research - Threats

Justification for research needed: Basic information is needed on its distribution, ecology and possible threats.

#### Viability analysis

### Hogna schmitzi

#### Species information

Scientific name: Hogna schmitzi

Species authority: Wunderlich, 1992

Common names: Tarântula-de-Porto-Santo (Portuguese)Porto Santo Wolf Spider (English)

Kingdom: Animalia

Phylum: Arthropoda

Class: Arachnida

Order: Araneae

Family: Lycosidae

**Figure 7.**
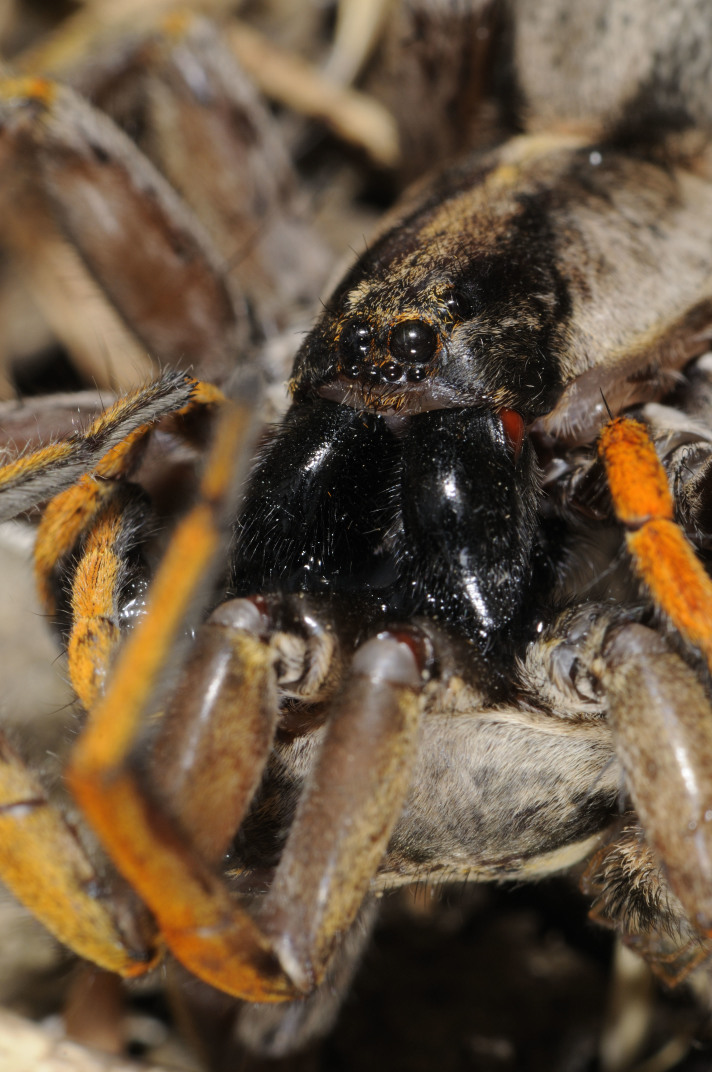
*Hogna schmitzi* Wunderlich, 1992 (photo by Pedro Cardoso).

Figure(s) or Photo(s): Fig. 7

Region for assessment: Global

#### Geographic range

Biogeographic realm: Palearctic

Countries: Portugal

Map of records (Google Earth): Suppl. material 23

Basis of EOO and AOO: Species Distribution Model

Basis (narrative): Multiple collection sites are recorded for the species (Blackwall 1857, Johnson 1863, Schmitz 1895, Kulczyński 1899, Cockerell 1924, Denis 1962, Schmidt 1990, Wunderlich 1992). It was possible to perform species distribution modeling to predict its potential range with confidence limits. See methods for details.

Min Elevation/Depth (m): 0

Max Elevation/Depth (m): 270

Range description: Across the entire island of Porto Santo and its small islets. Habitats include areas close to human settlements but exclude densely forested areas.

#### New occurrences

#### Extent of occurrence

EOO (km2): 32-64-68

Trend: Stable

Justification for trend: The species seems to be able to live on several habitat types, even close to human settlements.

Causes ceased?: Yes

Causes understood?: Yes

Causes reversible?: Yes

Extreme fluctuations?: No

#### Area of occupancy

Trend: Stable

Justification for trend: The species seems to be able to live on several habitat types, even close to human settlements.

Causes ceased?: Yes

Causes understood?: Yes

Causes reversible?: Yes

Extreme fluctuations?: No

AOO (km2): 32-64-68

#### Locations

Number of locations: 0

Justification for number of locations: No current threats to the species.

Trend: Stable

Extreme fluctuations?: No

#### Population

Number of individuals: Unknown

Trend: Stable

Justification for trend: The species seems to be able to live on several habitat types, even close to human settlements.

Causes ceased?: Yes

Causes understood?: Yes

Causes reversible?: Yes

Extreme fluctuations?: No

Population Information (Narrative): No population size estimates exist.

#### Subpopulations

Trend: Stable

Extreme fluctuations?: No

Severe fragmentation?: No

#### Habitat

System: Terrestrial

Habitat specialist: No

Habitat (narrative): The species seems to be able to live on several habitat types, even close to human settlements. It only avoids densely forested areas.

Trend in extent, area or quality?: Stable

##### Habitat

Habitat importance: Major Importance

Habitats: 3.8. Shrubland - Mediterranean-type Shrubby Vegetation4.5. Grassland - Subtropical/Tropical Dry6. Rocky areas (e.g. inland cliffs, mountain peaks)

#### Habitat

Habitat importance: Major Importance

Habitats: 3.8. Shrubland - Mediterranean-type Shrubby Vegetation4.5. Grassland - Subtropical/Tropical Dry6. Rocky areas (e.g. inland cliffs, mountain peaks)

#### Ecology

Size: 20-30 mm

Generation length (yr): 2

Dependency of single sp?: No

Ecology and traits (narrative): Active ground hunter feeding mainly on medium/large size arthropods.

#### Threats

Justification for threats: Unknown threats.

##### Threats

Threat type: Past

Threats: 12. Other options - Other threat

#### Threats

Threat type: Past

Threats: 12. Other options - Other threat

#### Conservation

Justification for conservation actions: A small part of the species range is inside the Porto Santo Network of Marine Protected Areas (which include islets).

##### Conservation actions

Conservation action type: In Place

Conservation actions: 1.1. Land/water protection - Site/area protection

#### Conservation actions

Conservation action type: In Place

Conservation actions: 1.1. Land/water protection - Site/area protection

#### Other

##### Use and trade

Use type: International

##### Ecosystem services

Ecosystem service type: Very important

##### Research needed

Research needed: 3.1. Monitoring - Population trends

Justification for research needed: Monitoring of population trends should be conducted to confirm species status.

#### Use and trade

Use type: International

#### Ecosystem services

Ecosystem service type: Very important

#### Research needed

Research needed: 3.1. Monitoring - Population trends

Justification for research needed: Monitoring of population trends should be conducted to confirm species status.

#### Viability analysis

### Lathys affinis

#### Species information

Scientific name: Lathys affinis

Species authority: (Blackwall, 1862)

Kingdom: Animalia

Phylum: Arthropoda

Class: Arachnida

Order: Araneae

Family: Dictynidae

Region for assessment: Global

#### Geographic range

Biogeographic realm: Palearctic

Countries: Portugal

Map of records (Google Earth): Suppl. material 24

Basis of EOO and AOO: Species Distribution Model

Basis (narrative): Multiple collection sites are recorded for the species, mostly recent and in all habitat types (Blackwall 1862, Kulczyński 1899, Schenkel 1938, Denis 1962, Wunderlich 1992, Crespo et al. 2013, Crespo et al. 2014b). It was possible to perform species distribution modeling to predict its potential range with confidence limits. See methods for details.

Min Elevation/Depth (m): 0

Max Elevation/Depth (m): 1460

Range description: *Lathys affinis* is known on all islands across the Madeira archipelago and on all habitat types, from laurisilva forest to open arid areas.

#### New occurrences

#### Extent of occurrence

EOO (km2): 744-3373-3699

Trend: Stable

Justification for trend: The species seems to be able to live on several habitat types, even close to human settlements.

Causes ceased?: Yes

Causes understood?: Yes

Causes reversible?: Yes

Extreme fluctuations?: No

#### Area of occupancy

Trend: Stable

Justification for trend: The species seems to be able to live on several habitat types, even close to human settlements.

Causes ceased?: Yes

Causes understood?: Yes

Causes reversible?: Yes

Extreme fluctuations?: No

AOO (km2): 40-544-948

#### Locations

Number of locations: 0

Justification for number of locations: No known threats to the species.

Trend: Stable

Extreme fluctuations?: No

#### Population

Number of individuals: Unknown

Trend: Stable

Justification for trend: The species seems to be able to live on several habitat types, even close to human settlements.

Causes ceased?: Yes

Causes understood?: Yes

Causes reversible?: Yes

Extreme fluctuations?: No

Population Information (Narrative): No population size estimates exist.

#### Subpopulations

Trend: Stable

Extreme fluctuations?: No

Severe fragmentation?: No

#### Habitat

System: Terrestrial

Habitat specialist: No

Habitat (narrative): The species seems to be able to live on several habitat types, even close to human settlements.

Trend in extent, area or quality?: Stable

##### Habitat

Habitat importance: Major Importance

Habitats: 1.4. Forest - Temperate1.9. Forest - Subtropical/Tropical Moist Montane3.8. Shrubland - Mediterranean-type Shrubby Vegetation4.5. Grassland - Subtropical/Tropical Dry4.7. Grassland - Subtropical/High Altitude6. Rocky areas (e.g. inland cliffs, mountain peaks)16. Introduced vegetation

#### Habitat

Habitat importance: Major Importance

Habitats: 1.4. Forest - Temperate1.9. Forest - Subtropical/Tropical Moist Montane3.8. Shrubland - Mediterranean-type Shrubby Vegetation4.5. Grassland - Subtropical/Tropical Dry4.7. Grassland - Subtropical/High Altitude6. Rocky areas (e.g. inland cliffs, mountain peaks)16. Introduced vegetation

#### Ecology

Size: 2 mm

Generation length (yr): 1

Dependency of single sp?: No

Ecology and traits (narrative): Small cribellate web builder on vegetation or ground level feeding mainly on small insects.

#### Threats

Justification for threats: Unknown threats.

##### Threats

Threat type: Past

Threats: 12. Other options - Other threat

#### Threats

Threat type: Past

Threats: 12. Other options - Other threat

#### Conservation

Justification for conservation actions: Much of the species range is inside the Madeira Natural Park and in several protected areas across the archipelago.

##### Conservation actions

Conservation action type: In Place

Conservation actions: 1.1. Land/water protection - Site/area protection

#### Conservation actions

Conservation action type: In Place

Conservation actions: 1.1. Land/water protection - Site/area protection

#### Other

##### Use and trade

Use type: International

##### Ecosystem services

Ecosystem service type: Very important

##### Research needed

Research needed: 3.1. Monitoring - Population trends

Justification for research needed: Monitoring of population trends should be conducted to confirm species status.

#### Use and trade

Use type: International

#### Ecosystem services

Ecosystem service type: Very important

#### Research needed

Research needed: 3.1. Monitoring - Population trends

Justification for research needed: Monitoring of population trends should be conducted to confirm species status.

#### Viability analysis

### Lepthyphantes impudicus

#### Species information

Scientific name: Lepthyphantes impudicus

Species authority: Kulczynski, 1909

Kingdom: Animalia

Phylum: Arthropoda

Class: Arachnida

Order: Araneae

Family: Linyphiidae

Region for assessment: Global

#### Geographic range

Biogeographic realm: Palearctic

Countries: Portugal

Map of records (Google Earth): Suppl. material 25

Basis of EOO and AOO: Species Distribution Model

Basis (narrative): Multiple collection sites are recorded for the species, mostly recent and in laurisilva forest (Kulczyński 1909, Denis 1962, Crespo et al. 2014b). It was possible to perform species distribution modeling to predict its potential range with confidence limits. See methods for details.

Min Elevation/Depth (m): 200

Max Elevation/Depth (m): 1850

Range description: *Lepthyphantes impudicus* is known throughout the laurisilva forest that occupies about 20% of the island, mainly on its steep and humid northern slopes.

#### New occurrences

#### Extent of occurrence

EOO (km2): 153-400-736

Trend: Stable

Justification for trend: The preferred habitat of the species, humid laurisilva forest, is not experiencing a decline in area and the invasive species present should not affect the spider populations.

Causes ceased?: Yes

Causes understood?: Yes

Causes reversible?: Yes

Extreme fluctuations?: No

#### Area of occupancy

Trend: Stable

Justification for trend: The preferred habitat of the species, humid laurisilva forest, is not experiencing any decline in area and the invasive species present should not affect the spider populations.

Causes ceased?: Yes

Causes understood?: Yes

Causes reversible?: Yes

Extreme fluctuations?: No

AOO (km2): 152-400-736

#### Locations

Number of locations: 0

Justification for number of locations: No known threats to the species.

Trend: Stable

Extreme fluctuations?: No

#### Population

Number of individuals: Unknown

Trend: Stable

Justification for trend: The preferred habitat of the species, humid laurisilva forest, is not experiencing any decline in area and the invasive species present should not affect the spider populations.

Causes ceased?: Yes

Causes understood?: Yes

Causes reversible?: Yes

Extreme fluctuations?: No

Population Information (Narrative): No population size estimates exist.

#### Subpopulations

Trend: Stable

Extreme fluctuations?: No

Severe fragmentation?: No

#### Habitat

System: Terrestrial

Habitat specialist: Yes

Habitat (narrative): Humid laurisilva forest on the northern slopes of Madeira Island.

Trend in extent, area or quality?: Stable

Justification for trend: The preferred habitat of the species, humid laurisilva forest, is not experiencing any decline in area and the invasive species present should not affect the spider populations.

##### Habitat

Habitat importance: Major Importance

Habitats: 1.9. Forest - Subtropical/Tropical Moist Montane

#### Habitat

Habitat importance: Major Importance

Habitats: 1.9. Forest - Subtropical/Tropical Moist Montane

#### Ecology

Size: 3 mm

Generation length (yr): 1

Dependency of single sp?: No

Ecology and traits (narrative): This species is a sheet-web builder on the tree branches feeding mainly on small insects.

#### Threats

Justification for threats: Unknown threats.

##### Threats

Threat type: Past

Threats: 12. Other options - Other threat

#### Threats

Threat type: Past

Threats: 12. Other options - Other threat

#### Conservation

Justification for conservation actions: Most of the species range is inside the Madeira Natural Park.

##### Conservation actions

Conservation action type: In Place

Conservation actions: 1.1. Land/water protection - Site/area protection

#### Conservation actions

Conservation action type: In Place

Conservation actions: 1.1. Land/water protection - Site/area protection

#### Other

##### Use and trade

Use type: International

##### Ecosystem services

Ecosystem service type: Very important

##### Research needed

Research needed: 3.1. Monitoring - Population trends

Justification for research needed: Monitoring of population trends should be conducted to confirm species status.

#### Use and trade

Use type: International

#### Ecosystem services

Ecosystem service type: Very important

#### Research needed

Research needed: 3.1. Monitoring - Population trends

Justification for research needed: Monitoring of population trends should be conducted to confirm species status.

#### Viability analysis

### Lepthyphantes lundbladi

#### Species information

Scientific name: Lepthyphantes lundbladi

Species authority: Schenkel, 1938

Kingdom: Animalia

Phylum: Arthropoda

Class: Arachnida

Order: Araneae

Family: Linyphiidae

Region for assessment: Global

#### Geographic range

Biogeographic realm: Palearctic

Countries: Portugal

Map of records (Google Earth): Suppl. material 26

Basis of EOO and AOO: Species Distribution Model

Basis (narrative): Multiple collection sites are recorded for the species in laurisilva forest (Schenkel 1938, Wunderlich 1987). It was possible to perform species distribution modeling to predict its potential range with confidence limits. See methods for details.

Min Elevation/Depth (m): 750

Max Elevation/Depth (m): 1850

Range description: *Lepthyphantes lundbladi* is predicted to be present throughout the laurisilva forest that occupies about 20% of the island, mainly on its steep and humid northern slopes.

#### New occurrences

#### Extent of occurrence

EOO (km2): 40-288-600

Trend: Stable

Justification for trend: The preferred habitat of the species, humid laurisilva forest, is not experiencing any decline in area and the invasive species present should not affect the spider populations.

Causes ceased?: Yes

Causes understood?: Yes

Causes reversible?: Yes

Extreme fluctuations?: No

#### Area of occupancy

Trend: Stable

Justification for trend: The preferred habitat of the species, humid laurisilva forest, is not experiencing any decline in area and the invasive species present should not affect the spider populations.

Causes ceased?: Yes

Causes understood?: Yes

Causes reversible?: Yes

Extreme fluctuations?: No

AOO (km2): 40-288-600

#### Locations

Number of locations: 0

Justification for number of locations: No known threats to the species.

Trend: Stable

Extreme fluctuations?: No

#### Population

Number of individuals: Unknown

Trend: Stable

Justification for trend: The preferred habitat of the species, humid laurisilva forest, is not experiencing any decline in area and the invasive species present should not affect the spider populations.

Causes ceased?: Yes

Causes understood?: Yes

Causes reversible?: Yes

Extreme fluctuations?: No

Population Information (Narrative): No population size estimates exist.

#### Subpopulations

Trend: Stable

Extreme fluctuations?: No

Severe fragmentation?: No

#### Habitat

System: Terrestrial

Habitat specialist: Yes

Habitat (narrative): Humid laurisilva forest on the northern slopes of Madeira Island.

Trend in extent, area or quality?: Stable

##### Habitat

Habitat importance: Major Importance

Habitats: 1.9. Forest - Subtropical/Tropical Moist Montane

#### Habitat

Habitat importance: Major Importance

Habitats: 1.9. Forest - Subtropical/Tropical Moist Montane

#### Ecology

Size: 3 mm

Generation length (yr): 1

Dependency of single sp?: No

Ecology and traits (narrative): This species is a sheet-web builder close to the soil, being first described from the entrance of a lava tube (Gruta da Ribeira do Inferno).

#### Threats

Justification for threats: Unknown threats.

##### Threats

Threat type: Past

Threats: 12. Other options - Other threat

#### Threats

Threat type: Past

Threats: 12. Other options - Other threat

#### Conservation

Justification for conservation actions: Most of the species range is inside the Madeira Natural Park.

##### Conservation actions

Conservation action type: In Place

Conservation actions: 1.1. Land/water protection - Site/area protection

#### Conservation actions

Conservation action type: In Place

Conservation actions: 1.1. Land/water protection - Site/area protection

#### Other

##### Use and trade

Use type: International

##### Ecosystem services

Ecosystem service type: Very important

##### Research needed

Research needed: 3.1. Monitoring - Population trends

Justification for research needed: Monitoring of population trends should be conducted to confirm species status.

#### Use and trade

Use type: International

#### Ecosystem services

Ecosystem service type: Very important

#### Research needed

Research needed: 3.1. Monitoring - Population trends

Justification for research needed: Monitoring of population trends should be conducted to confirm species status.

#### Viability analysis

### Lepthyphantes mauli

#### Species information

Scientific name: Lepthyphantes mauli

Species authority: Wunderlich, 1992

Kingdom: Animalia

Phylum: Arthropoda

Class: Arachnida

Order: Araneae

Family: Linyphiidae

Region for assessment: Global

#### Geographic range

Biogeographic realm: Palearctic

Countries: Portugal

Map of records (Google Earth): Suppl. material 27

Basis of EOO and AOO: Species Distribution Model

Basis (narrative): Multiple collection sites are recorded for the species, mostly recent and in laurisilva forest (Wunderlich 1992, Crespo et al. 2014b). It was possible to perform species distribution modeling to predict its potential range with confidence limits. See methods for details.

Min Elevation/Depth (m): 50

Max Elevation/Depth (m): 1850

Range description: *Lepthyphantes mauli* is known from different sites in laurisilva forest that occupies about 20% of the island, mainly on its steep and humid northern slopes.

#### New occurrences

#### Extent of occurrence

EOO (km2): 200-316-524

Trend: Stable

Justification for trend: The preferred habitat of the species, humid laurisilva forest, is not experiencing any decline in area and the invasive species present should not affect the spider populations.

Causes ceased?: Yes

Causes understood?: Yes

Causes reversible?: Yes

Extreme fluctuations?: No

#### Area of occupancy

Trend: Stable

Justification for trend: The preferred habitat of the species, humid laurisilva forest, is not experiencing any decline in area and the invasive species present should not affect the spider populations.

Causes ceased?: Yes

Causes understood?: Yes

Causes reversible?: Yes

Extreme fluctuations?: No

AOO (km2): 200-316-524

#### Locations

Number of locations: 0

Justification for number of locations: No known threats to the species.

Trend: Stable

Extreme fluctuations?: No

#### Population

Number of individuals: Unknown

Trend: Stable

Justification for trend: The preferred habitat of the species, humid laurisilva forest, is not experiencing any decline in area and the invasive species present should not affect the spider populations.

Causes ceased?: Yes

Causes understood?: Yes

Causes reversible?: Yes

Extreme fluctuations?: No

Population Information (Narrative): No population size estimates exist.

#### Subpopulations

Trend: Stable

Extreme fluctuations?: No

Severe fragmentation?: No

#### Habitat

System: Terrestrial

Habitat specialist: Yes

Habitat (narrative): Humid laurisilva forest on the northern slopes of Madeira Island.

Trend in extent, area or quality?: Stable

Justification for trend: The preferred habitat of the species, humid laurisilva forest, is not experiencing any decline in area and the invasive species present should not affect the spider populations.

##### Habitat

Habitat importance: Major Importance

Habitats: 1.9. Forest - Subtropical/Tropical Moist Montane

#### Habitat

Habitat importance: Major Importance

Habitats: 1.9. Forest - Subtropical/Tropical Moist Montane

#### Ecology

Size: 3 mm

Generation length (yr): 1

Dependency of single sp?: No

Ecology and traits (narrative): This species is a sheet-web builder close to the soil, being first described from the entrance of a lava tube (Grutas de São Vicente).

#### Threats

Justification for threats: Unknown threats.

##### Threats

Threat type: Past

Threats: 12. Other options - Other threat

#### Threats

Threat type: Past

Threats: 12. Other options - Other threat

#### Conservation

##### Conservation actions

#### Conservation actions

#### Other

##### Use and trade

##### Ecosystem services

#### Use and trade

#### Ecosystem services

#### Viability analysis

### Macaroeris desertensis

#### Species information

Scientific name: Macaroeris desertensis

Species authority: Wunderlich, 1992

Kingdom: Animalia

Phylum: Arthropoda

Class: Arachnida

Order: Araneae

Family: Salticidae

Region for assessment: Global

#### Geographic range

Biogeographic realm: Palearctic

Countries: Portugal

Map of records (Google Earth): Suppl. material 28

Basis of EOO and AOO: Species Distribution Model

Basis (narrative): Multiple collection sites have been recorded for the species in both Porto Santo and Desertas (Wunderlich 1992, Crespo et al. 2013). It was possible to perform species distribution modeling to predict its potential range with confidence limits. See methods for details.

Min Elevation/Depth (m): 0

Max Elevation/Depth (m): 270

Range description: *Macaroeris desertensis* is known throughout the islands and islets of Porto Santo and Desertas, mainly on open grassland, scrubland and rocky areas.

#### New occurrences

#### Extent of occurrence

EOO (km2): 529-599-599

Trend: Stable

Justification for trend: The species seems to be common in open areas of several islands and islets.

Causes ceased?: Yes

Causes understood?: Yes

Causes reversible?: Yes

Extreme fluctuations?: No

#### Area of occupancy

Trend: Stable

Justification for trend: The species seems to be common in open areas of several islands and islets.

Causes ceased?: Yes

Causes understood?: Yes

Causes reversible?: Yes

Extreme fluctuations?: No

AOO (km2): 36-104-104

#### Locations

Number of locations: 0

Justification for number of locations: No known threats to the species.

Trend: Stable

Extreme fluctuations?: No

#### Population

Number of individuals: Unknown

Trend: Stable

Justification for trend: The species seems to be common in open areas of several islands and islets.

Causes ceased?: Yes

Causes understood?: Yes

Causes reversible?: Yes

Extreme fluctuations?: No

Population Information (Narrative): No population size estimates exist.

#### Subpopulations

Trend: Stable

Extreme fluctuations?: No

Severe fragmentation?: No

#### Habitat

System: Terrestrial

Habitat specialist: No

Habitat (narrative): *Macaroeris desertensis* is known throughout the islands and islets of Porto Santo and Desertas, mainly on open grassland, scrubland and rocky areas.

Trend in extent, area or quality?: Stable

Justification for trend: The species seems to be common in open areas of several islands and islets.

##### Habitat

Habitat importance: Major Importance

Habitats: 3.8. Shrubland - Mediterranean-type Shrubby Vegetation4.5. Grassland - Subtropical/Tropical Dry6. Rocky areas (e.g. inland cliffs, mountain peaks)

#### Habitat

Habitat importance: Major Importance

Habitats: 3.8. Shrubland - Mediterranean-type Shrubby Vegetation4.5. Grassland - Subtropical/Tropical Dry6. Rocky areas (e.g. inland cliffs, mountain peaks)

#### Ecology

Size: 4-5 mm

Generation length (yr): 1

Dependency of single sp?: No

Ecology and traits (narrative): Active hunter on low vegetation feeding mainly on small/medium size arthropods.

#### Threats

Justification for threats: Unknown threats.

##### Threats

Threat type: Past

Threats: 12. Other options - Other threat

#### Threats

Threat type: Past

Threats: 12. Other options - Other threat

#### Conservation

Justification for conservation actions: Part of the species range lies inside the Desertas Nature Reserve and in the Porto Santo Network of Marine Protected Areas (which includes islets).

##### Conservation actions

Conservation action type: In Place

Conservation actions: 1.1. Land/water protection - Site/area protection

#### Conservation actions

Conservation action type: In Place

Conservation actions: 1.1. Land/water protection - Site/area protection

#### Other

##### Use and trade

Use type: International

##### Ecosystem services

Ecosystem service type: Very important

##### Research needed

Research needed: 3.1. Monitoring - Population trends

Justification for research needed: Monitoring of population trends should be conducted to confirm species status.

#### Use and trade

Use type: International

#### Ecosystem services

Ecosystem service type: Very important

#### Research needed

Research needed: 3.1. Monitoring - Population trends

Justification for research needed: Monitoring of population trends should be conducted to confirm species status.

#### Viability analysis

### Macarophaeus cultior

#### Species information

Scientific name: Macarophaeus cultior

Species authority: (Kulczynski, 1899)

Kingdom: Animalia

Phylum: Arthropoda

Class: Arachnida

Order: Araneae

Family: Gnaphosidae

Region for assessment: Global

#### Geographic range

Biogeographic realm: Palearctic

Countries: Portugal

Map of records (Google Earth): Suppl. material 29

Basis of EOO and AOO: Species Distribution Model

Basis (narrative): Multiple collection sites are recorded for the species, mostly recent and in laurisilva forest (Kulczyński 1899, Denis 1962, Crespo et al. 2014b). It was possible to perform species distribution modeling to predict its potential range with confidence limits. See methods for details.

Min Elevation/Depth (m): 100

Max Elevation/Depth (m): 1850

Range description: *Macarophaeus cultior* is known throughout the laurisilva forest that occupies about 20% of the island, mainly on its steep and humid northern slopes.

#### New occurrences

#### Extent of occurrence

EOO (km2): 153-372-688

Trend: Stable

Justification for trend: The preferred habitat of the species, humid laurisilva forest, is not experiencing any decline in area and the invasive species present should not affect the spider populations.

Causes ceased?: Yes

Causes understood?: Yes

Causes reversible?: Yes

Extreme fluctuations?: No

#### Area of occupancy

Trend: Stable

Justification for trend: The preferred habitat of the species, humid laurisilva forest, is not experiencing any decline in area and the invasive species present should not affect the spider populations.

Causes ceased?: Yes

Causes understood?: Yes

Causes reversible?: Yes

Extreme fluctuations?: No

AOO (km2): 136-372-688

#### Locations

Number of locations: 0

Justification for number of locations: No known threats to the species.

Trend: Stable

Extreme fluctuations?: No

#### Population

Number of individuals: Unknown

Trend: Stable

Justification for trend: The preferred habitat of the species, humid laurisilva forest, is not experiencing any decline in area and the invasive species present should not affect the spider populations.

Causes ceased?: Yes

Causes understood?: Yes

Causes reversible?: Yes

Extreme fluctuations?: No

Population Information (Narrative): No population size estimates exist.

#### Subpopulations

Trend: Stable

Extreme fluctuations?: No

Severe fragmentation?: No

#### Habitat

System: Terrestrial

Habitat specialist: Yes

Habitat (narrative): Humid laurisilva forest on the northern slopes of Madeira Island.

Trend in extent, area or quality?: Stable

Justification for trend: The preferred habitat of the species, humid laurisilva forest, is not experiencing any decline in area and the invasive species present should not affect the spider populations.

##### Habitat

Habitat importance: Major Importance

Habitats: 1.9. Forest - Subtropical/Tropical Moist Montane

#### Habitat

Habitat importance: Major Importance

Habitats: 1.9. Forest - Subtropical/Tropical Moist Montane

#### Ecology

Size: 10-13 mm

Generation length (yr): 1

Dependency of single sp?: No

Ecology and traits (narrative): Active nocturnal ground hunter feeding mainly on small/medium size arthropods.

#### Threats

Justification for threats: Unknown threats.

##### Threats

Threat type: Past

Threats: 12. Other options - Other threat

#### Threats

Threat type: Past

Threats: 12. Other options - Other threat

#### Conservation

Justification for conservation actions: Most of the species range is inside the Madeira Natural Park.

##### Conservation actions

Conservation action type: In Place

Conservation actions: 1.1. Land/water protection - Site/area protection

#### Conservation actions

Conservation action type: In Place

Conservation actions: 1.1. Land/water protection - Site/area protection

#### Other

##### Use and trade

Use type: International

##### Ecosystem services

Ecosystem service type: Very important

##### Research needed

Research needed: 3.1. Monitoring - Population trends

Justification for research needed: Monitoring of population trends should be conducted to confirm species status.

#### Use and trade

Use type: International

#### Ecosystem services

Ecosystem service type: Very important

#### Research needed

Research needed: 3.1. Monitoring - Population trends

Justification for research needed: Monitoring of population trends should be conducted to confirm species status.

#### Viability analysis

### Mesiotelus maderianus

#### Species information

Scientific name: Mesiotelus maderianus

Species authority: Kulczynski, 1899

Kingdom: Animalia

Phylum: Arthropoda

Class: Arachnida

Order: Araneae

Family: Liocranidae

Region for assessment: Global

#### Geographic range

Biogeographic realm: Palearctic

Countries: Portugal

Map of records (Google Earth): Suppl. material 30

Basis of EOO and AOO: Species Distribution Model

Basis (narrative): There are only three records for the species, mostly old (Kulczyński 1899) plus a new record from Pico do Cidrão (unpublished). We still performed species distribution modeling to predict its potential range with confidence limits although these should be taken with caution. See methods for details.

Min Elevation/Depth (m): 650

Max Elevation/Depth (m): 1850

Range description: Known from few sites at high altitude, in laurisilva forest or above tree-line.

#### New occurrences

#### Extent of occurrence

EOO (km2): 28-256-720

Trend: Decline (inferred)

Justification for trend: One of the species habitats, humid laurisilva forest, is not experiencing any decline in area and the invasive species present should not affect the spider populations. The other habitat, mountain areas, experienced a severe wildfire in 2010 which might have reduced the species range.

Causes ceased?: Yes

Causes understood?: Yes

Causes reversible?: Yes

Extreme fluctuations?: No

#### Area of occupancy

Trend: Decline (inferred)

Justification for trend: One of the species habitats, humid laurisilva forest, is not experiencing any decline in area and the invasive species present should not affect the spider populations. The other habitat, mountain areas, experienced a severe wildfire in 2010 which might have reduced the species range.

Causes ceased?: Yes

Causes understood?: Yes

Causes reversible?: Yes

Extreme fluctuations?: No

AOO (km2): 28-256-720

#### Locations

Number of locations: 2

Justification for number of locations: A single wildfire event may affect the entire area above tree-line. The forest areas are in general not threatened.

Trend: Stable

Extreme fluctuations?: No

#### Population

Number of individuals: Unknown

Trend: Decline (inferred)

Justification for trend: One of the species habitats, humid laurisilva forest, is not experiencing any decline in area and the invasive species present should not affect the spider populations. The other habitat, mountain areas, experienced a severe wildfire in 2010 which might have reduced the species range.

Basis for decline: (c) a decline in area of occupancy, extent of occurrence and/or quality of habitat

Causes ceased?: Yes

Causes understood?: Yes

Causes reversible?: Yes

Extreme fluctuations?: No

Population Information (Narrative): No population size estimates exist.

#### Subpopulations

Number of subpopulations: Unknown

Trend: Decline (inferred)

Justification for trend: One of the species habitats, humid laurisilva forest, is not experiencing any decline in area and the invasive species present should not affect the spider populations. The other habitat, mountain areas, experienced a severe wildfire in 2010 which might have reduced the species population.

Extreme fluctuations?: No

Severe fragmentation?: No

#### Habitat

System: Terrestrial

Habitat specialist: No

Habitat (narrative): The only records of the species are from both laurisilva (Queimadas) and the mountain peaks (Pico do Cidrão).

Trend in extent, area or quality?: Decline (inferred)

Justification for trend: Although the laurisilva areas are extensive and mostly well-preserved, the mountain areas above the tree-line have suffered recent (2010) extensive wildfires that destroyed much of the native habitat. It is unknown whether these events have affected the species and to what proportion of its range, although this can be inferred.

##### Habitat

Habitat importance: Major Importance

Habitats: 1.9. Forest - Subtropical/Tropical Moist Montane6. Rocky areas (e.g. inland cliffs, mountain peaks)

#### Habitat

Habitat importance: Major Importance

Habitats: 1.9. Forest - Subtropical/Tropical Moist Montane6. Rocky areas (e.g. inland cliffs, mountain peaks)

#### Ecology

Size: 3 mm

Generation length (yr): 1

Dependency of single sp?: No

Ecology and traits (narrative): Active ground hunter feeding mainly on small size arthropods.

#### Threats

Justification for threats: Wildfires have negatively impacted the subpopulations above treeline in the past and may do it again in the future.

##### Threats

Threat type: Ongoing

Threats: 7.1. Natural system modifications - Fire & fire suppression

#### Threats

Threat type: Ongoing

Threats: 7.1. Natural system modifications - Fire & fire suppression

#### Conservation

Justification for conservation actions: Most of the species range is predicted to be inside the Madeira Natural Park.

##### Conservation actions

Conservation action type: In Place

Conservation actions: 1.1. Land/water protection - Site/area protection

#### Conservation actions

Conservation action type: In Place

Conservation actions: 1.1. Land/water protection - Site/area protection

#### Other

##### Use and trade

Use type: International

##### Ecosystem services

Ecosystem service type: Very important

##### Research needed

Research needed: 1.2. Research - Population size, distribution & trends1.3. Research - Life history & ecology1.5. Research - Threats3.1. Monitoring - Population trends3.4. Monitoring - Habitat trends

Justification for research needed: Given the high uncertainty the species range should be extensively studied. Furthermore, studies on the ecology of the species and how it was affected by wildfire is necessary. Afterwards, monitoring of population and habitat trends should be conducted to confirm species status.

#### Use and trade

Use type: International

#### Ecosystem services

Ecosystem service type: Very important

#### Research needed

Research needed: 1.2. Research - Population size, distribution & trends1.3. Research - Life history & ecology1.5. Research - Threats3.1. Monitoring - Population trends3.4. Monitoring - Habitat trends

Justification for research needed: Given the high uncertainty the species range should be extensively studied. Furthermore, studies on the ecology of the species and how it was affected by wildfire is necessary. Afterwards, monitoring of population and habitat trends should be conducted to confirm species status.

#### Viability analysis

### Meta barreti

#### Species information

Scientific name: Meta barreti

Species authority: Kulczynski, 1899

Kingdom: Animalia

Phylum: Arthropoda

Class: Arachnida

Order: Araneae

Family: Tetragnathidae

Region for assessment: Global

#### Geographic range

Biogeographic realm: Palearctic

Countries: Portugal

Map of records (Google Earth): Suppl. material 31

Basis of EOO and AOO: Species Distribution Model

Basis (narrative): Multiple collection sites are recorded for the species mostly in laurisilva forest, although none recently (Warburton 1892, Schmitz 1895, Kulczyński 1899, Bristowe 1925, Bacelar 1937, Schenkel 1938, Denis 1962, Wunderlich 1987, Wunderlich 1992). It was possible to perform species distribution modeling to predict its potential range with confidence limits. See methods for details.

Min Elevation/Depth (m): 50

Max Elevation/Depth (m): 1850

Range description: *Meta barreti* is (or was) known throughout the laurisilva forest that occupies about 20% of the island, mainly on its steep and humid northern slopes.

#### New occurrences

#### Extent of occurrence

EOO (km2): 0-352-899

Trend: Decline (inferred)

Justification for trend: The preferred habitat of the species, humid laurisilva forest, is not experiencing any decline in area. It should be noted, however, that the species has not been recorded for at least two decades despite extensive sampling. At the same time, the endemic congener *Meta stridulans* is now commonly seen after description in 1987, about the same time as *M. barreti* was last recorded. It is possible there was replacement of one species by the other, both single island endemics, although this is for now only suspected.

Causes ceased?: Unknown

Causes understood?: Unknown

Causes reversible?: Unknown

Extreme fluctuations?: Unknown

#### Area of occupancy

Trend: Decline (inferred)

Justification for trend: The preferred habitat of the species, humid laurisilva forest, is not experiencing any decline in area. It should be noted, however, that the species has not been recorded for at least two decades despite extensive sampling. At the same time, the endemic congener *M. stridulans* is now commonly seen after description in 1987, about the same time as *M. barreti* was last recorded. It is possible there was replacement of one species by the other, both single island endemics, although this is for now only suspected.

Causes ceased?: Unknown

Causes understood?: Unknown

Causes reversible?: Unknown

Extreme fluctuations?: Unknown

AOO (km2): 0-352-880

#### Locations

Number of locations: 0-1

Justification for number of locations: The species is not recorded for at least two decades despite extensive sampling. At the same time, the endemic congener *M. stridulans* is now very common in the same habitat and region. If this latter species replaced *M. barreti* it was a fast single event, although this can only be suspected.

Trend: Stable

Extreme fluctuations?: No

#### Population

Number of individuals: Unknown

Trend: Decline (inferred)

Justification for trend: The preferred habitat of the species, humid laurisilva forest, is not experiencing any decline in area. It should be noted, however, that the species is not recorded for at least two decades despite extensive sampling. At the same time, the endemic congener *M. stridulans* is now commonly seen after description in 1987, about the same time as *M. barreti* was last recorded. It is possible there was replacement of one species by the other, both single island endemics, although this, for now, is only suspected.

Basis for decline: (c) a decline in area of occupancy, extent of occurrence and/or quality of habitat(e) the effects of introduced taxa, hybridization, pathogens, pollutants, competitors or parasites.

Causes ceased?: Unknown

Causes understood?: Unknown

Causes reversible?: Unknown

Extreme fluctuations?: Unknown

Population Information (Narrative): No population size estimates exist.

#### Subpopulations

Number of subpopulations: Unknown

Trend: Decline (inferred)

Justification for trend: The preferred habitat of the species, humid laurisilva forest, is not experiencing any decline in area. It should be noted however that the species is not recorded for at least two decades despite extensive sampling. At the same time, the endemic congener *M. stridulans* is now commonly seen after description in 1987, about the same time as *M. barreti* was last recorded. It is possible there was replacement of one species by the other, both single island endemics, although this, for now, is only suspected.

Extreme fluctuations?: Unknown

Severe fragmentation?: Unknown

#### Habitat

System: Terrestrial

Habitat specialist: Yes

Habitat (narrative): Humid laurisilva forest on the northern slopes of Madeira Island.

Trend in extent, area or quality?: Decline (inferred)

Justification for trend: The preferred habitat of the species, humid laurisilva forest, is not experiencing any decline in area. It should be noted, however, that the species is not recorded for at least two decades despite extensive sampling. At the same time, the endemic congener *M. stridulans* is now commonly seen after description in 1987, about the same time as *M. barreti* was last recorded. It is possible there was replacement of one species by the other, both single island endemics, although this, for now, is only suspected.

##### Habitat

Habitat importance: Major Importance

Habitats: 1.9. Forest - Subtropical/Tropical Moist Montane

#### Habitat

Habitat importance: Major Importance

Habitats: 1.9. Forest - Subtropical/Tropical Moist Montane

#### Ecology

Size: 6 mm

Generation length (yr): 1

Dependency of single sp?: No

Ecology and traits (narrative): This species is an orb-web builder, possibly on the tree branches, feeding mainly on small insects. The congener *M. stridulans* is much larger (up to 11 mm), possibly giving it a competitive advantage.

#### Threats

Justification for threats: The endemic congener *M. stridulans* is now commonly seen after description in 1987, about the same time as *M. barreti* was last recorded. It is possible there was replacement of one species by the other, both single island endemics, although this is for now only suspected.

##### Threats

Threat type: Ongoing

Threats: 8.2. Invasive and other problematic species, genes & diseases - Problematic native species/diseases

#### Threats

Threat type: Ongoing

Threats: 8.2. Invasive and other problematic species, genes & diseases - Problematic native species/diseases

#### Conservation

Justification for conservation actions: All the species range is inside the Madeira Natural Park. If its apparent disappearance from the native range is confirmed some measures targeting species recovery should be implemented.

##### Conservation actions

Conservation action type: In Place

Conservation actions: 1.1. Land/water protection - Site/area protection

##### Conservation actions

Conservation action type: Needed

Conservation actions: 3.2. Species management - Species recovery

#### Conservation actions

Conservation action type: In Place

Conservation actions: 1.1. Land/water protection - Site/area protection

#### Conservation actions

Conservation action type: Needed

Conservation actions: 3.2. Species management - Species recovery

#### Other

##### Use and trade

Use type: International

##### Ecosystem services

Ecosystem service type: Very important

##### Research needed

Research needed: 1.2. Research - Population size, distribution & trends1.5. Research - Threats

Justification for research needed: The true distribution of the species and possible confusion with *Meta stridulans* should be clarified. Also research on the possible temporal replacement between these two species is needed.

#### Use and trade

Use type: International

#### Ecosystem services

Ecosystem service type: Very important

#### Research needed

Research needed: 1.2. Research - Population size, distribution & trends1.5. Research - Threats

Justification for research needed: The true distribution of the species and possible confusion with *Meta stridulans* should be clarified. Also research on the possible temporal replacement between these two species is needed.

#### Viability analysis

### Meta stridulans

#### Species information

Scientific name: Meta stridulans

Species authority: Wunderlich, 1987

Kingdom: Animalia

Phylum: Arthropoda

Class: Arachnida

Order: Araneae

Family: Tetragnathidae

Region for assessment: Global

#### Geographic range

Biogeographic realm: Palearctic

Countries: Portugal

Map of records (Google Earth): Suppl. material 32

Basis of EOO and AOO: Species Distribution Model

Basis (narrative): Multiple collection sites are recorded for the species, mostly recent and in laurisilva forest (Wunderlich 1987, Crespo et al. 2014b). It was possible to perform species distribution modeling to predict its potential range with confidence limits. See methods for details.

Min Elevation/Depth (m): 200

Max Elevation/Depth (m): 1400

Range description: *Meta stridulans* is known throughout the laurisilva forest that occupies about 20% of the island, mainly on its steep and humid northern slopes.

#### New occurrences

#### Extent of occurrence

EOO (km2): 98-336-832

Trend: Stable

Justification for trend: The preferred habitat of the species, humid laurisilva forest, is not experiencing any decline in area and the invasive species present should not affect the spider populations.

Causes ceased?: Yes

Causes understood?: Yes

Causes reversible?: Yes

Extreme fluctuations?: No

#### Area of occupancy

Trend: Stable

Justification for trend: The preferred habitat of the species, humid laurisilva forest, is not experiencing any decline in area and the invasive species present should not affect the spider populations.

Causes ceased?: Yes

Causes understood?: Yes

Causes reversible?: Yes

Extreme fluctuations?: No

AOO (km2): 96-324-812

#### Locations

Number of locations: 0

Justification for number of locations: No known threats to the species.

Trend: Stable

Extreme fluctuations?: No

#### Population

Number of individuals: Unknown

Trend: Stable

Justification for trend: The preferred habitat of the species, humid laurisilva forest, is not experiencing any decline in area and the invasive species present should not affect the spider populations.

Causes ceased?: Yes

Causes understood?: Yes

Causes reversible?: Yes

Extreme fluctuations?: No

Population Information (Narrative): No population size estimates exist.

#### Subpopulations

Trend: Stable

Extreme fluctuations?: No

Severe fragmentation?: No

#### Habitat

System: Terrestrial

Habitat specialist: Yes

Habitat (narrative): Humid laurisilva forest on the northern slopes of Madeira Island.

Trend in extent, area or quality?: Stable

##### Habitat

Habitat importance: Major Importance

Habitats: 1.9. Forest - Subtropical/Tropical Moist Montane

#### Habitat

Habitat importance: Major Importance

Habitats: 1.9. Forest - Subtropical/Tropical Moist Montane

#### Ecology

Size: 7-11 mm

Generation length (yr): 1

Dependency of single sp?: No

Ecology and traits (narrative): This species is an orb-web builder, possibly on the tree branches, feeding mainly on small-medium size insects.

#### Threats

Justification for threats: Unknown threats.

##### Threats

Threat type: Past

Threats: 12. Other options - Other threat

#### Threats

Threat type: Past

Threats: 12. Other options - Other threat

#### Conservation

Justification for conservation actions: Most of the species range is inside the Madeira Natural Park.

##### Conservation actions

Conservation action type: In Place

Conservation actions: 1.1. Land/water protection - Site/area protection

#### Conservation actions

Conservation action type: In Place

Conservation actions: 1.1. Land/water protection - Site/area protection

#### Other

##### Use and trade

Use type: International

##### Ecosystem services

Ecosystem service type: Less important

##### Research needed

Research needed: 3.1. Monitoring - Population trends

Justification for research needed: Monitoring of population trends should be conducted to confirm species status.

#### Use and trade

Use type: International

#### Ecosystem services

Ecosystem service type: Less important

#### Research needed

Research needed: 3.1. Monitoring - Population trends

Justification for research needed: Monitoring of population trends should be conducted to confirm species status.

#### Viability analysis

### Misumena nigromaculata

#### Species information

Scientific name: Misumena nigromaculata

Species authority: Denis, 1963

Kingdom: Animalia

Phylum: Arthropoda

Class: Arachnida

Order: Araneae

Family: Thomisidae

Region for assessment: Global

#### Geographic range

Biogeographic realm: Palearctic

Countries: Portugal

Map of records (Google Earth): Suppl. material 33

Basis of EOO and AOO: Unknown

Basis (narrative): The species EOO and AOO are unknown.

Range description: *Misumena nigromaculata* is known only from Funchal, south coast of Madeira Island, captured in October 1940 (Denis 1963) and was recently found with doubts in identification at Dunas da Piedade, Ponta de São Lourenco (unpublished).

#### New occurrences

#### Extent of occurrence

EOO (km2): Unknown

Trend: Unknown

Causes ceased?: Unknown

Causes understood?: Unknown

Causes reversible?: Unknown

Extreme fluctuations?: Unknown

#### Area of occupancy

Trend: Unknown

Causes ceased?: Unknown

Causes understood?: Unknown

Causes reversible?: Unknown

Extreme fluctuations?: Unknown

AOO (km2): Unknown

#### Locations

Number of locations: Unknown

Trend: Unknown

Extreme fluctuations?: Unknown

#### Population

Number of individuals: Unknown

Trend: Unknown

Causes ceased?: Unknown

Causes understood?: Unknown

Causes reversible?: Unknown

Extreme fluctuations?: Unknown

Population Information (Narrative): No population size estimates exist.

#### Subpopulations

Trend: Unknown

Extreme fluctuations?: Unknown

Severe fragmentation?: Unknown

#### Habitat

System: Terrestrial

Habitat specialist: Unknown

Habitat (narrative): The only place of confirmed presence in Funchal was largely occupied by farms and gardens at the time. The unconfirmed identification in Ponta de São Lourenco is on a semi-arid grassland.

Trend in extent, area or quality?: Unknown

##### Habitat

Habitat importance: Major Importance

Habitats: 4.5. Grassland - Subtropical/Tropical Dry16. Introduced vegetation

#### Habitat

Habitat importance: Major Importance

Habitats: 4.5. Grassland - Subtropical/Tropical Dry16. Introduced vegetation

#### Ecology

Size: 6 mm

Generation length (yr): 1

Dependency of single sp?: No

Ecology and traits (narrative): Unknown, but congeners are ambush hunters in low-vegetation, often seen waiting for prey on flowers.

#### Threats

Justification for threats: The only confirmed locality and location is now mostly residential area in the outskirts of Funchal.

##### Threats

Threat type: Ongoing

Threats: 1.1. Residential & commercial development - Housing & urban areas

#### Threats

Threat type: Ongoing

Threats: 1.1. Residential & commercial development - Housing & urban areas

#### Conservation

Justification for conservation actions: If a small range is confirmed in the future the species should benefit from a recovery plan. Yet, little information available precludes from advising on any concrete measures.

##### Conservation actions

Conservation action type: Needed

Conservation actions: 3.2. Species management - Species recovery

#### Conservation actions

Conservation action type: Needed

Conservation actions: 3.2. Species management - Species recovery

#### Other

##### Use and trade

Use type: International

##### Ecosystem services

Ecosystem service type: Very important

##### Research needed

Research needed: 1.2. Research - Population size, distribution & trends1.5. Research - Threats2.1. Conservation Planning - Species Action/Recovery Plan2.2. Conservation Planning - Area-based Management Plan

Justification for research needed: The true distribution of the species should be assessed along with possible threats along its range. If endangered, a species conservation plan encompassing recovery actions and area management should be needed to ensure the species survival.

#### Use and trade

Use type: International

#### Ecosystem services

Ecosystem service type: Very important

#### Research needed

Research needed: 1.2. Research - Population size, distribution & trends1.5. Research - Threats2.1. Conservation Planning - Species Action/Recovery Plan2.2. Conservation Planning - Area-based Management Plan

Justification for research needed: The true distribution of the species should be assessed along with possible threats along its range. If endangered, a species conservation plan encompassing recovery actions and area management should be needed to ensure the species survival.

#### Viability analysis

### Oecobius minor

#### Species information

Scientific name: Oecobius minor

Species authority: Kulczynski, 1909

Kingdom: Animalia

Phylum: Arthropoda

Class: Arachnida

Order: Araneae

Family: Oecobiidae

Region for assessment: Global

#### Geographic range

Biogeographic realm: Palearctic

Countries: Portugal

Map of records (Google Earth): Suppl. material 34

Basis of EOO and AOO: Unknown

Basis (narrative): The species EOO and AOO are unknown.

Range description: *Oecobius minor* is known only from the mountainous area south of Faial on the northern coast of Madeira Island, captured in undefined date (Wunderlich 1992). The only other record is from the same island in undefined locality (Kulczyński 1909).

#### New occurrences

#### Extent of occurrence

EOO (km2): Unknown

Trend: Unknown

Causes ceased?: Unknown

Causes understood?: Unknown

Causes reversible?: Unknown

Extreme fluctuations?: Unknown

#### Area of occupancy

Trend: Unknown

Causes ceased?: Unknown

Causes understood?: Unknown

Causes reversible?: Unknown

Extreme fluctuations?: Unknown

AOO (km2): Unknown

#### Locations

Number of locations: Unknown

Trend: Unknown

Extreme fluctuations?: Unknown

#### Population

Number of individuals: Unknown

Trend: Unknown

Causes ceased?: Unknown

Causes understood?: Unknown

Causes reversible?: Unknown

Extreme fluctuations?: Unknown

Population Information (Narrative): No population size estimates exist.

#### Subpopulations

Trend: Unknown

Extreme fluctuations?: Unknown

Severe fragmentation?: Unknown

#### Habitat

System: Terrestrial

Habitat specialist: Unknown

Habitat (narrative): The only known locality probably is in laurisilva forest.

Trend in extent, area or quality?: Unknown

##### Habitat

Habitat importance: Major Importance

Habitats: 1.9. Forest - Subtropical/Tropical Moist Montane

#### Habitat

Habitat importance: Major Importance

Habitats: 1.9. Forest - Subtropical/Tropical Moist Montane

#### Ecology

Size: 2 mm

Generation length (yr): 1

Dependency of single sp?: No

Ecology and traits (narrative): Unknown, but congeners build small flat webs over rocks or trunks where they hunt for small insects.

#### Threats

Justification for threats: Unknown threats.

##### Threats

Threat type: Past

Threats: 12. Other options - Other threat

#### Threats

Threat type: Past

Threats: 12. Other options - Other threat

#### Conservation

Justification for conservation actions: The known species range is inside the Madeira Natural Park.

##### Conservation actions

Conservation action type: In Place

Conservation actions: 1.1. Land/water protection - Site/area protection

#### Conservation actions

Conservation action type: In Place

Conservation actions: 1.1. Land/water protection - Site/area protection

#### Other

##### Use and trade

Use type: International

##### Ecosystem services

Ecosystem service type: Very important

##### Research needed

Research needed: 1.2. Research - Population size, distribution & trends1.5. Research - Threats

Justification for research needed: The true distribution of the species should be assessed along with possible threats along its range.

#### Use and trade

Use type: International

#### Ecosystem services

Ecosystem service type: Very important

#### Research needed

Research needed: 1.2. Research - Population size, distribution & trends1.5. Research - Threats

Justification for research needed: The true distribution of the species should be assessed along with possible threats along its range.

#### Viability analysis

### Oecobius selvagensis

#### Species information

Scientific name: Oecobius selvagensis

Species authority: Wunderlich, 1995

Kingdom: Animalia

Phylum: Arthropoda

Class: Arachnida

Order: Araneae

Family: Oecobiidae

Region for assessment: Global

#### Geographic range

Biogeographic realm: Palearctic

Countries: Portugal

Map of records (Google Earth): Suppl. material 35

Basis of EOO and AOO: Observed

Basis (narrative): The restricted distribution of the species allows to know its EOO and AOO with reasonable confidence.

Min Elevation/Depth (m): 0

Max Elevation/Depth (m): 160

Range description: The species is probably restricted to the Selvagem Grande Island between Madeira and the Canary Islands, where it was found in an unnamed erosion coastal cave in 1958 (Denis 1963, Rambla 1978, Wunderlich 1992). Two caves are now the entire known range of the species.

#### New occurrences

#### Extent of occurrence

EOO (km2): 4

Trend: Decline (inferred)

Justification for trend: The cosmopolitan congener *O. navus* is now commonly seen, even in caves, after being detected on the island for the first time before 1978. It is possible there is a gradual replacement of one species by the other, although this is for now only suspected.

Causes ceased?: No

Causes understood?: Yes

Causes reversible?: No

Extreme fluctuations?: No

#### Area of occupancy

Trend: Decline (inferred)

Justification for trend: The cosmopolitan congener *O. navus* is now commonly seen, even in caves, after being detected on the island for the first time before 1978. It is possible there is a gradual replacement of one species by the other, although this is for now only suspected.

Causes ceased?: No

Causes understood?: Yes

Causes reversible?: No

Extreme fluctuations?: No

AOO (km2): 4

#### Locations

Number of locations: 1

Justification for number of locations: If the suspicion that the introduction of *O. navus* is affecting *O. selvagensis* is confirmed, there is a single location derived from the spread of the invasive species in a single event.

Trend: Stable

Justification for trend: Probably stable for the last 10 years.

Extreme fluctuations?: No

#### Population

Number of individuals: Unknown

Trend: Decline (inferred)

Justification for trend: The cosmopolitan congener *O. navus* is now commonly seen, even in caves, after being detected on the island for the first time before 1978. It is possible there is a gradual replacement of one species by the other, although this is for now only suspected.

Basis for decline: (e) the effects of introduced taxa, hybridization, pathogens, pollutants, competitors or parasites.

Causes ceased?: No

Causes understood?: Yes

Causes reversible?: No

Extreme fluctuations?: No

Population Information (Narrative): No population size estimates exist.

#### Subpopulations

Number of subpopulations: 1

Trend: Stable

Justification for trend: A single subpopulation is historically known.

Extreme fluctuations?: No

Severe fragmentation?: No

#### Habitat

System: Terrestrial

Habitat specialist: Yes

Habitat (narrative): Only know from two caves.

Trend in extent, area or quality?: Decline (inferred)

Justification for trend: The cosmopolitan congener *O. navus* is now commonly seen, even in caves, after being detected on the island for the first time before 1978. It is possible there is a gradual replacement of one species by the other, although this, for now, is only suspected.

##### Habitat

Habitat importance: Major Importance

Habitats: 13.2. Marine Coastal/Supratidal - Coastal Caves/Karst

#### Habitat

Habitat importance: Major Importance

Habitats: 13.2. Marine Coastal/Supratidal - Coastal Caves/Karst

#### Ecology

Size: 2 mm

Generation length (yr): 1

Dependency of single sp?: No

Ecology and traits (narrative): It is probable that this is a species that lives underground due to the two known localities being inside of caves and it being largely depigmented. Congeners build small flat webs where they hunt for small insects.

#### Threats

Justification for threats: The cosmopolitan *O. navus* may be outcompeting the species in part of its range.

##### Threats

Threat type: Ongoing

Threats: 8.1. Invasive and other problematic species, genes & diseases - Invasive non-native/alien species/diseases

#### Threats

Threat type: Ongoing

Threats: 8.1. Invasive and other problematic species, genes & diseases - Invasive non-native/alien species/diseases

#### Conservation

Justification for conservation actions: The island of Selvagem Grande is part of the Selvagens Nature Reserve. If competition is confirmed, the invasive *O. navus* should be controlled. As this task is probably impossible, ex-situ conservation with eventual re-introduction and recovery might be the only feasible measure to prevent further reduction of *O. selvagensis*.

##### Conservation actions

Conservation action type: In Place

Conservation actions: 1.1. Land/water protection - Site/area protection

##### Conservation actions

Conservation action type: Needed

Conservation actions: 2.2. Land/water management - Invasive/problematic species control3.2. Species management - Species recovery3.3. Species management - Species re-introduction3.4. Species management - Ex-situ conservation

#### Conservation actions

Conservation action type: In Place

Conservation actions: 1.1. Land/water protection - Site/area protection

#### Conservation actions

Conservation action type: Needed

Conservation actions: 2.2. Land/water management - Invasive/problematic species control3.2. Species management - Species recovery3.3. Species management - Species re-introduction3.4. Species management - Ex-situ conservation

#### Other

##### Use and trade

Use type: International

##### Ecosystem services

Ecosystem service type: Very important

##### Research needed

Research needed: 1.2. Research - Population size, distribution & trends1.5. Research - Threats2.1. Conservation Planning - Species Action/Recovery Plan2.2. Conservation Planning - Area-based Management Plan

Justification for research needed: The current distribution of the species and possible threats from the invasive congener should be thoroughly studied. If outcompeted, *O. selvagensis* should be the target of a species conservation plan with consequent area management actions.

#### Use and trade

Use type: International

#### Ecosystem services

Ecosystem service type: Very important

#### Research needed

Research needed: 1.2. Research - Population size, distribution & trends1.5. Research - Threats2.1. Conservation Planning - Species Action/Recovery Plan2.2. Conservation Planning - Area-based Management Plan

Justification for research needed: The current distribution of the species and possible threats from the invasive congener should be thoroughly studied. If outcompeted, *O. selvagensis* should be the target of a species conservation plan with consequent area management actions.

#### Viability analysis

### Parapelecopsis mediocris

#### Species information

Scientific name: Parapelecopsis mediocris

Species authority: (Kulczynski, 1899)

Kingdom: Animalia

Phylum: Arthropoda

Class: Arachnida

Order: Araneae

Family: Linyphiidae

Taxonomic notes: Possible junior synonym of *Parapelecopsis nemoralioides* (O. Pickard-Cambridge, 1884) (Wunderlich 1992)

Region for assessment: Global

#### Geographic range

Biogeographic realm: Palearctic

Countries: Portugal

Map of records (Google Earth): Suppl. material 36

Basis of EOO and AOO: Unknown

Basis (narrative): The species EOO and AOO are unknown.

Range description: Described from undefined locality in Madeira Island (Kulczyński 1899)

#### New occurrences

#### Extent of occurrence

EOO (km2): Unknown

Trend: Unknown

Causes ceased?: Unknown

Causes understood?: Unknown

Causes reversible?: Unknown

Extreme fluctuations?: Unknown

#### Area of occupancy

Trend: Unknown

Causes ceased?: Unknown

Causes understood?: Unknown

Causes reversible?: Unknown

Extreme fluctuations?: Unknown

AOO (km2): Unknown

#### Locations

Number of locations: Unknown

Trend: Unknown

Extreme fluctuations?: Unknown

#### Population

Number of individuals: Unknown

Trend: Unknown

Causes ceased?: Unknown

Causes understood?: Unknown

Causes reversible?: Unknown

Extreme fluctuations?: Unknown

Population Information (Narrative): The species population size and trend are unknown.

#### Subpopulations

Trend: Unknown

Extreme fluctuations?: Unknown

Severe fragmentation?: Unknown

#### Habitat

System: Terrestrial

Habitat specialist: Unknown

Habitat (narrative): The species habitat is unknown.

Trend in extent, area or quality?: Unknown

##### Habitat

Habitat importance: Major Importance

Habitats: 18. Unknown

#### Habitat

Habitat importance: Major Importance

Habitats: 18. Unknown

#### Ecology

Size: 2 mm

Generation length (yr): 1

Dependency of single sp?: No

Ecology and traits (narrative): Unknown.

#### Threats

Justification for threats: Unknown threats.

##### Threats

Threat type: Past

Threats: 12. Other options - Other threat

#### Threats

Threat type: Past

Threats: 12. Other options - Other threat

#### Conservation

##### Conservation actions

#### Conservation actions

#### Other

##### Use and trade

Use type: International

##### Ecosystem services

Ecosystem service type: Very important

##### Research needed

Research needed: 1.1. Research - Taxonomy1.2. Research - Population size, distribution & trends1.3. Research - Life history & ecology1.5. Research - Threats

Justification for research needed: The species is not found since original description in 1899 (Kulczyński 1899) and needs, first of all, taxonomic clarification to confirm synonymy with *P. nemoralioides*. If valid, basic information would be needed on its distribution, ecology and possible threats.

#### Use and trade

Use type: International

#### Ecosystem services

Ecosystem service type: Very important

#### Research needed

Research needed: 1.1. Research - Taxonomy1.2. Research - Population size, distribution & trends1.3. Research - Life history & ecology1.5. Research - Threats

Justification for research needed: The species is not found since original description in 1899 (Kulczyński 1899) and needs, first of all, taxonomic clarification to confirm synonymy with *P. nemoralioides*. If valid, basic information would be needed on its distribution, ecology and possible threats.

#### Viability analysis

### Philodromus insulanus

#### Species information

Scientific name: Philodromus insulanus

Species authority: Kulczynski, 1905

Kingdom: Animalia

Phylum: Arthropoda

Class: Arachnida

Order: Araneae

Family: Philodromidae

Region for assessment: Global

#### Geographic range

Biogeographic realm: Palearctic

Countries: Portugal

Map of records (Google Earth): Suppl. material 37

Basis of EOO and AOO: Species Distribution Model

Basis (narrative): Multiple collection sites are recorded for the species, mostly recent and in laurisilva forest (Kulczyński 1905, Schenkel 1938, Denis 1962, Crespo et al. 2014b). It was possible to perform species distribution modeling to predict its potential range with confidence limits. See methods for details.

Min Elevation/Depth (m): 300

Max Elevation/Depth (m): 1750

Range description: *Philodromus insulanus* is known throughout the laurisilva forest that occupies about 20% of the island, mainly on its steep and humid northern slopes.

#### New occurrences

#### Extent of occurrence

EOO (km2): 201-419-782

Trend: Stable

Justification for trend: The preferred habitat of the species, humid laurisilva forest, is not experiencing any decline in area and the invasive species present should not affect the spider populations.

Causes ceased?: Yes

Causes understood?: Yes

Causes reversible?: Yes

Extreme fluctuations?: No

#### Area of occupancy

Trend: Stable

Justification for trend: The preferred habitat of the species, humid laurisilva forest, is not experiencing any decline in area and the invasive species present should not affect the spider populations.

Causes ceased?: Yes

Causes understood?: Yes

Causes reversible?: Yes

Extreme fluctuations?: No

AOO (km2): 132-396-776

#### Locations

Number of locations: 0

Justification for number of locations: No known threats to the species.

Trend: Stable

Extreme fluctuations?: No

#### Population

Number of individuals: Unknown

Trend: Stable

Justification for trend: The preferred habitat of the species, humid laurisilva forest, is not experiencing any decline in area and the invasive species present should not affect the spider populations.

Causes ceased?: Yes

Causes understood?: Yes

Causes reversible?: Yes

Extreme fluctuations?: No

Population Information (Narrative): No population size estimates exist.

#### Subpopulations

Trend: Stable

Extreme fluctuations?: No

Severe fragmentation?: No

#### Habitat

System: Terrestrial

Habitat specialist: Yes

Habitat (narrative): Humid laurisilva forest on the northern slopes of Madeira Island.

Trend in extent, area or quality?: Stable

Justification for trend: The preferred habitat of the species, humid laurisilva forest, is not experiencing any decline in area and the invasive species present should not affect the spider populations.

##### Habitat

Habitat importance: Major Importance

Habitats: 1.9. Forest - Subtropical/Tropical Moist Montane

#### Habitat

Habitat importance: Major Importance

Habitats: 1.9. Forest - Subtropical/Tropical Moist Montane

#### Ecology

Size: 5-12 mm

Generation length (yr): 1

Dependency of single sp?: No

Ecology and traits (narrative): The species is an active hunter at low to high vegetation feeding mainly on small size arthropods.

#### Threats

Justification for threats: Unknown threats.

##### Threats

Threat type: Past

Threats: 12. Other options - Other threat

#### Threats

Threat type: Past

Threats: 12. Other options - Other threat

#### Conservation

Justification for conservation actions: Most of the species range is inside the Madeira Natural Park.

##### Conservation actions

Conservation action type: In Place

Conservation actions: 1.1. Land/water protection - Site/area protection

#### Conservation actions

Conservation action type: In Place

Conservation actions: 1.1. Land/water protection - Site/area protection

#### Other

##### Use and trade

Use type: International

##### Ecosystem services

Ecosystem service type: Very important

##### Research needed

Research needed: 3.1. Monitoring - Population trends

Justification for research needed: Monitoring of population trends should be conducted to confirm species status.

#### Use and trade

Use type: International

#### Ecosystem services

Ecosystem service type: Very important

#### Research needed

Research needed: 3.1. Monitoring - Population trends

Justification for research needed: Monitoring of population trends should be conducted to confirm species status.

#### Viability analysis

### Philodromus simillimus

#### Species information

Scientific name: Philodromus simillimus

Species authority: Denis, 1962

Kingdom: Animalia

Phylum: Arthropoda

Class: Arachnida

Order: Araneae

Family: Philodromidae

Region for assessment: Global

#### Geographic range

Biogeographic realm: Palearctic

Countries: Portugal

Map of records (Google Earth): Suppl. material 38

Basis of EOO and AOO: Unknown

Basis (narrative): The species EOO and AOO are unknown.

Min Elevation/Depth (m): 1860

Max Elevation/Depth (m): 1860

Range description: Only known from Pico Ruivo, the highest mountain in Madeira Island with 1861 m altitude (Denis 1962). A single female was captured in April 1957.

#### New occurrences

#### Extent of occurrence

EOO (km2): Unknown

Trend: Unknown

Causes ceased?: Unknown

Causes understood?: Unknown

Causes reversible?: Unknown

Extreme fluctuations?: Unknown

#### Area of occupancy

Trend: Unknown

Causes ceased?: Unknown

Causes understood?: Unknown

Causes reversible?: Unknown

Extreme fluctuations?: Unknown

AOO (km2): Unknown

#### Locations

Number of locations: Unknown

Trend: Unknown

Extreme fluctuations?: Unknown

#### Population

Number of individuals: Unknown

Trend: Unknown

Causes ceased?: Unknown

Causes understood?: Unknown

Causes reversible?: Unknown

Extreme fluctuations?: Unknown

Population Information (Narrative): No population size estimates exist.

#### Subpopulations

Trend: Unknown

Extreme fluctuations?: Unknown

Severe fragmentation?: Unknown

#### Habitat

System: Terrestrial

Habitat specialist: Unknown

Habitat (narrative): The single site where the species was sampled is in high mountain above tree-line.

Trend in extent, area or quality?: Unknown

##### Habitat

Habitat importance: Major Importance

Habitats: 6. Rocky areas (e.g. inland cliffs, mountain peaks)

#### Habitat

Habitat importance: Major Importance

Habitats: 6. Rocky areas (e.g. inland cliffs, mountain peaks)

#### Ecology

Size: 5 mm

Generation length (yr): 1

Dependency of single sp?: No

Ecology and traits (narrative): Nothing is known about the species but congeners are active hunters mainly feeding on small arthropods.

#### Threats

Justification for threats: If this species is restricted to the high peaks of Madeira recent wildfires may have affected its population. Given the lack of information this is purely speculative.

##### Threats

Threat type: Ongoing

Threats: 7.1. Natural system modifications - Fire & fire suppression

#### Threats

Threat type: Ongoing

Threats: 7.1. Natural system modifications - Fire & fire suppression

#### Conservation

Justification for conservation actions: If only living in the mountain peaks of Madeira, this species habitat is protected by the Madeira Natural Park.

##### Conservation actions

Conservation action type: In Place

Conservation actions: 1.1. Land/water protection - Site/area protection

#### Conservation actions

Conservation action type: In Place

Conservation actions: 1.1. Land/water protection - Site/area protection

#### Other

##### Use and trade

Use type: International

##### Ecosystem services

Ecosystem service type: Very important

##### Research needed

Research needed: 1.1. Research - Taxonomy1.2. Research - Population size, distribution & trends1.3. Research - Life history & ecology1.5. Research - Threats

Justification for research needed: Individuals of this species are not found since the original description (Denis 1962) and it needs, first of all, taxonomic clarification. If valid, basic information would be needed on its distribution, ecology and possible threats.

#### Use and trade

Use type: International

#### Ecosystem services

Ecosystem service type: Very important

#### Research needed

Research needed: 1.1. Research - Taxonomy1.2. Research - Population size, distribution & trends1.3. Research - Life history & ecology1.5. Research - Threats

Justification for research needed: Individuals of this species are not found since the original description (Denis 1962) and it needs, first of all, taxonomic clarification. If valid, basic information would be needed on its distribution, ecology and possible threats.

#### Viability analysis

### Pholcus dentatus

#### Species information

Scientific name: Pholcus dentatus

Species authority: Wunderlich, 1995

Kingdom: Animalia

Phylum: Arthropoda

Class: Arachnida

Order: Araneae

Family: Pholcidae

Region for assessment: Global

#### Geographic range

Biogeographic realm: Palearctic

Countries: Portugal

Map of records (Google Earth): Suppl. material 39

Basis of EOO and AOO: Unknown

Basis (narrative): The species EOO and AOO are unkown.

Min Elevation/Depth (m): 300

Max Elevation/Depth (m): 450

Range description: *Pholcus dentatus* is known from only two sites separated by 25 kms in the northern coast of Madeira Island between 300 and 450 meters elevation, Fonte da Pedra and Ribeira da Janela (Wunderlich 1995).

#### New occurrences

#### Extent of occurrence

EOO (km2): Unknown

Trend: Unknown

Causes ceased?: Unknown

Causes understood?: Unknown

Causes reversible?: Unknown

Extreme fluctuations?: Unknown

#### Area of occupancy

Trend: Unknown

Causes ceased?: Unknown

Causes understood?: Unknown

Causes reversible?: Unknown

Extreme fluctuations?: Unknown

AOO (km2): Unknown

#### Locations

Number of locations: Unknown

Trend: Unknown

Extreme fluctuations?: Unknown

#### Population

Number of individuals: Unknown

Trend: Unknown

Causes ceased?: Unknown

Causes understood?: Unknown

Causes reversible?: Unknown

Extreme fluctuations?: Unknown

Population Information (Narrative): No population size estimates exist.

#### Subpopulations

Trend: Unknown

Extreme fluctuations?: Unknown

Severe fragmentation?: Unknown

#### Habitat

System: Terrestrial

Habitat specialist: Unknown

Habitat (narrative): Habitat was never specified.

Trend in extent, area or quality?: Unknown

##### Habitat

Habitat importance: Major Importance

Habitats: 18. Unknown

#### Habitat

Habitat importance: Major Importance

Habitats: 18. Unknown

#### Ecology

Size: 4-5 mm

Generation length (yr): 1

Dependency of single sp?: No

Ecology and traits (narrative): The species ecology is unknown but almost certainly a tangle-web builder feeding on small arthropods.

#### Threats

Justification for threats: Unknown threats.

##### Threats

Threat type: Past

Threats: 12. Other options - Other threat

#### Threats

Threat type: Past

Threats: 12. Other options - Other threat

#### Conservation

##### Conservation actions

Conservation action type: In Place

#### Conservation actions

Conservation action type: In Place

#### Other

##### Use and trade

Use type: International

##### Ecosystem services

Ecosystem service type: Very important

##### Research needed

Research needed: 1.2. Research - Population size, distribution & trends1.3. Research - Life history & ecology1.5. Research - Threats

Justification for research needed: Basic information is needed on its distribution, ecology and possible threats.

#### Use and trade

Use type: International

#### Ecosystem services

Ecosystem service type: Very important

#### Research needed

Research needed: 1.2. Research - Population size, distribution & trends1.3. Research - Life history & ecology1.5. Research - Threats

Justification for research needed: Basic information is needed on its distribution, ecology and possible threats.

#### Viability analysis

### Pholcus madeirensis

#### Species information

Scientific name: Pholcus madeirensis

Species authority: Wunderlich, 1987

Kingdom: Animalia

Phylum: Arthropoda

Class: Arachnida

Order: Araneae

Family: Pholcidae

Region for assessment: Global

#### Geographic range

Biogeographic realm: Palearctic

Countries: Portugal

Map of records (Google Earth): Suppl. material 40

Basis of EOO and AOO: Species Distribution Model

Basis (narrative): Multiple collection sites are recorded for the species, mostly recent and in laurisilva forest (Wunderlich, 1987, unpublished). It was possible to perform species distribution modeling to predict its potential range with confidence limits. See methods for details.

Min Elevation/Depth (m): 350

Max Elevation/Depth (m): 1850

Range description: *Pholcus madeirensis* was first described from the southern slopes of Madeira, including near Funchal, in undescribed habitat (Wunderlich 1987). More recently it was found in several laurisilva forest sites of the northern slopes.

#### New occurrences

#### Extent of occurrence

EOO (km2): 47-332-761

Trend: Stable

Justification for trend: The preferred habitat of the species, humid laurisilva forest, is not experiencing any decline in area and the invasive species present should not affect the spider populations.

Causes ceased?: Yes

Causes understood?: Yes

Causes reversible?: Yes

Extreme fluctuations?: No

#### Area of occupancy

Trend: Stable

Justification for trend: The preferred habitat of the species, humid laurisilva forest, is not experiencing any decline in area and the invasive species present should not affect the spider populations.

Causes ceased?: Yes

Causes understood?: Yes

Causes reversible?: Yes

Extreme fluctuations?: No

AOO (km2): 12-332-752

#### Locations

Number of locations: 0

Justification for number of locations: No known threats to the species.

Trend: Stable

Extreme fluctuations?: No

#### Population

Number of individuals: Unknown

Trend: Stable

Justification for trend: The preferred habitat of the species, humid laurisilva forest, is not experiencing any decline in area and the invasive species present should not affect the spider populations.

Causes ceased?: Yes

Causes understood?: Yes

Causes reversible?: Yes

Extreme fluctuations?: No

Population Information (Narrative): No population size estimates exist.

#### Subpopulations

Trend: Stable

Extreme fluctuations?: No

Severe fragmentation?: No

#### Habitat

System: Terrestrial

Habitat specialist: No

Habitat (narrative): Mainly humid laurisilva forest on the northern slopes of Madeira Island, but also known from the southern slopes in undescribed habitat.

Trend in extent, area or quality?: Stable

Justification for trend: The preferred habitat of the species, humid laurisilva forest, is not experiencing any decline in area and the invasive species present should not affect the spider populations.

##### Habitat

Habitat importance: Major Importance

Habitats: 1.9. Forest - Subtropical/Tropical Moist Montane18. Unknown

#### Habitat

Habitat importance: Major Importance

Habitats: 1.9. Forest - Subtropical/Tropical Moist Montane18. Unknown

#### Ecology

Size: 5 mm

Generation length (yr): 1

Dependency of single sp?: No

Ecology and traits (narrative): The ecology of this species is unknown but almost certainly a tangle-web builder feeding on small arthropods.

#### Threats

Justification for threats: Unknown threats.

##### Threats

Threat type: Past

Threats: 12. Other options - Other threat

#### Threats

Threat type: Past

Threats: 12. Other options - Other threat

#### Conservation

Justification for conservation actions: Part of its range is inside the Madeira Natural Park.

##### Conservation actions

Conservation action type: In Place

Conservation actions: 1.1. Land/water protection - Site/area protection

#### Conservation actions

Conservation action type: In Place

Conservation actions: 1.1. Land/water protection - Site/area protection

#### Other

##### Use and trade

Use type: International

##### Ecosystem services

Ecosystem service type: Very important

##### Research needed

Research needed: 1.2. Research - Population size, distribution & trends1.3. Research - Life history & ecology3.1. Monitoring - Population trends

Justification for research needed: Monitoring of population trends should be conducted to confirm species status. As the preferred habitat outside laurisilva forest is unknown, research on distribution and preferred habitats should be a priority.

#### Use and trade

Use type: International

#### Ecosystem services

Ecosystem service type: Very important

#### Research needed

Research needed: 1.2. Research - Population size, distribution & trends1.3. Research - Life history & ecology3.1. Monitoring - Population trends

Justification for research needed: Monitoring of population trends should be conducted to confirm species status. As the preferred habitat outside laurisilva forest is unknown, research on distribution and preferred habitats should be a priority.

#### Viability analysis

### Pholcus magnus

#### Species information

Scientific name: Pholcus magnus

Species authority: Wunderlich, 1987

Kingdom: Animalia

Phylum: Arthropoda

Class: Arachnida

Order: Araneae

Family: Pholcidae

Region for assessment: Global

#### Geographic range

Biogeographic realm: Palearctic

Countries: Portugal

Map of records (Google Earth): Suppl. material 41

Basis of EOO and AOO: Unknown

Basis (narrative): The EOO and AOO of this species are unknown.

Min Elevation/Depth (m): 700

Max Elevation/Depth (m): 700

Range description: Only known from Portela, on eastern Madeira Island (Wunderlich 1987), captured under a bridge before 1987.

#### New occurrences

#### Extent of occurrence

EOO (km2): Unknown

Trend: Unknown

Causes ceased?: Unknown

Causes understood?: Unknown

Causes reversible?: Unknown

Extreme fluctuations?: Unknown

#### Area of occupancy

Trend: Unknown

Causes ceased?: Unknown

Causes understood?: Unknown

Causes reversible?: Unknown

Extreme fluctuations?: Unknown

AOO (km2): Unknown

#### Locations

Number of locations: Unknown

Trend: Unknown

Extreme fluctuations?: Unknown

#### Population

Number of individuals: Unknown

Trend: Unknown

Causes ceased?: Unknown

Causes understood?: Unknown

Causes reversible?: Unknown

Extreme fluctuations?: Unknown

Population Information (Narrative): No population size estimates exist.

#### Subpopulations

Trend: Unknown

Extreme fluctuations?: Unknown

Severe fragmentation?: Unknown

#### Habitat

System: Terrestrial

Habitat specialist: Unknown

Habitat (narrative): Only known from under a bridge surrounded by undescribed habitat (Wunderlich 1987).

Trend in extent, area or quality?: Unknown

##### Habitat

Habitat importance: Major Importance

Habitats: 18. Unknown

#### Habitat

Habitat importance: Major Importance

Habitats: 18. Unknown

#### Ecology

Size: 5-6 mm

Generation length (yr): 1

Dependency of single sp?: No

Ecology and traits (narrative): The ecology of the species is unknown but almost certainly a tangle-web builder feeding on small arthropods.

#### Threats

Justification for threats: Unknown threats.

##### Threats

Threat type: Past

Threats: 12. Other options - Other threat

#### Threats

Threat type: Past

Threats: 12. Other options - Other threat

#### Conservation

##### Conservation actions

#### Conservation actions

#### Other

##### Use and trade

Use type: International

##### Ecosystem services

Ecosystem service type: Very important

##### Research needed

Research needed: 1.2. Research - Population size, distribution & trends1.3. Research - Life history & ecology1.5. Research - Threats

Justification for research needed: Basic information is needed on its distribution, ecology and possible threats.

#### Use and trade

Use type: International

#### Ecosystem services

Ecosystem service type: Very important

#### Research needed

Research needed: 1.2. Research - Population size, distribution & trends1.3. Research - Life history & ecology1.5. Research - Threats

Justification for research needed: Basic information is needed on its distribution, ecology and possible threats.

#### Viability analysis

### Pholcus parvus

#### Species information

Scientific name: Pholcus parvus

Species authority: Wunderlich, 1987

Kingdom: Animalia

Phylum: Arthropoda

Class: Arachnida

Order: Araneae

Family: Pholcidae

Region for assessment: Global

#### Geographic range

Biogeographic realm: Palearctic

Countries: Portugal

Map of records (Google Earth): Suppl. material 42

Basis of EOO and AOO: Observed

Basis (narrative): Four collection sites are recorded for the species, most recently in laurisilva forest close to the northern coast of Madeira (Wunderlich 1987, Wunderlich 1995). It was possible to perform species distribution modeling to predict its potential range with confidence limits. See methods for details.

Min Elevation/Depth (m): 50

Max Elevation/Depth (m): 1150

Range description: *Pholcus parvus* is known from a few sites in laurisilva forest or nearby, always in valleys close to the northern coast of Madeira Island.

#### New occurrences

#### Extent of occurrence

EOO (km2): 76-213-912

Trend: Stable

Justification for trend: The preferred habitat of the species, humid laurisilva forest, is not experiencing any decline in area and the invasive species present should not affect the spider populations.

Causes ceased?: Yes

Causes understood?: Yes

Causes reversible?: Yes

Extreme fluctuations?: No

#### Area of occupancy

Trend: Stable

Justification for trend: The preferred habitat of the species, humid laurisilva forest, is not experiencing any decline in area and the invasive species present should not affect the spider populations.

Causes ceased?: Yes

Causes understood?: Yes

Causes reversible?: Yes

Extreme fluctuations?: No

AOO (km2): 16-204-912

#### Locations

Number of locations: 0

Justification for number of locations: No known threats to the species.

Trend: Stable

Extreme fluctuations?: No

#### Population

Number of individuals: Unknown

Trend: Stable

Justification for trend: The preferred habitat of the species, humid laurisilva forest, is not experiencing any decline in area and the invasive species present should not affect the spider populations.

Causes ceased?: Yes

Causes understood?: Yes

Causes reversible?: Yes

Extreme fluctuations?: No

Population Information (Narrative): No population size estimates exist.

#### Subpopulations

Trend: Stable

Extreme fluctuations?: No

Severe fragmentation?: No

#### Habitat

System: Terrestrial

Habitat specialist: Yes

Habitat (narrative): Humid laurisilva forest on the northern valleys of Madeira Island.

Trend in extent, area or quality?: Stable

Justification for trend: The preferred habitat of the species, humid laurisilva forest, is not experiencing any decline in area and the invasive species present should not affect the spider populations.

##### Habitat

Habitat importance: Major Importance

Habitats: 1.9. Forest - Subtropical/Tropical Moist Montane

#### Habitat

Habitat importance: Major Importance

Habitats: 1.9. Forest - Subtropical/Tropical Moist Montane

#### Ecology

Size: 4-5 mm

Generation length (yr): 1

Dependency of single sp?: No

Ecology and traits (narrative): The ecology of the species is unknown but almost certainly a tangle-web builder feeding on small arthropods.

#### Threats

Justification for threats: Unknown threats.

##### Threats

Threat type: Past

Threats: 12. Other options - Other threat

#### Threats

Threat type: Past

Threats: 12. Other options - Other threat

#### Conservation

Justification for conservation actions: Most of the species range is inside the Madeira Natural Park.

##### Conservation actions

Conservation action type: In Place

Conservation actions: 1.1. Land/water protection - Site/area protection

#### Conservation actions

Conservation action type: In Place

Conservation actions: 1.1. Land/water protection - Site/area protection

#### Other

##### Use and trade

Use type: International

##### Ecosystem services

Ecosystem service type: Very important

##### Research needed

Research needed: 3.1. Monitoring - Population trends

Justification for research needed: Monitoring of population trends should be conducted to confirm species status.

#### Use and trade

Use type: International

#### Ecosystem services

Ecosystem service type: Very important

#### Research needed

Research needed: 3.1. Monitoring - Population trends

Justification for research needed: Monitoring of population trends should be conducted to confirm species status.

#### Viability analysis

### Pholcus silvai

#### Species information

Scientific name: Pholcus silvai

Species authority: Wunderlich, 1995

Kingdom: Animalia

Phylum: Arthropoda

Class: Arachnida

Order: Araneae

Family: Pholcidae

Region for assessment: Global

#### Geographic range

Biogeographic realm: Palearctic

Countries: Portugal

Map of records (Google Earth): Suppl. material 43

Basis of EOO and AOO: Species Distribution Model

Basis (narrative): Multiple collection sites are recorded for the species, from both laurisilva forest and open areas above 450 m altitude (Wunderlich 1995). It was possible to perform species distribution modeling to predict its potential range with confidence limits. See methods for details.

Min Elevation/Depth (m): 450

Max Elevation/Depth (m): 1800

Range description: *Pholcus silvai* is known on the western side of Madeira Island, from both laurisilva forest and open areas above 450 m altitude.

#### New occurrences

#### Extent of occurrence

EOO (km2): 38-440-888

Trend: Stable

Justification for trend: The preferred habitats of the species, humid laurisilva forest and open areas on the western side of the island, are not experiencing any decline in area and the invasive species present should not affect the spider populations.

Causes ceased?: Yes

Causes understood?: Yes

Causes reversible?: Yes

Extreme fluctuations?: No

#### Area of occupancy

Trend: Stable

Justification for trend: The preferred habitats of the species, humid laurisilva forest and open areas on the western side of the island, are not experiencing any decline in area and the invasive species present should not affect the spider populations.

Causes ceased?: Yes

Causes understood?: Yes

Causes reversible?: Yes

Extreme fluctuations?: No

AOO (km2): 20-440-888

#### Locations

Number of locations: 0

Justification for number of locations: No known threats to the species.

Trend: Stable

Extreme fluctuations?: No

#### Population

Number of individuals: Unknown

Trend: Stable

Justification for trend: The preferred habitats of the species, humid laurisilva forest and open areas on the western side of the island, are not experiencing any decline in area and the invasive species present should not affect the spider populations.

Causes ceased?: Yes

Causes understood?: Yes

Causes reversible?: Yes

Extreme fluctuations?: No

Population Information (Narrative): No population size estimates exist.

#### Subpopulations

Trend: Stable

Extreme fluctuations?: No

Severe fragmentation?: No

#### Habitat

System: Terrestrial

Habitat specialist: No

Habitat (narrative): The species occurs in both laurisilva forest and open areas on the western side of the island.

Trend in extent, area or quality?: Stable

Justification for trend: The preferred habitats of the species, humid laurisilva forest and open areas on the western side of the island, are not experiencing any decline in area and the invasive species present should not affect the spider populations.

##### Habitat

Habitat importance: Major Importance

Habitats: 1.9. Forest - Subtropical/Tropical Moist Montane3.4. Shrubland - Temperate4.7. Grassland - Subtropical/High Altitude

#### Habitat

Habitat importance: Major Importance

Habitats: 1.9. Forest - Subtropical/Tropical Moist Montane3.4. Shrubland - Temperate4.7. Grassland - Subtropical/High Altitude

#### Ecology

Size: 4-5 mm

Generation length (yr): 1

Dependency of single sp?: No

Ecology and traits (narrative): The ecology of this species is unknown but almost certainly a tangle-web builder feeding on small arthropods.

#### Threats

Justification for threats: Unknown threats.

##### Threats

Threat type: Past

Threats: 12. Other options - Other threat

#### Threats

Threat type: Past

Threats: 12. Other options - Other threat

#### Conservation

Justification for conservation actions: Most of the species range is inside the Madeira Natural Park.

##### Conservation actions

Conservation action type: In Place

Conservation actions: 1.1. Land/water protection - Site/area protection

#### Conservation actions

Conservation action type: In Place

Conservation actions: 1.1. Land/water protection - Site/area protection

#### Other

##### Use and trade

Use type: International

##### Ecosystem services

Ecosystem service type: Very important

##### Research needed

Research needed: 3.1. Monitoring - Population trends

Justification for research needed: Monitoring of population trends should be conducted to confirm this species' status.

#### Use and trade

Use type: International

#### Ecosystem services

Ecosystem service type: Very important

#### Research needed

Research needed: 3.1. Monitoring - Population trends

Justification for research needed: Monitoring of population trends should be conducted to confirm this species' status.

#### Viability analysis

### Prinerigone pigra

#### Species information

Scientific name: Prinerigone pigra

Species authority: (Blackwall, 1862)

Kingdom: Animalia

Phylum: Arthropoda

Class: Arachnida

Order: Araneae

Family: Linyphiidae

Region for assessment: Global

#### Geographic range

Biogeographic realm: Palearctic

Countries: Portugal

Map of records (Google Earth): Suppl. material 44

Basis of EOO and AOO: Species Distribution Model

Basis (narrative): Only three collection sites have ever been recorded for this species, mostly from laurisilva forest (Blackwall 1862, Schenkel 1938, Wunderlich 1995). It was possible to perform species distribution modeling to predict its potential range with confidence limits, although it must be carefully considered due to the low number of samples. See methods for details.

Min Elevation/Depth (m): 600

Max Elevation/Depth (m): 1850

Range description: *Prinerigone pigra* seems to be restricted to high-altitude forest (above 600 m) in Madeira Island.

#### New occurrences

#### Extent of occurrence

EOO (km2): 52-456-87

Trend: Unknown

Justification for trend: There are no recent collections of the species. It does not seem to be common given the scarce number of records. Impossible to infer on the current trend.

Causes ceased?: Unknown

Causes understood?: Unknown

Causes reversible?: Unknown

Extreme fluctuations?: No

#### Area of occupancy

Trend: Unknown

Justification for trend: There are no recent collections of the species. It does not seem to be common given the scarce number of records. Impossible to infer on the current trend.

Causes ceased?: Unknown

Causes understood?: Unknown

Causes reversible?: Unknown

Extreme fluctuations?: No

AOO (km2): 16-456-872

#### Locations

Number of locations: Unknown

Justification for number of locations: Impossible to infer if there are any threats.

Trend: Unknown

Extreme fluctuations?: No

#### Population

Number of individuals: Unknown

Trend: Unknown

Justification for trend: There are no recent collections of the species. It does not seem to be common given the scarce number of records. Impossible to infer on the current trend.

Causes ceased?: Unknown

Causes understood?: Unknown

Causes reversible?: Unknown

Extreme fluctuations?: No

Population Information (Narrative): No population size estimates exist.

#### Subpopulations

Trend: Unknown

Extreme fluctuations?: No

Severe fragmentation?: Unknown

#### Habitat

System: Terrestrial

Habitat specialist: Unknown

Habitat (narrative): It seems to be mostly found in high-altitude laurisilva forest but possibly also in other habitats above 600m.

Trend in extent, area or quality?: Unknown

Justification for trend: There are no recent collections of the species. It does not seem to be common given the scarce number of records. Therefore, it is impossible to infer the current trend.

##### Habitat

Habitat importance: Major Importance

Habitats: 1.9. Forest - Subtropical/Tropical Moist Montane18. Unknown

#### Habitat

Habitat importance: Major Importance

Habitats: 1.9. Forest - Subtropical/Tropical Moist Montane18. Unknown

#### Ecology

Size: 2 mm

Generation length (yr): 1

Dependency of single sp?: No

Ecology and traits (narrative): Unknown, but sister taxa such as *Prinerigone vagans* (Audouin, 1826) are sheet-web weavers at ground and low vegetation levels.

#### Threats

Justification for threats: Unknown threats.

##### Threats

Threat type: Past

Threats: 12. Other options - Other threat

#### Threats

Threat type: Past

Threats: 12. Other options - Other threat

#### Conservation

Justification for conservation actions: Part of the species range is inside the Madeira Natural Park.

##### Conservation actions

Conservation action type: In Place

Conservation actions: 1.1. Land/water protection - Site/area protection

#### Conservation actions

Conservation action type: In Place

Conservation actions: 1.1. Land/water protection - Site/area protection

#### Other

##### Use and trade

Use type: International

##### Ecosystem services

Ecosystem service type: Very important

##### Research needed

Research needed: 1.2. Research - Population size, distribution & trends1.3. Research - Life history & ecology1.5. Research - Threats

Justification for research needed: Basic information is needed on its distribution, ecology and possible threats.

#### Use and trade

Use type: International

#### Ecosystem services

Ecosystem service type: Very important

#### Research needed

Research needed: 1.2. Research - Population size, distribution & trends1.3. Research - Life history & ecology1.5. Research - Threats

Justification for research needed: Basic information is needed on its distribution, ecology and possible threats.

#### Viability analysis

### Rugathodes madeirensis

#### Species information

Scientific name: Rugathodes madeirensis

Species authority: Wunderlich, 1987

Kingdom: Animalia

Phylum: Arthropoda

Class: Arachnida

Order: Araneae

Family: Theridiidae

Region for assessment: Global

#### Geographic range

Biogeographic realm: Palearctic

Countries: Portugal

Map of records (Google Earth): Suppl. material 45

Basis of EOO and AOO: Species Distribution Model

Basis (narrative): Multiple collection sites are recorded for the species, mostly recent and in laurisilva forest (Schenkel 1938, Denis 1962, Wunderlich 1987, Wunderlich 1992, Crespo et al. 2014b). It was possible to perform species distribution modeling to predict its potential range with confidence limits. See methods for details.

Min Elevation/Depth (m): 50

Max Elevation/Depth (m): 1700

Range description: *Rugathodes madeirensis* is one of the most common species in Madeira Island, known throughout the laurisilva forest and also other habitats including pine plantations and close to urban areas.

#### New occurrences

#### Extent of occurrence

EOO (km2): 235-432-792

Trend: Stable

Justification for trend: The species seems to be able to live within several habitat types, even close to human settlements.

Causes ceased?: Yes

Causes understood?: Yes

Causes reversible?: Yes

Extreme fluctuations?: No

#### Area of occupancy

Trend: Stable

Justification for trend: The species seems to be able to live on several habitat types, even close to human settlements.

Causes ceased?: Yes

Causes understood?: Yes

Causes reversible?: Yes

Extreme fluctuations?: No

AOO (km2): 188-404-792

#### Locations

Number of locations: 0

Justification for number of locations: No known threats to the species.

Trend: Stable

Extreme fluctuations?: No

#### Population

Number of individuals: Unknown

Trend: Stable

Justification for trend: The species seems to be able to live within several habitat types, even close to human settlements.

Causes ceased?: Yes

Causes understood?: Yes

Causes reversible?: Yes

Extreme fluctuations?: No

Population Information (Narrative): No population size estimates exist.

#### Subpopulations

Trend: Stable

Extreme fluctuations?: No

Severe fragmentation?: No

#### Habitat

System: Terrestrial

Habitat specialist: No

Habitat (narrative): *Rugathodes madeirensis* is one of the most common species in Madeira Island, known throughout the laurisilva forest and also other habitats including pine plantations and close to urban areas.

Trend in extent, area or quality?: Stable

##### Habitat

Habitat importance: Major Importance

Habitats: 1.9. Forest - Subtropical/Tropical Moist Montane

##### Habitat

Habitat importance: Suitable

Habitats: 14.3. Artificial/Terrestrial - Plantations14.4. Artificial/Terrestrial - Rural Gardens

#### Habitat

Habitat importance: Major Importance

Habitats: 1.9. Forest - Subtropical/Tropical Moist Montane

#### Habitat

Habitat importance: Suitable

Habitats: 14.3. Artificial/Terrestrial - Plantations14.4. Artificial/Terrestrial - Rural Gardens

#### Ecology

Size: 2 mm

Generation length (yr): 1

Dependency of single sp?: No

Ecology and traits (narrative): Cobweb spider at all vegetation layers feeding on small arthropods.

#### Threats

Justification for threats: Unknown threats.

##### Threats

Threat type: Past

Threats: 12. Other options - Other threat

#### Threats

Threat type: Past

Threats: 12. Other options - Other threat

#### Conservation

Justification for conservation actions: Most of the species range is inside the Madeira Natural Park.

##### Conservation actions

Conservation action type: In Place

Conservation actions: 1.1. Land/water protection - Site/area protection

#### Conservation actions

Conservation action type: In Place

Conservation actions: 1.1. Land/water protection - Site/area protection

#### Other

##### Use and trade

Use type: International

##### Ecosystem services

Ecosystem service type: Very important

##### Research needed

Research needed: 3.1. Monitoring - Population trends

Justification for research needed: Monitoring of population trends should be conducted to confirm species status.

#### Use and trade

Use type: International

#### Ecosystem services

Ecosystem service type: Very important

#### Research needed

Research needed: 3.1. Monitoring - Population trends

Justification for research needed: Monitoring of population trends should be conducted to confirm species status.

#### Viability analysis

### Scotognapha paivani

#### Species information

Scientific name: Scotognapha paivani

Species authority: (Blackwall, 1864)

Kingdom: Animalia

Phylum: Arthropoda

Class: Arachnida

Order: Araneae

Family: Gnaphosidae

Region for assessment: Global

#### Geographic range

Biogeographic realm: Palearctic

Countries: Portugal

Map of records (Google Earth): Suppl. material 46

Basis of EOO and AOO: Observed

Basis (narrative): As the species is thought to be restricted to the three small islands/islets of Selvagens, the EOO and AOO can be calculated with some confidence.

Min Elevation/Depth (m): 0

Max Elevation/Depth (m): 160

Range description: Restricted to Selvagem Grande, Selvagem Pequena and Ilhéu de Fora in the archipelago of Selvagens.

#### New occurrences

#### Extent of occurrence

EOO (km2): 13

Trend: Stable

Justification for trend: The EOO seems to be stable with no signs of loss or known threats.

Causes ceased?: Yes

Causes understood?: Yes

Causes reversible?: Yes

Extreme fluctuations?: No

#### Area of occupancy

Trend: Stable

Justification for trend: The AOO seems to be stable with no signs of loss or known threats.

Causes ceased?: Yes

Causes understood?: Yes

Causes reversible?: Yes

Extreme fluctuations?: No

AOO (km2): 12

#### Locations

Number of locations: 0

Justification for number of locations: No known threats to the species.

Trend: Stable

Extreme fluctuations?: No

#### Population

Number of individuals: Unknown

Trend: Stable

Justification for trend: The population size seems to be stable with no signs of loss or known threats.

Causes ceased?: Yes

Causes understood?: Yes

Causes reversible?: Yes

Extreme fluctuations?: No

Population Information (Narrative): No population size estimates exist.

#### Subpopulations

Number of subpopulations: 3

Trend: Stable

Extreme fluctuations?: No

Severe fragmentation?: No

#### Habitat

System: Terrestrial

Habitat specialist: Unknown

Habitat (narrative): The islands and islets of Desertas have a mix of grassland and rocky outcrops within coastal cliffs. The species seems to be relatively common in grassland, unknown if also occurs in other habitats.

Trend in extent, area or quality?: Stable

Justification for trend: The habitat in Selvagem Grande was subject of a recovery plan during 2000-2002 and seems to be stable with no signs of loss or known threats.

##### Habitat

Habitat importance: Major Importance

Habitats: 4.5. Grassland - Subtropical/Tropical Dry

#### Habitat

Habitat importance: Major Importance

Habitats: 4.5. Grassland - Subtropical/Tropical Dry

#### Ecology

Size: 3-10 mm

Generation length (yr): 1

Dependency of single sp?: No

Ecology and traits (narrative): This species is probably a nocturnal hunter of small arthropods at ground level.

#### Threats

Justification for threats: Unknown threats.

##### Threats

Threat type: Past

Threats: 12. Other options - Other threat

#### Threats

Threat type: Past

Threats: 12. Other options - Other threat

#### Conservation

Justification for conservation actions: The species range is inside the Selvagens Nature Reserve.

##### Conservation actions

Conservation action type: In Place

Conservation actions: 1.1. Land/water protection - Site/area protection

#### Conservation actions

Conservation action type: In Place

Conservation actions: 1.1. Land/water protection - Site/area protection

#### Other

##### Use and trade

Use type: International

##### Ecosystem services

Ecosystem service type: Very important

##### Research needed

Research needed: 3.1. Monitoring - Population trends

Justification for research needed: Monitoring of population trends should be conducted to confirm species status.

#### Use and trade

Use type: International

#### Ecosystem services

Ecosystem service type: Very important

#### Research needed

Research needed: 3.1. Monitoring - Population trends

Justification for research needed: Monitoring of population trends should be conducted to confirm species status.

#### Viability analysis

### Spermophorides selvagensis

#### Species information

Scientific name: Spermophorides selvagensis

Species authority: Wunderlich, 1992

Kingdom: Animalia

Phylum: Arthropoda

Class: Arachnida

Order: Araneae

Family: Pholcidae

Region for assessment: Global

#### Geographic range

Biogeographic realm: Palearctic

Countries: Portugal

Map of records (Google Earth): Suppl. material 47

Basis of EOO and AOO: Observed

Basis (narrative): This species is only known from Selvagem Grande (Denis 1963, Wunderlich 1992, Crespo et al. 2009b), being possible to calculate the EOO and AOO with reasonable confidence.

Min Elevation/Depth (m): 0

Max Elevation/Depth (m): 160

Range description: Only known from Selvagem Grande, from both a small coastal cave (Gruta das Pardelas) and rocky areas.

#### New occurrences

#### Extent of occurrence

EOO (km2): 4

Trend: Stable

Justification for trend: The EOO seems to be stable with no signs of loss or known threats.

Causes ceased?: Yes

Causes understood?: Yes

Causes reversible?: Yes

Extreme fluctuations?: No

#### Area of occupancy

Trend: Stable

Justification for trend: The AOO seems to be stable with no signs of loss or known threats.

Causes ceased?: Yes

Causes understood?: Yes

Causes reversible?: Yes

Extreme fluctuations?: No

AOO (km2): 4

#### Locations

Number of locations: 0

Justification for number of locations: No known threats to the species.

Trend: Stable

Extreme fluctuations?: No

#### Population

Number of individuals: Unknown

Trend: Stable

Justification for trend: The population size seems to be stable with no signs of loss or known threats.

Causes ceased?: Yes

Causes understood?: Yes

Causes reversible?: Yes

Extreme fluctuations?: No

Population Information (Narrative): No population size estimates exist.

#### Subpopulations

Number of subpopulations: 1

Trend: Stable

Extreme fluctuations?: No

Severe fragmentation?: No

#### Habitat

System: Terrestrial

Habitat specialist: No

Habitat (narrative): Found both in a coastal cave and rocky outcrops.

Trend in extent, area or quality?: Stable

##### Habitat

Habitat importance: Major Importance

Habitats: 6. Rocky areas (e.g. inland cliffs, mountain peaks)13.1. Marine Coastal/Supratidal - Sea Cliffs and Rocky Offshore Islands13.2. Marine Coastal/Supratidal - Coastal Caves/Karst

#### Habitat

Habitat importance: Major Importance

Habitats: 6. Rocky areas (e.g. inland cliffs, mountain peaks)13.1. Marine Coastal/Supratidal - Sea Cliffs and Rocky Offshore Islands13.2. Marine Coastal/Supratidal - Coastal Caves/Karst

#### Ecology

Size: 1-2 mm

Generation length (yr): 1

Dependency of single sp?: No

Ecology and traits (narrative): This is a tangle-web spider hunting for small arthropods on rock walls.

#### Threats

Justification for threats: Unknown threats.

##### Threats

Threat type: Past

Threats: 12. Other options - Other threat

#### Threats

Threat type: Past

Threats: 12. Other options - Other threat

#### Conservation

Justification for conservation actions: The species range is inside the Selvagens Nature Reserve.

##### Conservation actions

Conservation action type: In Place

Conservation actions: 1.1. Land/water protection - Site/area protection

#### Conservation actions

Conservation action type: In Place

Conservation actions: 1.1. Land/water protection - Site/area protection

#### Other

##### Use and trade

Use type: International

##### Ecosystem services

Ecosystem service type: Very important

##### Research needed

Research needed: 3.1. Monitoring - Population trends

Justification for research needed: Monitoring of population trends should be conducted to confirm species status.

#### Use and trade

Use type: International

#### Ecosystem services

Ecosystem service type: Very important

#### Research needed

Research needed: 3.1. Monitoring - Population trends

Justification for research needed: Monitoring of population trends should be conducted to confirm species status.

#### Viability analysis

### Steatoda distincta

#### Species information

Scientific name: Steatoda distincta

Species authority: (Blackwall, 1859)

Kingdom: Animalia

Phylum: Arthropoda

Class: Arachnida

Order: Araneae

Family: Theridiidae

Taxonomic notes: Possible junior synonym of *Steatoda paykulliana* (Walckenaer, 1806), a widespread palearctic species (Denis 1962).

Region for assessment: Global

#### Geographic range

Biogeographic realm: Palearctic

Countries: Portugal

Map of records (Google Earth): Suppl. material 48

Basis of EOO and AOO: Unknown

Basis (narrative): The EOO and AOO of this species are unknown.

Range description: Only mentioned for "Madeira" (Blackwall 1859, Warburton 1892), with no locality data.

#### New occurrences

#### Extent of occurrence

EOO (km2): Unknown

Trend: Unknown

Causes ceased?: Unknown

Causes understood?: Unknown

Causes reversible?: Unknown

Extreme fluctuations?: Unknown

#### Area of occupancy

Trend: Unknown

Causes ceased?: Unknown

Causes understood?: Unknown

Causes reversible?: Unknown

Extreme fluctuations?: Unknown

AOO (km2): Unknown

#### Locations

Number of locations: Unknown

Trend: Unknown

Extreme fluctuations?: Unknown

#### Population

Number of individuals: Unknown

Trend: Unknown

Causes ceased?: Unknown

Causes understood?: Unknown

Causes reversible?: Unknown

Extreme fluctuations?: Unknown

Population Information (Narrative): No population size estimates exist.

#### Subpopulations

Trend: Unknown

Extreme fluctuations?: Unknown

Severe fragmentation?: Unknown

#### Habitat

System: Terrestrial

Habitat specialist: Unknown

Habitat (narrative): The species habitat is unknown.

Trend in extent, area or quality?: Unknown

##### Habitat

Habitat importance: Major Importance

Habitats: 18. Unknown

#### Habitat

Habitat importance: Major Importance

Habitats: 18. Unknown

#### Ecology

Size: 5 mm

Generation length (yr): 1

Dependency of single sp?: No

Ecology and traits (narrative): Not much is known about this species ecology except it should be a cobweb builder (if the species is valid).

#### Threats

Justification for threats: Unknown threats.

##### Threats

Threat type: Past

Threats: 12. Other options - Other threat

#### Threats

Threat type: Past

Threats: 12. Other options - Other threat

#### Conservation

##### Conservation actions

#### Conservation actions

#### Other

##### Use and trade

Use type: International

##### Ecosystem services

Ecosystem service type: Very important

##### Research needed

Research needed: 1.1. Research - Taxonomy1.2. Research - Population size, distribution & trends1.3. Research - Life history & ecology1.5. Research - Threats

Justification for research needed: *S. distincta* probably is a synonym of a widespread species (Denis 1962) and needs, first of all, taxonomic clarification. If valid, basic information would be needed on its distribution, ecology and possible threats.

#### Use and trade

Use type: International

#### Ecosystem services

Ecosystem service type: Very important

#### Research needed

Research needed: 1.1. Research - Taxonomy1.2. Research - Population size, distribution & trends1.3. Research - Life history & ecology1.5. Research - Threats

Justification for research needed: *S. distincta* probably is a synonym of a widespread species (Denis 1962) and needs, first of all, taxonomic clarification. If valid, basic information would be needed on its distribution, ecology and possible threats.

#### Viability analysis

### Tenuiphantes tenebricoloides

#### Species information

Scientific name: Tenuiphantes tenebricoloides

Species authority: (Schenkel, 1938)

Kingdom: Animalia

Phylum: Arthropoda

Class: Arachnida

Order: Araneae

Family: Linyphiidae

Region for assessment: Global

#### Geographic range

Biogeographic realm: Palearctic

Countries: Portugal

Map of records (Google Earth): Suppl. material 49

Basis of EOO and AOO: Species Distribution Model

Basis (narrative): Multiple collection sites are recorded for the species, mostly recent and in laurisilva forest (Schenkel 1938, Denis 1962, Wunderlich 1987, unpublished). It was possible to perform species distribution modeling to predict its potential range with confidence limits. See methods for details.

Min Elevation/Depth (m): 550

Max Elevation/Depth (m): 1750

Range description: *Tenuiphantes tenebricoloides* is one of the most common species in Madeira Island, known throughout the laurisilva forest and also other habitats including planted/cultivated forest and close to urban areas.

#### New occurrences

#### Extent of occurrence

EOO (km2): 181-488-700

Trend: Stable

Justification for trend: Although most common in laurisilva forest, the species seems to be able to live on several habitat types, even close to human settlements.

Causes ceased?: Yes

Causes understood?: Yes

Causes reversible?: Yes

Extreme fluctuations?: No

#### Area of occupancy

Trend: Stable

Justification for trend: Although most common in laurisilva forest, the species seems to be able to live on several habitat types, even close to human settlements.

Causes ceased?: Yes

Causes understood?: Yes

Causes reversible?: Yes

Extreme fluctuations?: No

AOO (km2): 160-488-692

#### Locations

Number of locations: 0

Justification for number of locations: No known threats to the species.

Trend: Stable

Extreme fluctuations?: No

#### Population

Number of individuals: Unknown

Trend: Stable

Justification for trend: Although most common in laurisilva forest, the species seems to be able to live on several habitat types, even close to human settlements.

Causes ceased?: Yes

Causes understood?: Yes

Causes reversible?: Yes

Extreme fluctuations?: No

Population Information (Narrative): No population size estimates exist.

#### Subpopulations

Trend: Stable

Extreme fluctuations?: No

Severe fragmentation?: No

#### Habitat

System: Terrestrial

Habitat specialist: No

Habitat (narrative): Most common in humid laurissilva forest but recorded from several habitat types.

Trend in extent, area or quality?: Stable

Justification for trend: Although most common in laurisilva forest, the species seems to be able to live on several habitat types such as cultivated forest, even close to human settlements.

##### Habitat

Habitat importance: Major Importance

Habitats: 1.9. Forest - Subtropical/Tropical Moist Montane

##### Habitat

Habitat importance: Suitable

Habitats: 14.3. Artificial/Terrestrial - Plantations14.4. Artificial/Terrestrial - Rural Gardens

#### Habitat

Habitat importance: Major Importance

Habitats: 1.9. Forest - Subtropical/Tropical Moist Montane

#### Habitat

Habitat importance: Suitable

Habitats: 14.3. Artificial/Terrestrial - Plantations14.4. Artificial/Terrestrial - Rural Gardens

#### Ecology

Size: 2-3 mm

Generation length (yr): 1

Dependency of single sp?: No

Ecology and traits (narrative): This species is a sheet-web builder at the arboreal layer feeding mainly on small insects.

#### Threats

Justification for threats: Unknown threats.

##### Threats

Threat type: Past

Threats: 12. Other options - Other threat

#### Threats

Threat type: Past

Threats: 12. Other options - Other threat

#### Conservation

Justification for conservation actions: Most of the species range is inside the Madeira Natural Park.

##### Conservation actions

Conservation action type: In Place

Conservation actions: 1.1. Land/water protection - Site/area protection

#### Conservation actions

Conservation action type: In Place

Conservation actions: 1.1. Land/water protection - Site/area protection

#### Other

##### Use and trade

Use type: International

##### Ecosystem services

Ecosystem service type: Very important

##### Research needed

Research needed: 3.1. Monitoring - Population trends

Justification for research needed: Monitoring of population trends should be conducted to confirm species status.

#### Use and trade

Use type: International

#### Ecosystem services

Ecosystem service type: Very important

#### Research needed

Research needed: 3.1. Monitoring - Population trends

Justification for research needed: Monitoring of population trends should be conducted to confirm species status.

#### Viability analysis

### Trogloneta madeirensis

#### Species information

Scientific name: Trogloneta madeirensis

Species authority: Wunderlich, 1987

Kingdom: Animalia

Phylum: Arthropoda

Class: Arachnida

Order: Araneae

Family: Mysmenidae

Region for assessment: Global

#### Geographic range

Biogeographic realm: Palearctic

Countries: Portugal

Map of records (Google Earth): Suppl. material 50

Basis of EOO and AOO: Species Distribution Model

Basis (narrative): Multiple collection sites are recorded for the species, mostly recent and in laurisilva forest (Wunderlich 1987, Wunderlich 1992, Crespo et al. 2014b). It was possible to perform species distribution modeling to predict its potential range with confidence limits. See methods for details.

Range description: *Trogloneta madeirensis* is one of the most common species in Madeira Island, known throughout the laurisilva forest and also other habitats including caves and close to urban areas.

#### New occurrences

#### Extent of occurrence

EOO (km2): 202-389-764

Trend: Stable

Justification for trend: Although most common in laurisilva forest, the species seems to be able to live on several habitat types, even close to human settlements.

Causes ceased?: Yes

Causes understood?: Yes

Causes reversible?: Yes

Extreme fluctuations?: No

#### Area of occupancy

Trend: Stable

Justification for trend: Although most common in laurisilva forest, the species seems to be able to live on several habitat types, even close to human settlements.

Causes ceased?: Yes

Causes understood?: Yes

Causes reversible?: Yes

Extreme fluctuations?: No

AOO (km2): 172-368-764

#### Locations

Number of locations: 0

Justification for number of locations: No known threats to the species.

Trend: Stable

Extreme fluctuations?: No

#### Population

Number of individuals: Unknown

Trend: Stable

Justification for trend: Although most common in laurisilva forest, the species seems to be able to live on several habitat types, even close to human settlements.

Causes ceased?: Yes

Causes understood?: Yes

Causes reversible?: Yes

Extreme fluctuations?: No

Population Information (Narrative): No population size estimates exist.

#### Subpopulations

Trend: Stable

Extreme fluctuations?: No

Severe fragmentation?: No

#### Habitat

System: Terrestrial

Habitat specialist: No

Habitat (narrative): Most common in humid laurissilva forest but recorded from several habitat types.

Trend in extent, area or quality?: Decline (observed)

Justification for trend: Although most common in laurisilva forest, the species seems to be able to live on several habitat types, even close to human settlements.

##### Habitat

Habitat importance: Major Importance

Habitats: 1.9. Forest - Subtropical/Tropical Moist Montane

##### Habitat

Habitat importance: Suitable

Habitats: 7.1. Caves and Subterranean Habitats (non-aquatic) - Caves14.4. Artificial/Terrestrial - Rural Gardens

#### Habitat

Habitat importance: Major Importance

Habitats: 1.9. Forest - Subtropical/Tropical Moist Montane

#### Habitat

Habitat importance: Suitable

Habitats: 7.1. Caves and Subterranean Habitats (non-aquatic) - Caves14.4. Artificial/Terrestrial - Rural Gardens

#### Ecology

Size: 0.9-1.3 mm

Generation length (yr): 1

Dependency of single sp?: No

Ecology and traits (narrative): This tiny spider has been found living among the rocks and trees, shrubs and herbs and also in moss on tree trunks (Wunderlich 1987). It possibly builds three-dimensional webs (Hajer 2000).

#### Threats

Justification for threats: Unknown threats.

##### Threats

Threat type: Past

Threats: 12. Other options - Other threat

#### Threats

Threat type: Past

Threats: 12. Other options - Other threat

#### Conservation

Justification for conservation actions: Most of the species range is inside the Madeira Natural Park.

##### Conservation actions

Conservation action type: In Place

Conservation actions: 1.1. Land/water protection - Site/area protection

#### Conservation actions

Conservation action type: In Place

Conservation actions: 1.1. Land/water protection - Site/area protection

#### Other

##### Use and trade

Use type: International

##### Ecosystem services

Ecosystem service type: Very important

##### Research needed

Research needed: 3.1. Monitoring - Population trends

Justification for research needed: Monitoring of population trends should be conducted to confirm species status.

#### Use and trade

Use type: International

#### Ecosystem services

Ecosystem service type: Very important

#### Research needed

Research needed: 3.1. Monitoring - Population trends

Justification for research needed: Monitoring of population trends should be conducted to confirm species status.

#### Viability analysis

### Turinyphia maderiana

#### Species information

Scientific name: Turinyphia maderiana

Species authority: (Schenkel, 1938)

Kingdom: Animalia

Phylum: Arthropoda

Class: Arachnida

Order: Araneae

Family: Linyphiidae

Region for assessment: Global

#### Geographic range

Biogeographic realm: Palearctic

Countries: Portugal

Map of records (Google Earth): Suppl. material 51

Basis of EOO and AOO: Species Distribution Model

Basis (narrative): Multiple collection sites are recorded for the species, mostly recent and in laurisilva forest (Schenkel 1938, Denis 1962, Wunderlich 1987, Crespo et al. 2014b). It was possible to perform species distribution modeling to predict its potential range with confidence limits. See methods for details.

Min Elevation/Depth (m): 300

Max Elevation/Depth (m): 1700

Range description: *Turinyphia maderiana* is known throughout the laurisilva forest that occupies about 20% of the island, mainly on its steep and humid northern slopes.

#### New occurrences

#### Extent of occurrence

EOO (km2): 181-351-700

Trend: Stable

Justification for trend: The preferred habitat of the species, humid laurisilva forest, is not experiencing any decline in area and the invasive species present should not affect the spider populations.

Causes ceased?: Yes

Causes understood?: Yes

Causes reversible?: Yes

Extreme fluctuations?: No

#### Area of occupancy

Trend: Stable

Justification for trend: The preferred habitat of the species, humid laurisilva forest, is not experiencing any decline in area and the invasive species present should not affect the spider populations.

Causes ceased?: Yes

Causes understood?: Yes

Causes reversible?: Yes

Extreme fluctuations?: No

AOO (km2): 172-344-700

#### Locations

Number of locations: 0

Justification for number of locations: No known threats to the species.

Trend: Stable

Extreme fluctuations?: No

#### Population

Number of individuals: Unknown

Trend: Stable

Justification for trend: The preferred habitat of the species, humid laurisilva forest, is not experiencing any decline in area and the invasive species present should not affect the spider populations.

Causes ceased?: Yes

Causes understood?: Yes

Causes reversible?: Yes

Extreme fluctuations?: No

Population Information (Narrative): No population size estimates exist.

#### Subpopulations

Trend: Stable

Extreme fluctuations?: No

Severe fragmentation?: No

#### Habitat

System: Terrestrial

Habitat specialist: Yes

Habitat (narrative): Humid laurisilva forest on the northern slopes of Madeira Island.

Trend in extent, area or quality?: Stable

Justification for trend: The preferred habitat of the species, humid laurisilva forest, is not experiencing any decline in area and the invasive species present should not affect the spider populations.

##### Habitat

Habitat importance: Major Importance

Habitats: 1.9. Forest - Subtropical/Tropical Moist Montane

#### Habitat

Habitat importance: Major Importance

Habitats: 1.9. Forest - Subtropical/Tropical Moist Montane

#### Ecology

Size: 2 mm

Generation length (yr): 1

Dependency of single sp?: No

Ecology and traits (narrative): This species is a sheet-web builder at different vegetation levels feeding mainly on small insects.

#### Threats

Justification for threats: Unknown threats.

##### Threats

Threat type: Past

Threats: 12. Other options - Other threat

#### Threats

Threat type: Past

Threats: 12. Other options - Other threat

#### Conservation

Justification for conservation actions: All of the species range is inside the Madeira Natural Park.

##### Conservation actions

Conservation action type: In Place

Conservation actions: 1.1. Land/water protection - Site/area protection

#### Conservation actions

Conservation action type: In Place

Conservation actions: 1.1. Land/water protection - Site/area protection

#### Other

##### Use and trade

Use type: International

##### Ecosystem services

Ecosystem service type: Very important

##### Research needed

Research needed: 3.1. Monitoring - Population trends

Justification for research needed: Monitoring of population trends should be conducted to confirm species status.

#### Use and trade

Use type: International

#### Ecosystem services

Ecosystem service type: Very important

#### Research needed

Research needed: 3.1. Monitoring - Population trends

Justification for research needed: Monitoring of population trends should be conducted to confirm species status.

#### Viability analysis

### Typhochrestus madeirensis

#### Species information

Scientific name: Typhochrestus madeirensis

Species authority: Crespo, 2013

Kingdom: Animalia

Phylum: Arthropoda

Class: Arachnida

Order: Araneae

Family: Linyphiidae

Region for assessment: Global

#### Geographic range

Biogeographic realm: Palearctic

Countries: Portugal

Map of records (Google Earth): Suppl. material 52

Basis of EOO and AOO: Species Distribution Model

Basis (narrative): Multiple collection sites are recorded for the species, all recent and in open habitats (Crespo et al. 2013, Crespo et al. 2014b). It was possible to perform species distribution modeling to predict its potential range with confidence limits. See methods for details.

Min Elevation/Depth (m): 300

Max Elevation/Depth (m): 1750

Range description: Living on relatively high-altitude open areas across Madeira (Paúl da Serra and the region between the highest mountain peaks), Deserta Grande (south plateau) and Bugio (south and north plateaus).

#### New occurrences

#### Extent of occurrence

EOO (km2): 372-568-1397

Trend: Stable

Justification for trend: The species seems to be able to live on open habitat patches across different islands.

Causes ceased?: Yes

Causes understood?: Yes

Causes reversible?: Yes

Extreme fluctuations?: No

#### Area of occupancy

Trend: Stable

Justification for trend: The species seems to be able to live on open habitat patches across different islands.

Causes ceased?: Yes

Causes understood?: Yes

Causes reversible?: Yes

Extreme fluctuations?: No

AOO (km2): 20-64-656

#### Locations

Number of locations: 0

Justification for number of locations: No known threats to the species.

Trend: Stable

Extreme fluctuations?: No

#### Population

Number of individuals: Unknown

Trend: Stable

Justification for trend: The species seems to be able to live on open habitat patches across different islands.

Causes ceased?: Yes

Causes understood?: Yes

Causes reversible?: Yes

Extreme fluctuations?: No

Population Information (Narrative): No population size estimates exist.

#### Subpopulations

Trend: Stable

Extreme fluctuations?: No

Severe fragmentation?: No

#### Habitat

System: Terrestrial

Habitat specialist: No

Habitat (narrative): The species seems to be able to live within different open habitat types, including grassland and rocky mountain peaks.

Trend in extent, area or quality?: Stable

Justification for trend: The species seems to be able to live on open habitat patches across different islands.

##### Habitat

Habitat importance: Major Importance

Habitats: 4.7. Grassland - Subtropical/High Altitude6. Rocky areas (e.g. inland cliffs, mountain peaks)

#### Habitat

Habitat importance: Major Importance

Habitats: 4.7. Grassland - Subtropical/High Altitude6. Rocky areas (e.g. inland cliffs, mountain peaks)

#### Ecology

Size: 1.2-1.4 mm

Generation length (yr): 1

Dependency of single sp?: No

Ecology and traits (narrative): Living at ground level, probably actively hunting for small insects.

#### Threats

Justification for threats: Unknown threats.

##### Threats

Threat type: Past

Threats: 12. Other options - Other threat

#### Threats

Threat type: Past

Threats: 12. Other options - Other threat

#### Conservation

Justification for conservation actions: Most of the known species range is inside protected areas.

##### Conservation actions

Conservation action type: In Place

Conservation actions: 1. Land/water protection

#### Conservation actions

Conservation action type: In Place

Conservation actions: 1. Land/water protection

#### Other

##### Use and trade

Use type: International

##### Ecosystem services

Ecosystem service type: Very important

##### Research needed

Research needed: 3.1. Monitoring - Population trends

Justification for research needed: Monitoring of population trends should be conducted to confirm species status.

#### Use and trade

Use type: International

#### Ecosystem services

Ecosystem service type: Very important

#### Research needed

Research needed: 3.1. Monitoring - Population trends

Justification for research needed: Monitoring of population trends should be conducted to confirm species status.

#### Viability analysis

### Xysticus grohi

#### Species information

Scientific name: Xysticus grohi

Species authority: (Wunderlich, 1992)

Kingdom: Animalia

Phylum: Arthropoda

Class: Arachnida

Order: Araneae

Family: Thomisidae

Region for assessment: Global

#### Geographic range

Biogeographic realm: Palearctic

Countries: Portugal

Map of records (Google Earth): Suppl. material 53

Basis of EOO and AOO: Observed

Basis (narrative): Species possibly restricted to the islands of Deserta Grande and Bugio (Wunderlich 1987, Wunderlich 1992, Crespo et al. 2013, unpublished). The EOO can be calculated with reasonable confidence.

Min Elevation/Depth (m): 0

Max Elevation/Depth (m): 400

Range description: This species is possibly restricted to the islands of Deserta Grande and Bugio where it is know from three sites but probably extends across the entire islands given the adequate microhabitat (steep slopes).

#### New occurrences

#### Extent of occurrence

EOO (km2): 24

Trend: Stable

Justification for trend: Although the invasive *Xysticus nubilus* Simon, 1875, first detected in 2011, seems to have occupied all the flat areas of Desertas, the steep slopes around the islands may constitute refuge to this single island endemic.

Causes ceased?: Yes

Causes understood?: Yes

Causes reversible?: Yes

Extreme fluctuations?: No

#### Area of occupancy

Trend: Stable

Justification for trend: Although the invasive *X. nubilus* seems to have occupied all the flat areas of Desertas, the steep slopes around the islands may constitute refuge to this single island endemic.

Causes ceased?: Yes

Causes understood?: Yes

Causes reversible?: Yes

Extreme fluctuations?: No

AOO (km2): 24

#### Locations

Number of locations: 1

Justification for number of locations: A single event, the introduction of the invasive species *X. nubilus*, first detected in 2011, seems to have originated the extirpation of *X. grohi* from most of the islands. This invasive process may continue in the future to the current refuge of the endemic species, the steep coastal slopes.

Trend: Stable

Extreme fluctuations?: No

#### Population

Number of individuals: Unknown

Trend: Decline (inferred)

Justification for trend: The introduction of the invasive congener *X. nubilus* seems to have originated the extirpation of *X. grohi* from most of the islands. This invasive process may continue in the future to the current refuge of the endemic species, the steep coastal slopes.

Basis for decline: (e) the effects of introduced taxa, hybridization, pathogens, pollutants, competitors or parasites.

Causes ceased?: No

Causes understood?: Yes

Causes reversible?: No

Extreme fluctuations?: No

Population Information (Narrative): No population size estimates exist. The introduction of the invasive *X. nubilus* seems to have originated the extirpation of *X. grohi* from most of the islands. This invasive process may continue in the future to the current refuge of the endemic species, the steep coastal slopes.

#### Subpopulations

Number of subpopulations: Unknown

Trend: Decline (inferred)

Justification for trend: The introduction of *X. nubilus* seems to have originated the extirpation of *X. grohi* from most of the islands.

Extreme fluctuations?: No

Severe fragmentation?: Unknown

#### Habitat

System: Terrestrial

Habitat specialist: Yes

Habitat (narrative): This species is possibly restricted to rocky areas, now only on coastal slopes.

Trend in extent, area or quality?: Decline (inferred)

Justification for trend: The introduction of *X. nubilus* seems to have originated the extirpation of *X. grohi* from most of the islands.

##### Habitat

Habitat importance: Major Importance

Habitats: 6. Rocky areas (e.g. inland cliffs, mountain peaks)13.1. Marine Coastal/Supratidal - Sea Cliffs and Rocky Offshore Islands

#### Habitat

Habitat importance: Major Importance

Habitats: 6. Rocky areas (e.g. inland cliffs, mountain peaks)13.1. Marine Coastal/Supratidal - Sea Cliffs and Rocky Offshore Islands

#### Ecology

Size: 5 mm

Generation length (yr): 1

Dependency of single sp?: No

Ecology and traits (narrative): This species is an ambush hunter of small insects over rocks and possibly low vegetation.

#### Threats

Justification for threats: The introduction of the invasive species *X. nubilus*, first detected in 2011, seems to have originated the extirpation of *X. grohi* from most of the islands. This invasive process may continue in the future to the current refuge of the endemic species, the steep coastal slopes.

##### Threats

Threat type: Ongoing

Threats: 8.1. Invasive and other problematic species, genes & diseases - Invasive non-native/alien species/diseases

#### Threats

Threat type: Ongoing

Threats: 8.1. Invasive and other problematic species, genes & diseases - Invasive non-native/alien species/diseases

#### Conservation

Justification for conservation actions: All of the species range is inside the Desertas Nature Reserve. The invasive *X. nubilus* should be erradicated from the island. As this task is probably impossible, ex-situ conservation with eventual re-introduction and recovery might be the only feasible measure to prevent the species extinction.

##### Conservation actions

Conservation action type: In Place

Conservation actions: 1.1. Land/water protection - Site/area protection

##### Conservation actions

Conservation action type: Needed

Conservation actions: 2.2. Land/water management - Invasive/problematic species control3.2. Species management - Species recovery3.3. Species management - Species re-introduction3.4. Species management - Ex-situ conservation

#### Conservation actions

Conservation action type: In Place

Conservation actions: 1.1. Land/water protection - Site/area protection

#### Conservation actions

Conservation action type: Needed

Conservation actions: 2.2. Land/water management - Invasive/problematic species control3.2. Species management - Species recovery3.3. Species management - Species re-introduction3.4. Species management - Ex-situ conservation

#### Other

##### Use and trade

Use type: International

##### Ecosystem services

Ecosystem service type: Very important

##### Research needed

Research needed: 1.2. Research - Population size, distribution & trends1.3. Research - Life history & ecology1.5. Research - Threats2.1. Conservation Planning - Species Action/Recovery Plan2.2. Conservation Planning - Area-based Management Plan3.1. Monitoring - Population trends

Justification for research needed: The current distribution of the species and possible threats from the invasive congener should be thoroughly studied. *X. grohi* should be the target of a species conservation plan with consequent area management actions. Monitoring of population trends should be conducted to confirm the species status.

#### Use and trade

Use type: International

#### Ecosystem services

Ecosystem service type: Very important

#### Research needed

Research needed: 1.2. Research - Population size, distribution & trends1.3. Research - Life history & ecology1.5. Research - Threats2.1. Conservation Planning - Species Action/Recovery Plan2.2. Conservation Planning - Area-based Management Plan3.1. Monitoring - Population trends

Justification for research needed: The current distribution of the species and possible threats from the invasive congener should be thoroughly studied. *X. grohi* should be the target of a species conservation plan with consequent area management actions. Monitoring of population trends should be conducted to confirm the species status.

#### Viability analysis

### Xysticus madeirensis

#### Species information

Scientific name: Xysticus madeirensis

Species authority: (Wunderlich, 1992)

Kingdom: Animalia

Phylum: Arthropoda

Class: Arachnida

Order: Araneae

Family: Thomisidae

Region for assessment: Global

#### Geographic range

Biogeographic realm: Palearctic

Countries: Portugal

Map of records (Google Earth): Suppl. material 54

Basis of EOO and AOO: Unknown

Basis (narrative): This species EOO and AOO are unknown.

Range description: This species is only known from Fajã da Nogueira at the northeastern region of Madeira Island in laurisilva forest (Fig. 3). A single female was recorded in November 1980. As this is an unusual season for most species it is possible the species was missed in recent collections due to its phenology.

#### New occurrences

#### Extent of occurrence

EOO (km2): Unknown

Trend: Unknown

Causes ceased?: Unknown

Causes understood?: Unknown

Causes reversible?: Unknown

Extreme fluctuations?: Unknown

#### Area of occupancy

Trend: Unknown

Causes ceased?: Unknown

Causes understood?: Unknown

Causes reversible?: Unknown

Extreme fluctuations?: Unknown

AOO (km2): Unknown

#### Locations

Number of locations: Unknown

Trend: Unknown

Extreme fluctuations?: Unknown

#### Population

Number of individuals: Unknown

Trend: Unknown

Causes ceased?: Unknown

Causes understood?: Unknown

Causes reversible?: Unknown

Extreme fluctuations?: Unknown

Population Information (Narrative): No population size estimates exist.

#### Subpopulations

Trend: Unknown

Extreme fluctuations?: Unknown

Severe fragmentation?: Unknown

#### Habitat

System: Terrestrial

Habitat specialist: Unknown

Habitat (narrative): Only known from laurisilva forest.

Trend in extent, area or quality?: Unknown

##### Habitat

Habitat importance: Major Importance

Habitats: 1.9. Forest - Subtropical/Tropical Moist Montane

#### Habitat

Habitat importance: Major Importance

Habitats: 1.9. Forest - Subtropical/Tropical Moist Montane

#### Ecology

Size: 6 mm

Generation length (yr): 1

Dependency of single sp?: No

Ecology and traits (narrative): As all congeners, certainly an ambush hunter feeding on small insects.

#### Threats

Justification for threats: Unknown threats.

##### Threats

Threat type: Past

Threats: 12. Other options - Other threat

#### Threats

Threat type: Past

Threats: 12. Other options - Other threat

#### Conservation

Justification for conservation actions: The known species range is inside the Madeira Natural Park.

##### Conservation actions

Conservation action type: In Place

Conservation actions: 1.1. Land/water protection - Site/area protection

#### Conservation actions

Conservation action type: In Place

Conservation actions: 1.1. Land/water protection - Site/area protection

#### Other

##### Use and trade

Use type: International

##### Ecosystem services

Ecosystem service type: Very important

##### Research needed

Research needed: 1.2. Research - Population size, distribution & trends1.3. Research - Life history & ecology1.5. Research - Threats

Justification for research needed: Basic research is needed on its distribution, ecology and possible threats throughout the range.

#### Use and trade

Use type: International

#### Ecosystem services

Ecosystem service type: Very important

#### Research needed

Research needed: 1.2. Research - Population size, distribution & trends1.3. Research - Life history & ecology1.5. Research - Threats

Justification for research needed: Basic research is needed on its distribution, ecology and possible threats throughout the range.

#### Viability analysis

### Zimirina lepida

#### Species information

Scientific name: Zimirina lepida

Species authority: (Blackwall, 1859)

Kingdom: Animalia

Phylum: Arthropoda

Class: Arachnida

Order: Araneae

Family: Prodidomidae

**Figure 8.**
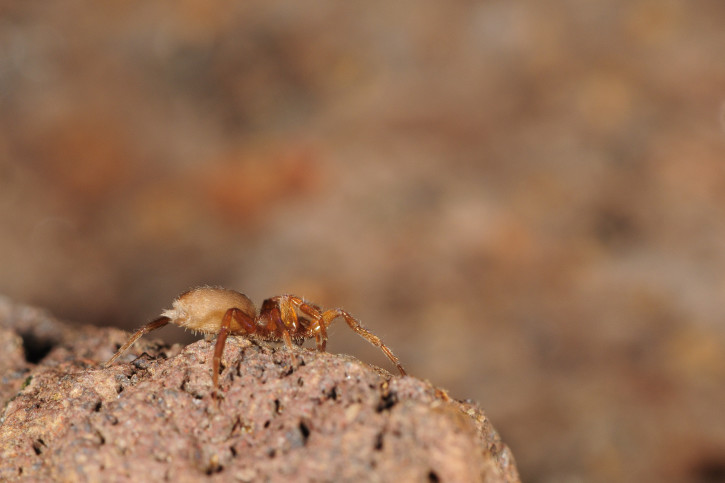
*Zimirina lepida* (Blackwall, 1859) (photo by Pedro Cardoso).

Figure(s) or Photo(s): Fig. 8

Region for assessment: Global

#### Geographic range

Biogeographic realm: Palearctic

Countries: Portugal

Map of records (Google Earth): Suppl. material 55

Basis of EOO and AOO: Species Distribution Model

Basis (narrative): Multiple collection sites are recorded for the species, mostly recent and in open grassland or shrubland (Wunderlich 1987, Wunderlich 1992, Crespo et al. 2009a, Crespo et al. 2009b, Crespo et al. 2013). It was possible to perform species distribution modeling to predict its potential range with confidence limits. See methods for details.

Min Elevation/Depth (m): 0

Max Elevation/Depth (m): 160

Range description: Known from all islands and few islets of Madeira and Selvagens archipelagos. Always at low altitude in open habitats such as grassland or shrubland.

#### New occurrences

#### Extent of occurrence

EOO (km2): 10682-10682-13343

Trend: Stable

Justification for trend: The species seems to be able to live on several habitat types, even close to human settlements.

Causes ceased?: Yes

Causes understood?: Yes

Causes reversible?: Yes

Extreme fluctuations?: No

#### Area of occupancy

Trend: Stable

Justification for trend: The species seems to be able to live on several habitat types, even close to human settlements.

Causes ceased?: Yes

Causes understood?: Yes

Causes reversible?: Yes

Extreme fluctuations?: No

AOO (km2): 32-188-432

#### Locations

Number of locations: 0

Justification for number of locations: No known threats to the species.

Trend: Stable

Extreme fluctuations?: No

#### Population

Number of individuals: Unknown

Trend: Stable

Justification for trend: The species seems to be able to live on several habitat types, even close to human settlements.

Causes ceased?: Yes

Causes understood?: Yes

Causes reversible?: Yes

Extreme fluctuations?: No

Population Information (Narrative): No population size estimates exist.

#### Subpopulations

Trend: Stable

Extreme fluctuations?: No

Severe fragmentation?: No

#### Habitat

System: Terrestrial

Habitat specialist: No

Habitat (narrative): Open grassland and shrubland, often in disturbed areas, close to or even inside houses.

Trend in extent, area or quality?: Stable

##### Habitat

Habitat importance: Major Importance

Habitats: 3.8. Shrubland - Mediterranean-type Shrubby Vegetation4.5. Grassland - Subtropical/Tropical Dry14.4. Artificial/Terrestrial - Rural Gardens

#### Habitat

Habitat importance: Major Importance

Habitats: 3.8. Shrubland - Mediterranean-type Shrubby Vegetation4.5. Grassland - Subtropical/Tropical Dry14.4. Artificial/Terrestrial - Rural Gardens

#### Ecology

Size: 2-3 mm

Generation length (yr): 1

Dependency of single sp?: No

Ecology and traits (narrative): Most probably an active hunter at ground level feeding on small size arthropods.

#### Threats

Justification for threats: Unknown threats.

##### Threats

Threat type: Past

Threats: 12. Other options - Other threat

#### Threats

Threat type: Past

Threats: 12. Other options - Other threat

#### Conservation

Justification for conservation actions: Part of the species range is inside several protected areas

##### Conservation actions

Conservation action type: In Place

Conservation actions: 1.1. Land/water protection - Site/area protection

#### Conservation actions

Conservation action type: In Place

Conservation actions: 1.1. Land/water protection - Site/area protection

#### Other

##### Use and trade

Use type: International

##### Ecosystem services

Ecosystem service type: Very important

##### Research needed

Research needed: 3.1. Monitoring - Population trends

Justification for research needed: Monitoring of population trends should be conducted to confirm species status.

#### Use and trade

Use type: International

#### Ecosystem services

Ecosystem service type: Very important

#### Research needed

Research needed: 3.1. Monitoring - Population trends

Justification for research needed: Monitoring of population trends should be conducted to confirm species status.

#### Viability analysis

## Discussion

Out of 56 species evaluated (*Hogna ingens* included), there is no reliable information on range and trends for 16 (29%). Among the 40 with reliable information, 29 are widespread, with an estimated EOO > 200 km^2^ and AOO > 60 km^2^. Most of these are restricted to the laurisilva forest that occupies 20% of the area of Madeira Island and which is well preserved and protected for the most part.

Seven species show a continuing decline in either range or population size. Their decline can be attributed to habitat destruction or degradation (*Centromerus anoculus* and *C. sexoculatus*, both exclusive to degraded caves), plant invasive species that reduce the habitat quality (*Dysdera portisancti* and *H. ingens*), wildfires at high mountain regions (*Mesiotelus maderianus*) and possible competition for resources from congeners (*Meta barreti* and *Xysticus grohi*).

The tetragnathid *M. barreti* is considered as possibly extinct due to the suspected impact of a competing species. Other than habitat destruction, competing species have been found to be the major menace for threatened spiders in Macaronesia (Cardoso et al. 2010). Competing species are almost always invasive taxa, but can also be native taxa that have spread above natural levels and to new areas due to imbalances in the communities. Such is the possible case of *Meta stridulans*, only recently described, yet now widespread throughout the native forest. We can also hypothesize that this species is in fact an invasive yet to be found on its original region, although this can only be speculated for now.

A few species are missing critical information to be able to assess their status, namely a couple that are only known from the high peaks of Madeira Island (*Drassodes rugichelis* and *Philodromus simillimus*). While all other habitats have been subject to multiple recent projects using standardized intensive sampling, the mountain regions have been sporadically sampled, rarely in recent times. This habitat, known to host multiple endemic species of other taxa such as snails and beetles, was subjected to catastrophic wildfires in 2010 that affected many threatened endemics, and should therefore be the focus for future work with spiders. Furthermore, it will also be important to assess the vulnerability of these mountain habitats and their associated spider fauna to climate change effects.

The effects of climate change, one of the prevailing threats accross the world affecting numerous species, was never studied for Madeira and Selvagens spiders. This is a known threat to Macaronesian bryophytes (Patiño et al. 2016) and Azorean spiders (Ferreira et al. 2016), and might affect many of the taxa assessed here. Yet, no good, high resolution future climatic data exists for Madeira and Selvagens, and a study into this has for now been postponed.

Among all the conservation measures suggested, the restoration of original habitat areas and control of invasive species are often a priority to guarantee the survival of threatened species. Many actions have been undertaken by the local authorities (Instituto das Florestas e da Conservação da Natureza) in Madeira and Selvagens archipelagos to control and eradicate invasive plants and mammals jointly with habitat restoration programs. However, it is important not only to ensure the continuity of those actions, but also to monitor their effectiveness by assessing population changes in selected groups like spiders as they already proved to be efficient and effective bioindicators in many terrestrial ecosystems (Bonte et al. 2002, Scott et al. 2006), including oceanic islands (Cardoso et al. 2010, Cardoso et al. 2013). For a few species (*Dysdera portisancti*, *Meta barreti*, *Misumena nigromaculata*, *Oecobius selvagensis* and *Xysticus grohi*), ex-situ conservation, recovery and possible re-introduction could be a last resort or insurance against extinction, if this has not occurred yet.

The network of protected areas in Madeira and Selvagens archipelagos is extensive and covers most of the areas known to harbour higher values of species richness and endemism (Boieiro et al. 2015). A clear gap is however evident in Porto Santo (main island) where some important areas for nature conservation are in urgent need to benefit from legal protection. The selection of new areas for nature conservation in Madeira archipelago must take in consideration the spider fauna, but also other invertebrate groups, since they accomodate the largest fractions of the biological diversity, endemicity and vulnerability to extinction.

In conclusion, although most endemic spiders from Madeira and Selvagens archipelagos are in a favourable situation due to the good condition and protection of the laurisilva forests where many live, there are a number of species requiring urgent attention and protection measures. These include the few cave and mountain-restricted species or threatened by competing congeners or invasive plants. Extending current protected areas, restoring original habitats of threatened species and control invasive taxa are still a priority.

Discussion

## Supplementary Material

Supplementary material 1 Distribution of *Araneus hortensis* (Blackwall, 1859) Data type: Distribution
File: oo_146332.kml Cardoso, P.

Supplementary material 2 Distribution of *Arctosa maderana* Roewer, 1960 Data type: Distribution
File: oo_146336.kml Cardoso, P.

Supplementary material 3 Distribution of *Centromerus anoculus* Wunderlich, 1995 Data type: Distribution
File: oo_146706.kml Cardoso, P.

Supplementary material 4 Distribution of *Centromerus sexoculatus* Wunderlich, 1992 Data type: Distribution
File: oo_146707.kml Cardoso, P.

Supplementary material 5 Distribution of *Centromerus variegatus* Denis, 1962 Data type: Distribution
File: oo_146340.kml Cardoso, P.

Supplementary material 6 Distribution of *Ceratinopsis infuscata* (Denis, 1962) Data type: Distribution
File: oo_146342.kml Cardoso, P.

Supplementary material 7 Distribution of *Dipoenata longitarsis* (Denis, 1962) Data type: Distribution
File: oo_146343.kml Cardoso, P.

Supplementary material 8 Distribution of *Drassodes rugichelis* Denis, 1962 Data type: Distribution
File: oo_146345.kml Cardoso, P.

Supplementary material 9 Distribution of *Dysdera aneris* Macías-Hernández & Arnedo, 2010 Data type: Distribution
File: oo_147143.kml Cardoso, P.

Supplementary material 10 Distribution of *Dysdera coiffaiti* Denis, 1962 Data type: Distribution
File: oo_146347.kml Cardoso, P.

Supplementary material 11 Distribution of Dysdera diversa Blackwall, 1862 Data type: Distribution
File: oo_146348.kml Cardoso, P.

Supplementary material 12 Distribution of *Dysdera portisancti* Wunderlich, 1995 Data type: Distribution
File: oo_146350.kml Cardoso, P.

Supplementary material 13 Distribution of *Dysdera vandeli* Denis, 1962 Data type: Distribution
File: oo_146352.kml Cardoso, P.

Supplementary material 14 Distribution of *Echemus modestus* Kulczynski, 1899 Data type: Distribution
File: oo_146354.kml Cardoso, P.

Supplementary material 15 Distribution of *Frontinellina dearmata* (Kulczynski, 1899) Data type: Distribution
File: oo_146355.kml Cardoso, P.

Supplementary material 16 Distribution of *Frontiphantes fulgurenotatus* (Schenkel, 1938) Data type: Distribution
File: oo_146359.kml Cardoso, P.

Supplementary material 17 Distribution of *Hahnia insulana* Schenkel, 1938 Data type: Distribution
File: oo_146358.kml Cardoso, P.

Supplementary material 18 Distribution of *Hogna biscoitoi* Wunderlich, 1992 Data type: Distribution
File: oo_146360.kml Cardoso, P.

Supplementary material 19 Distribution of *Hogna heeri* (Thorell, 1875) Data type: Distribution
File: oo_146364.kml Cardoso, P.

Supplementary material 20 Distribution of *Hogna insularum* (Kulczynski, 1899) Data type: Distribution
File: oo_146367.kml Cardoso, P.

Supplementary material 21 Distribution of *Hogna maderiana* (Walckenaer, 1837) Data type: Distribution
File: oo_146537.kml Cardoso, P.

Supplementary material 22 Distribution of *Hogna nonannulata* Wunderlich, 1995 Data type: Distribution
File: oo_146370.kml Cardoso, P.

Supplementary material 23 Distribution of *Hogna schmitzi* Wunderlich, 1992 Data type: Distribution
File: oo_146774.kml Cardoso, P.

Supplementary material 24 Distribution of *Lathys affinis* (Blackwall, 1862) Data type: Distribution
File: oo_146373.kml Cardoso, P.

Supplementary material 25 Distribution of *Lepthyphantes impudicus* Kulczynski, 1909 Data type: Distribution
File: oo_146374.kml Cardoso, P.

Supplementary material 26 Distribution of *Lepthyphantes lundbladi* Schenkel, 1938 Data type: Distribution
File: oo_146375.kml Cardoso, P.

Supplementary material 27 Distribution of *Lepthyphantes mauli* Wunderlich, 1992 Data type: Distribution
File: oo_146376.kml Cardoso, P.

Supplementary material 28 Distribution of *Macaroeris desertensis* Wunderlich, 1992 Data type: Distribution
File: oo_146377.kml Cardoso, P.

Supplementary material 29 Distribution of *Macarophaeus cultior* (Kulczynski, 1899) Data type: Distribution
File: oo_146378.kml Cardoso, P.

Supplementary material 30 Distribution of *Mesiotelus maderianus* Kulczynski, 1899 Data type: Distribution
File: oo_146380.kml Cardoso, P.

Supplementary material 31 Distribution of *Meta barreti* Kulczynski, 1899 Data type: Distribution
File: oo_146381.kml Cardoso, P.

Supplementary material 32 Distribution of *Meta stridulans* Wunderlich, 1987 Data type: Distribution
File: oo_146382.kml Cardoso, P.

Supplementary material 33 Distribution of *Misumena nigromaculata* Denis, 1963 Data type: Distribution
File: oo_146383.kml Cardoso, P.

Supplementary material 34 Distribution of *Oecobius minor* Kulczynski, 1909 Data type: Distribution
File: oo_152599.kml Cardoso, P.

Supplementary material 35 Distribution of *Oecobius selvagensis* Wunderlich, 1995 Data type: Distribution
File: oo_146387.kml Cardoso, P.

Supplementary material 36 Distribution of *Parapelecopsis mediocris* (Kulczynski, 1899) Data type: Distribution
File: oo_146395.kml Cardoso, P.

Supplementary material 37 Distribution of *Philodromus insulanus* Kulczynski, 1905 Data type: Distribution
File: oo_146396.kml Cardoso, P.

Supplementary material 38 Distribution of Philodromus simillimus Denis, 1962 Data type: Distribution
File: oo_146398.kml Cardoso, P.

Supplementary material 39 Distribution of *Pholcus dentatus* Wunderlich, 1995 Data type: Distribution
File: oo_146399.kml Cardoso, P.

Supplementary material 40 Distribution of *Pholcus madeirensis* Wunderlich, 1987 Data type: Distribution
File: oo_146402.kml Cardoso, P.

Supplementary material 41 Distribution of *Pholcus magnus* Wunderlich, 1987 Data type: Distribution
File: oo_146404.kml Cardoso, P.

Supplementary material 42 Distribution of *Pholcus parvus* Wunderlich, 1987 Data type: Distribution
File: oo_146406.kml Cardoso, P.

Supplementary material 43 Distribution of *Pholcus silvai* Wunderlich, 1995 Data type: Distribution
File: oo_152600.kml Cardoso, P.

Supplementary material 44 Distribution of *Prinerigone pigra* (Blackwall, 1862) Data type: Distribution
File: oo_146411.kml Cardoso, P.

Supplementary material 45 Distribution of *Rugathodes madeirensis* Wunderlich, 1987 Data type: Distribution
File: oo_146412.kml Cardoso, P.

Supplementary material 46 Distribution of *Scotognapha paivani* (Blackwall, 1864) Data type: Distribution
File: oo_146413.kml Cardoso, P.

Supplementary material 47 Distribution of *Spermophorides selvagensis* Wunderlich, 1992 Data type: Distribution
File: oo_146414.kml Cardoso, P.

Supplementary material 48 Distribution of *Steatoda distincta* (Blackwall, 1859) Data type: Distribution
File: oo_146415.kml Cardoso, P.

Supplementary material 49 Distribution of *Tenuiphantes tenebricoloides* (Schenkel, 1938) Data type: Distribution
File: oo_146416.kml Cardoso, P.

Supplementary material 50 Distribution of *Trogloneta madeirensis* Wunderlich, 1987 Data type: Distribution
File: oo_146417.kml Cardoso, P.

Supplementary material 51 Distribution of *Turinyphia maderiana* (Schenkel, 1938) Data type: Distribution
File: oo_146418.kml Cardoso, P.

Supplementary material 52 Distribution of *Typhochrestus madeirensis* Crespo, 2013 Data type: Distribution
File: oo_149592.kml Cardoso, P.

Supplementary material 53 Distribution of *Xysticus grohi* (Wunderlich, 1992) Data type: Distribution
File: oo_152582.kml Cardoso, P.

Supplementary material 54 Distribution of *Xysticus madeirensis* (Wunderlich, 1992) Data type: Distribution
File: oo_146421.kml Cardoso, P.

Supplementary material 55 Distribution of *Zimirina lepida* (Blackwall, 1859) Data type: Distribution
File: oo_146423.kml Cardoso, P.

## Figures and Tables

**Figure 5.**
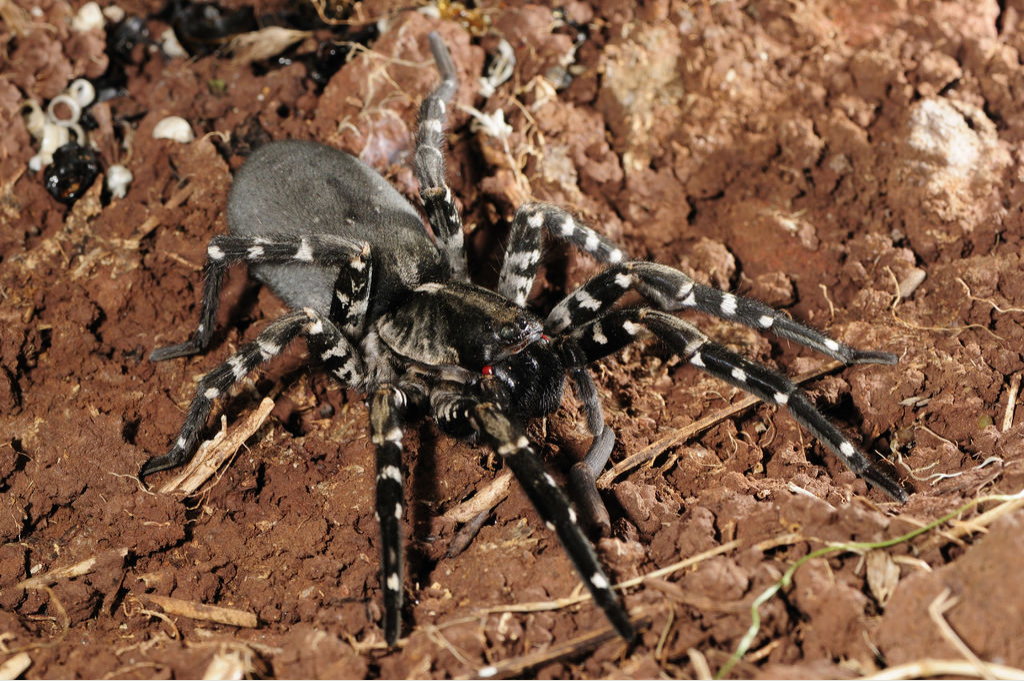
*Hogna ingens* (Blackwall, 1857) adult female (photo by Pedro Cardoso).
